# Variability in Phelan-McDermid Syndrome in a Cohort of 210 Individuals

**DOI:** 10.3389/fgene.2022.652454

**Published:** 2022-04-12

**Authors:** Julián Nevado, Sixto García-Miñaúr, María Palomares-Bralo, Elena Vallespín, Encarna Guillén-Navarro, Jordi Rosell, Cristina Bel-Fenellós, María Ángeles Mori, Montserrat Milá, Miguel del Campo, Pilar Barrúz, Fernando Santos-Simarro, Gabriela Obregón, Carmen Orellana, Harry Pachajoa, Jair Antonio Tenorio, Enrique Galán, Juan C. Cigudosa, Angélica Moresco, César Saleme, Silvia Castillo, Elisabeth Gabau, Luis Pérez-Jurado, Ana Barcia, Maria Soledad Martín, Elena Mansilla, Isabel Vallcorba, Pedro García-Murillo, Franco Cammarata-Scalisi, Natálya Gonçalves Pereira, Raquel Blanco-Lago, Mercedes Serrano, Juan Dario Ortigoza-Escobar, Blanca Gener, Verónica Adriana Seidel, Pilar Tirado, Pablo Lapunzina, Spanish PMS Working Group, Mena Rocío

**Affiliations:** ^1^ Instituto de Genética Médica y Molecular (INGEMM)-IdiPAZ, Hospital Universitario La Paz, Madrid, Spain; ^2^ CIBERER, Centro de Investigación Biomédica en Red de Enfermedades Raras, ISCIII, Madrid, Spain; ^3^ ITHACA-European Reference Network, Hospital La Paz, Madrid, Spain; ^4^ Hospital Virgen de la Arrixaca, Murcia, Spain; ^5^ Hospital Son Espases, Palma de Mallorca, Spain; ^6^ Departamento de Investigación y Psicología en Educación, Facultad de Educación, UCM, Madrid, Spain; ^7^ CEE Estudio-3, Afanias, Madrid, Spain; ^8^ Hospital Clinic, Barcelona, Spain; ^9^ Hospital Vall D’Hebron, Barcelona, Spain; ^10^ Hospìtal Juan P. Garrahan, Buenos Aires, Argentina; ^11^ Hospital La Fé, Valencia, Spain; ^12^ Universidad Icesi, Cali, Colombia; ^13^ Hospital Materno-Infantil Infanta Cristina, Badajoz, Spain; ^14^ NIM-Genetics Madrid, Alcobendas, Spain; ^15^ Maternity Nuestra Señora de la Merced, Tucumán, Argentina; ^16^ Sección Genética, Hospital Clínico Universidad de Chile, Santiago, Chile; ^17^ Clínica Alemana, Santiago, Chile; ^18^ Corporación Sanitaria Parc Taulí, Barcelona, Spain; ^19^ Servicio de Genética, Instituto de Investigaciones Médicas Hospital del Mar (IMIM)/Universitat Pompeu Fabra, Barcelona, Spain; ^20^ Hospital Universitario Virgen del Rocío, Sevilla, Spain; ^21^ Unidad de Genética, Hospital Virgen de la Salud, Toledo, Spain; ^22^ Servicio de Pediatría, Hospital Regional de Antofagasta, Antofagasta, Chile; ^23^ Clínica Reprodutiva NIDUS, Juiz de Fora, Brasil; ^24^ Servicio de Neuropediatría, Hospital Universitario Central de Asturias, Oviedo (Asturias), Spain; ^25^ Unidad de Neuropediatría, Hospital San Joan de Deu, Barcelona, Spain; ^26^ Hospital Universitario de Cruces, Bilbao, Spain; ^27^ Servicio de Genética Clínica, Hospital Universitario Gregorio Marañon, Madrid, Spain; ^28^ Servicio de Neuropediatría, Hospital Universitario La Paz, Madrid, Spain

**Keywords:** autistic behavior, 22q13 deletion syndrome, Phelan-McDermid syndrome (PMS), *SHANK3*, subtelomeric deletion syndrome, intellectual disabilities (ID)

## Abstract

Phelan-McDermid syndrome (PMS, OMIM# 606232) results from either different rearrangements at the distal region of the long arm of chromosome 22 (22q13.3) or pathogenic sequence variants in the *SHANK3* gene. *SHANK3* codes for a structural protein that plays a central role in the formation of the postsynaptic terminals and the maintenance of synaptic structures. Clinically, patients with PMS often present with global developmental delay, absent or severely delayed speech, neonatal hypotonia, minor dysmorphic features, and autism spectrum disorders (ASD), among other findings. Here, we describe a cohort of 210 patients with genetically confirmed PMS. We observed multiple variant types, including a significant number of small deletions (<0.5 Mb, 64/189) and *SHANK3* sequence variants (21 cases). We also detected multiple types of rearrangements among microdeletion cases, including a significant number with post-zygotic mosaicism (9.0%, 17/189), ring chromosome 22 (10.6%, 20/189), unbalanced translocations (*de novo* or inherited, 6.4%), and additional rearrangements at 22q13 (6.3%, 12/189) as well as other copy number variations in other chromosomes, unrelated to 22q deletions (14.8%, 28/189). We compared the clinical and genetic characteristics among patients with different sizes of deletions and with *SHANK3* variants*.* Our findings suggest that *SHANK3* plays an important role in this syndrome but is probably not uniquely responsible for all the spectrum features in PMS. We emphasize that only an adequate combination of different molecular and cytogenetic approaches allows an accurate genetic diagnosis in PMS patients. Thus, a diagnostic algorithm is proposed.

## Introduction

In the past 15–20 years, the increasing use of genome-wide telomere screening by fluorescence *in situ* hybridization (FISH), multiplex ligation-dependent probe amplification (MLPA, Schouten et al., 2002), and more recently chromosome microarrays (CMA) has provided evidence of the presence of subtle abnormalities involving telomeres in around 5% (range, 2%–30%) of patients with intellectual disability (ID) (Anderlid et al., 2002a; Shao et al., 2008). In the evaluation of ID patients, deletion of 22q13.3, also known as Phelan-McDermid syndrome (PMS; OMIM#:606232), is one of the most common subtelomeric deletions after 1p36.3 deletion syndrome (Heilstedt et al., 2003; Delahaye et al., 2009). PMS usually results from either the loss of genetic material at the distal region of the long arm of chromosome 22 (including *SHANK3*) or pathogenic sequence variants in *SHANK3*.

SHANK3 plays a central role in forming the postsynaptic environment, integrating the protein network of glutamate receptors at postsynaptic density and the maintenance of synaptic structures (Boeckers, 2006; Durand et al., 2007). Deletion sizes vary considerably among PMS individuals, ranging from intragenic deletions in the *SHANK3* gene (∼13 Kb) to around 9 Mb (Bonaglia et al., 2011; Phelan et al., 2018). The deletion occurs with similar frequency in male and female. *SHANK3* haploinsufficiency is proposed to be responsible for the major neurological features of the 22q13 deletion syndrome (Bonaglia M. C. et al., 2001; Anderlid et al., 2002b; Wilson et al., 2003; Durand et al., 2007; Phelan et al., 2018) and recently has also been shown to be involved in additional clinical features of the syndrome in humans (De Rubeis et al., 2018) and mice (Sauer et al., 2019). However, interstitial deletions disrupting the 22q13.3 band, not including *SHANK3* (Wilson et al., 2008; Disciglio et al., 2014; Ha et al., 2017), are also reported. The clinical features in these patients overlap those of PMS, raising debate about whether they can be diagnosed as having PMS.

Although many PMS patients have been diagnosed worldwide, most of the individuals included in previous genotype-phenotype analyses had microdeletions (Cusmano-Ozog et al., 2007; Dhar et al., 2010; Sarasua et al., 2011; Soorya et al., 2013; Sarasua et al., 2014a,b; Tabet et al., 2017; Samogy-Costa et al., 2019). Indeed, the proportion of patients with *SHANK3* variants in previous data is 3%–25% (Phelan et al., 2018; De Rubeis et al., 2018; and ClinVar, Varsome, LOVD databases) or 8.6% in the PMS International Registry (among genetically confirmed cases; Kolevzon et al., 2019). Thus, PMS seems to be underdiagnosed, and its exact prevalence in is unknown.

Here, we describe the clinical and molecular data of one of the largest cohorts of patients with confirmed genetic diagnosis of PMS, most of them with microdeletions (189/210, 90%) and 21 with *SHANK3* sequence variants (10%). High-resolution CMA, cytogenetic, and MLPA techniques were necessary to delineate the size and gene content of the deletions and to identify additional rearrangements. Exome and/or target panel sequencing analysis of *SHANK3* were preferentially applied for *SHANK3* sequence variant analysis.

## Material and Methods

### Subjects

Between 2008 and 2020, 242 patients with confirmed PMS, mostly nonrelated (except for four individuals from two families), were recruited for this study in collaboration with the Spanish PMS Association and the Argentinean PMS Group. Twenty-eight of these had incomplete clinical or molecular data and were not included in this study. Three were excluded because they carried deletions at 22q13.33 nearby to *SHANK3* but not including this gene, and one had an intragenic *SHANK3* duplication and was also excluded because, at this time, we are not able to confirm that the duplication is in tandem and disrupts *SHANK3*. Thus, 210 individuals constituted the final cohort (Supplementary Figure S1).

Most of the DNA samples from these patients were extracted and analyzed at INGEMM (Madrid, Spain). A minority of them had been previously analyzed outside of our institution by high-resolution CMA or next generation sequencing (NGS). The patients’ clinical information was obtained from the referring physicians and/or their clinical geneticists and compiled in two questionnaires. Data were completed by reviewing medical records and parents’ interviews. Parents or guardians provided informed consent. The Institutional Review Board of Hospital Universitario La Paz approved the study (PI: 2735 HULP, Madrid. Spain).

## Methods

### Karyotyping and FISH

Cytogenetic analyses were performed on GTG-banded metaphases at a resolution of about 550 bands according to standard laboratory protocols using Chromosome Kit P (Euroclone, Siziano PV, Italy). FISH was performed according to standard laboratory protocols using the subtelomeric 22q13 probe (D22S1056, Kreatech Biotechnology B.V, Amsterdam, Netherlands) or the DiGeorge/VCFS probe mixture (Vysis Inc., IL, United States), containing a control probe in *ARSA* that maps to the 22q13.3 region. In some cases, the probe N25/N85A3 (Cytocell, Cambridge, United Kingdom) within the *SHANK3* locus was also used.

### Parental Origin Analysis

We used highly polymorphic short tandem repeats (D22S1169, D22S1149, D22S444, D22S1170, D22S295, and D22S1141) mapping within the *SHANK3* gene and around it to evaluate parental segregation. The forward primers were synthesized and labeled with fluorescein-amidite (Sigma-Aldrich, St. Louis, MO, United States), whereas the reverse primers were not labeled (primer sequences are available upon request). The region amplified by these primers depended on the number of repeats. Capillary electrophoresis (Applied Biosystems Genetic Analyzer System 3130) was used to detect the length of the fragments (Thermo Fisher, CA, United States).

### MLPA Probe Kits

We used several MLPA-Salsa kits in this study (MRC-Holland, Amsterdam, Netherlands). For patients referred to rule out subtelomeric rearrangements in the first years of the study, MLPA kits P036 and P070 were used. DNA samples of all patients with 22q13 deletions were further characterized with the specific MLPA P188 and P339 probe mixes for PMS (MRC-Holland). Both kits contain 34 sequence probes on chromosome 22q13 and control ones for other chromosomes (12 and 9, respectively). The majority of the 22q13 probes (22/34) are in the 1 Mb terminal region of the long arm (P188) and include multiple probes within *SHANK3* (P339). Data analyses were performed according to the protocols supplied by the providers defining relative probe signals by dividing each measured peak area by the sum of all peak areas of the control probes of that sample. Each peak’s relative probe area ratio was then compared to a DNA control sample (Promega, United Kingdom), using Coffalysser.net (MRC- Holland).

### Chromosome Microarray Analysis (CMA)

Different array platforms were used in this study: *1*) a clinical 60K-array CGH (INGEMM, KaryoArray-^®^, Vallespin et al., 2013) in 72 of 189 patients; *2*) a high-resolution customized- 60K aCGH (INGEMM custom design, not published) at 22q13.3 in 30 of 189 patients; *3*) different custom or commercial CGH-microarrays with a variety of resolutions in 59 patients (Supplementary Figure S2A); *4*) a genome-wide scan of 850,00 tag SNPs (Illumina Infinium CytoSNP-850K BeadChip) in 56 patients (Supplemental Data, Supplementary Figure 2B) at INGEMM; and *5*) a genome-wide scan of 750,00 tag SNPs (Affymetrix, ThermoFisher Scientific, Waltham, MA, United States) in 11 patients. Arrays in 1–3 were analyzed with Cytogenomics software (Agilent Corporation; Santa Clara, CA, United States). Image data from 4 were analyzed using the Chromosome Viewer tool contained in the Genome Studio package (Illumina, San Diego, CA, United States). In Chromosome Viewer, gene call scores <0.15 at any locus were considered “no calls.” In addition, allele frequency analysis was applied for all SNPs. For the analysis of 5, the ChAS software (Affymetrix, Thermo-Fisher Scientific, Waltham, MA, United States) was used.

All genomic coordinates were established according to the 2009 human genome build 19 (GRCh37/NCBI build 37.1). Deletion coordinates were plotted using the University of California at Santa Cruz Genome Browser (http://genome.ucsc.edu/).

### *SHANK3* Sequencing Analysis

These studies were performed either at INGEMM or outside of our institution, using different NGS approaches, all under the manufacturer’s guidelines: *1*) exome sequencing by trio analysis using the Agilent SureSelect XT clinical research exome (Agilent Tech) and IDT Technologies (Coralville, IA, United States); *2*) singleton exome sequencing CentoXome Gold^®^, and NOVAGENE (Agilent all exon V6) and MedExome, Q-Genomics (Barcelona, Spain); and *3*) a customized gene panel of specific genes related to ID or/and autism (Agilent-based Technologies). Most samples (98%) were run in Illumina instruments (such as Nextseq500; Miseq, Hiseq 2000/4000; Illumina, San Diego, CA, United States). Classification of the variants follows ACMG/AMP criteria (Richards et al., 2015), using VarSome 10.2 as a web source.

### Validation of Global Functional Assessment of the Patients (GFAP)

We estimated an individual severity score in our cohort using different features taken from the questionnaires and weighed them by Human Ontology Phenotype (HPO) term frequencies on a numerical scale of core features of the syndrome. The GFAP was constructed as follows: items with a frequency between 0% and 20%, 1 point; between 20% and 35%, 2 points; between 35% and 70%, 5 points, and >70%, 10 points. Principal component analysis (PCA) was used to validate the GFAP construct, containing Kaiser-Meyer-Olkin’s measure and Barlett’s test.

### Statistical Analysis

Statistical analyses were performed with SPSS version 25 (IBM Corporation, Chicago, IL, United States). Descriptive analysis included mean ± SD for continuous variables and frequency tables for categorical variables (Table 1). The categorical variables were taken from our two questionnaires curated from medical records and were expressed as “1” (condition present at some point) or “0” (condition not present at any time). Correlation associations were calculated using Pearson’s linear correlation coefficient (continuous variables) or Spearman’s Rho and Kendall’s tau_b (categorical variables). Comparisons between two groups were performed either with Student’s *t*-test (for continuous variables) or chi-square tests (for categorical ones). For more than two groups, ANOVA (followed by Bonferroni’s or T3-Dunnett *post hoc* tests) were run for continuous variables and *z*-tests between column proportions for categorical variables. Ward’s minimum variance method was the criterion used in hierarchical cluster analysis, and the number of clusters was selected using the Bayesian information criterion (BIC) or Akaike information criterion (AIC). A *p*-value lower than .05 was considered to indicate a statistically significant difference.

**TABLE 1:** Descriptive statistics and frequencies of variables used in the study of 22q13.3 microdeletions and *SHANK3* variants. a) Categorical variables

		Deletions		*SHANK3* variants	
		Frequency	Percentage	Frequency	Percentage
Sex	Male	85	44.7	13	61.9
	Female	105	55.3	8	38.1
	Total	190	100	21	100
Growth	Centile ≤3	23	12.1	0	0
	Normal	105	56.3	16	88.9
	Centile ≥95	60	31.6	2	11.1
	Total	188	100	18	100
Walk independently	≤15 months	50	26.3	13	72.2
	>15 months	139	73.7	5	27.8
	Total	189	100	18	100
Delayed/absent speech	No words	65/181	36	5/18	27.7
	Some words, 10–20	70/181	39.6	8/18	44.4
	Many words, and ability to make sentences	46/181	25.4	5/18	27.7
	Total	181	100	18	100
Hypotonia	No	45	24.1	8	38
	Yes	142	75.9	13	62
	Total	187	100	21	100
Behavior abnormalities (e.g., stereotypies, manic behavior)	No	39	20.9	1	5.9
	Yes	148	79.1	20	94.1
	Total	187	100	21	100
Regressions	No	98	52.1	10	52.6
	Yes	90	47.9	9	47.4
	Total	188	100	19	100
Seizures	No	129	69	16	84.2
	Yes	58	31	3	15.8
	Total	187	100	19	100
High pain threshold	No	62	33.2	4	21
	Yes	125	66.8	15	79
	Total	187	100	19	100
Decreased perspiration	Yes	99	52.7	5	31.2
	Normal	77	42.4	11	68.8
	Increased	11	5.9	0	0
	Total	187	100	16	100
Microcephaly	Normal	151	81.1	16	88.9
	Yes	37	18.9	2	11.1
	Total	188	100	18	100
Macrocephaly	Normal	139	73.9	14	76.8
	Yes	49	26.1	4	22.2
	Total	188	100	18	100
	No	150	79.8	16	94.1
Dolicocephaly	Yes	38	20.2	1	5.9
	Total	188	100	17	100
	No	161	86.1	16	88.9
Flat midface	Yes	26	13.9	2	11.1
	Total	187	100	18	100
	No	134	71.3	16	88.9
Epicanthal folds	Yes	54	28.7	2	11.1
	Total	188	100	18	100
	No	138	73.8	16	88.9
Strabismus	Yes	49	26.2	2	11.1
	Total	187	100	18	100
	No	153	81.8	15	88.2
Ptosis	Yes	34	18.2	2	11.8
	Total	187	100	17	100
	No	80	42.8	8	44.4
Long eyelashes	Yes	107	57.2	10	55.6
	Total	187	100	18	100
	No	113	60.4	16	94.1
Full eyebrow	Yes	74	39.6	1	5.9
	Total	187	100	17	100
	No	144	75.8	17	94.5
Full/puffy eyelids	Yes	43	22.6	1	5.5
	Total	187	100	18	100
	No	143	77	17	94.5
Deep set eyes	Yes	44	23	1	5.5
	Total	187	100	18	100
	No	82	43.9	12	60
Wide nasal bridge	Yes	105	56.1	8	40
	Total	187	100	20	100
	No	79	42.2	11	61.1
Bulbous nose	Yes	108	57.8	7	38.9
	Total	187	100	18	100
	No	102	54	11	57.9
Ear anomalies	Yes	86	46	8	42.1
	Total	188	100	19	100
	No	145	77.5	15	79
Full/puffy cheeks	Yes	42	22.5	4	21
	Total	187	100	19	100
	No	99	52.9	15	83.3
Widely spaced teeth/malocclusion	Yes	88	47.1	3	16.7
	Total	187	100	18	100
	No	78	41.7	7	38.9
Pointed chin	Yes	109	58.3	11	61.1
	Total	187	100	18	100
	No	136	72.7	17	94.5
Toe syndactyly	Yes	51	27.3	1	5.5
	Total	187	100	18	100
	No	86	45.3	9	52.9
Large, fleshy hands	Yes	101	53.2	8	47.1
	Total	187	100	17	100
	No	152	80	16	94.1
Fifth finger clinodactyly	Yes	35	18.4	1	5.9
	Total	187	100	17	100
	No	111	59.4	11	61.1
Hypoplastic/dysplastic nails	Yes	76	40.6	7	38.9
	Total	187	100	18	100
Main reason for genetic consultation	DD	102	54	5	29.4
	ASD	26	13.8	8	47.1
	Dysmorphic features	7	3.7	0	0
	ID	17	9.0	1	5.9
	Hypotonia	16	8.5	0	0
	Language problems	8	4.2	3	17.6
	Other	13	6.8	0	0
	Total	189	100	17	100
	DD	66	34.9	2	11.8
Second reason for genetic consultation	ASD	26	13.8	4	23.5
	Dysmorphic features	9	4.6	1	5.9
	ID	27	14.3	1	5.9
	Hypotonia	19	10.1	0	0
	Language problems	26	13.8	9	52.9
	Other	16	8.5	0	0
	Total	189	100	17	100
	No	159	84.1	16	94.1
Cardiac anomalies	Yes	30	15.9	1	5.9
	Total	189	100	17	100
	No	146	78.1	13	76.5
Ophthalmologic anomalies	Yes	41	21.9	4	23.5
	Total	187	100	17	100
	No	161	86.1	8	47.1
Sphincter control	Yes	26	13.9	9	52.9
	Total	187	100	17	100
	No	146	77.7	16	94.1
Renal and urogenital anomalies	Yes	42	22.2	1	5.9
	Total	188	100	17	100
	No	171	91.0	16	88.9
Lip/palate abnormalities	Yes	17	9.0	2	11.1
	Total	188	100	18	100
	No	142	75.9	8	42.1
Sleeping disorders	Yes	45	24.1	11	57.9
	Total	187	100	19	100
	No	146	77.7	13	76.5
Skin anomalies	Yes	42	22.2	4	23.5
	Total	188	100	17	100
	No	157	83.9	13	72.2
Recurrent infections	Yes	30	16.1	5	27.8
	Total	187	100	18	100
	No	175	93.6	17	100
Herniae	Yes	12	6.4	0	0
	Total	187	100	17	100
	No	184	97.9	17	100
Obesity	Yes	4	2.1	0	0
	Total	188	100	17	100
	No	167	89.3	14	82.2
Hearing problems	Yes	20	10.7	3	17.8
	Total	187	100	17	100
	No	169	90.4	17	100
Lymphedema	Yes	18	9.6	0	0
	Total	187	100	17	100
	No	153	81.8	13	72.2
Gastrointestinal problems	Yes	34	18.2	5	27.8
	Total	187	100	18	100
	Not performed	92	49.2	8	38.1
Brain MRI	Normal	59	31.6	11	52.4
	With abnormalities	36	19.2	2	9.5
	Total	187	100	21	100
	No	81	43.3	9	50
Poor visual contact	Yes	106	56.7	9	50
	Total	187	100	18	100
	No	117	62.6	11	61.1
Biting	Yes	70	37.4	7	38.9
	Total	186	100	18	100
	No	126	67.4	6	33.3
Very sensitive to touch	Yes	61	32.6	12	66.7
	Total	187	100	18	100
	No	118	63.1	11	61.1
Uncontrolled laughter	Yes	69	36.9	7	38.9
	Total	187	100	18	100
	No	90	48.1	10	52.6
Impulsive	Yes	97	51.9	9	47.4
	Total	187	100	19	100
	No	117	63.1	13	72.2
Excessive yelling	Yes	69	36.9	5	27.8
	Total	186	100	18	100
	No	145	77.2	13	76.5
Hair pulling	Yes	42	22.6	4	23.5
	Total	187	100	17	100
	No	144	77	13	76.5
Skin picking	Yes	43	23	4	23.5
	Total	187	100	17	100
	No	161	86.1	13	76.5
Nonstop crying	Yes	26	13.9	4	23.5
	Total	187	100	17	100
	No	151	80.8	17	89.5
Aggressive behavior	Yes	36	19.2	2	10.5
	Total	187	100	19	100
	No	125	66.9	12	66.7
Tongue thrusting, sticking out	Yes	62	33.1	6	33.3
	Total	187	100	18	100
	No	89	47.6	4	22.2
Abnormal emotional response	Yes	98	52.4	14	77.8
	Total	187	100	18	100
Formal ASD evaluation	Not performed	152	81.3	13	62
	Normal	13	6.9	1	4.8
	ASD diagnosis^a^	22	11.8	7	33.2
	Total	187	100	21	100

a ASD diagnosis according to the psychiatrists of the referring institutions.

## Results

### Cohort

Individuals (*n* = 210), all previously nonreported, are mostly from Spain, all over the country (*n* = 178), and from South America (*n* = 32), mainly from Argentina (Supplementary Figure S1). The female/male ratio, 1.12:1 (111/99), was similar to previous reports, and ages ranged from birth to 62 years. Descriptive statistics (for continuous variables) and frequencies (for categorical items) are shown in Table 1. The majority of individuals with PMS in our cohort are of pediatric age (between 0 and 16 years old, 146 patients; 69.5%). The mean age at diagnosis was around 6 years old for deletions (Table 1b) and around 8 years for the group with sequence variants in *SHANK3*. The mean age at evaluation were 12.44 ± 8.7 years and 10.99 ± 5.95 years for deletions and *SHANK3* sequence variants, respectively (Table 1b).

### Clinical Findings

The clinical features observed in this cohort by weighed-HPO terms are listed in Table 2a for 22q13.3 microdeletions. Table 2a also shows the frequencies of clinical features observed in other representative studies with deletion cases (Sarasua et al., 2014a; Tabet et al., 2017, Samogy-Costa et al., 2019). Table 2b shows the frequencies of clinical features observed in patients with *SHANK3* variants, and data from De Rubeis et al. (2018) and other previously published cases (Gauthier et al., 2009; Boccuto et al., 2013; Leblond et al., 2014; O’Roak et al., 2014; Bramswig et al., 2015; Nemirovsky et al., 2015; Zhang et al., 2015; Holder & Quach 2016; Bowling et al., 2017; Lim et al., 2017; Yuen et al., 2017).

**TABLE 2:** Frequency of clinical features observed in this cohort. a) Microdeletions at 22q13.3

HPO clinical features frequencies	This study (189 cases)	Sarasua et al., 2014a (201 cases)	Tabet et al., 2017 (78 cases)	Samogy-Costa et al., 2019 (34 cases)	
≥70 Intellectual disability	95.8% (181/189)	NA	100% (66/66)	NA
≥70 Speech delay	97.4% (184/189)	86.0% (37/43)	100% (65/65)	88.9 (24/27)
≥70 Developmental delay	74.3% (139/187)	88.0% (44/50)	NA	NA
≥70 Hypotonia	75.9% (142/187)	74.5% (82/110)	42.1% (32/76)	84.8% (28/33)
≥70 Behavior abnormalities	79.1%(148/187)	65.3% (83/127)	77.3% (34/44)	NA
≥70 High pain threshold	66.8% (125/187)	77.1% (131/170)	NA	80.0%(24/30)
35–60% ASD diagnosis^a^	62.9% (22/35)	NA	NA	NA
35–60% Pointed chin	58.3% (109/187)	52.3% (58/111)	6.6% (5/76)	NA
35–60% Wide nasal bridge	56.1% (105/187)	NA	2.6% (2/76)	42.3% (11/26)
35–60% Decreased perspiration	52.9% (99/187)	36% (18/50)	NA	NA
35–60% Ear anomalies	45.7% (86/188)	NA	15.8% (12/76)	73.1% (19/26)
35–60% Full brow	39.6% (74/187)	NA	NA	NA
35–60% Impulsive	51.9% (97/187)	40% (78/166)	NA	NA
35–60% Long eyelashes	57.2% (107/187)	84% (95/113)	2.6% (2/76)	11.5% (3/26)
35–60% Bulbous nose	57.8% (108/187)	NA	2.6% (2/76)	15.4% (4/26)
35–60% Large/fleshly hands	54.0% (101/187)	63.4%(71/112)	6.6%(5/76)	NA
40–60% Abnormal emotional response	52.4% (98/187)	NA	NA	NA
35–60% Regressions	47.9% (90/188)	NA	9.2% (6/65)	NA
35–60% Widely spaced teeth/malocclusion	47.1% (88/187)	NA	11.8% (9/76)	7.7% (2/26)
35–60% Hypoplastic/dysplastic nails	40.6% (76/187)	73% (81/111)	3.9% (3/76)	7.7% (2/26)
35–60% Abnormal brain MRI	37.9% (36/95)	NA	NA	NA
40–60% Biting	37.6% (70/186)	45.8% (82/179)	NA	NA
35–60% Excessive yelling	37.1% (69/186)	31% (54/174)	NA	NA
35–60% Uncontrolled laughter	36.9%(69/187)	NA	3.1% (2/65)	NA
20–35% Play frequently with tongue thrusting/sticking out	33.2% (62/187)	NA	NA	NA
20–35% Very sensitive to touch	32.6% (61/187)	NA	NA	NA
20–35% Growth centile >95%	31.9% (60/188)	9.4% (9/96)	4.6% (3/65)	NA
20–35% Seizures	31% (58/187)	54.3% (82/151)	18.5% (12/65)	NA
20–35% Epicanthus	28.7% (54/188)	46.8% (52/111)	10.5% (8/76)	7.7% (2/26)
20–35% 2/3 toe syndactyly	27.3% (51/187)	48.2%(53/110)	10.5% (8/76)	7.7% (2/26)
20–35% Strabismus	26.2% (49/187)	26.6% (29/109)	30.3% (23/76)	11.5% (3/26)
20–35% Macrocephaly	26.1% (49/188)	18.2% (20/110)	1.7% (1/60)	NA
20–35% Sleep disorders	24.1% (45/187)	46.2% (12/26)	5.7% (3/53)	42.4% (14/33)
20–35% Ability to make sentences	25.4% (46/181)	NA	NA	NA
20–35% Deep set eyes	23.5% (44/187)	28.8% (32/111)	NA	NA
20–35% Skin picking	23% (43/187)				
20–35% Hair pulling	22.5% (42/187)	25.5% (48/188)	NA	NA
20–35% Full/puffy cheeks	22.5% (42/187)	NA	NA	NA
20–35% Renal and urogenital anomalies	22.3% (42/188)	26.4% (39/148)	7.5% (4/53)	30.3% (10/33)
20–35% Skin anomalies	22.3% (42/188)	NA	NA	NA
20–35% Ophthalmological anomalies	21.9% (41/187)	NA	NA	NA
20–35% Dolichocephaly	20.2% (38/188)	31.9% (36/113)	NA	NA
<20% Aggressive behavior	19.3% (36/187)	38.6% (49/127)	10.8% (7/65)	NA
<20% Microcephaly	19.7% (37/188)	10.9% (12/110)	6.6% (5/76)	NA
<20% Gastrointestinal problems	18.2% (34/187)	41.6% (62/149)	18.5%(12/65)	56.7%(17/30)
<20% Recurrent infections	16.0% (30/187)	NA	13.2% (7/53)	60.6% (20/33)
<20% Growth centile <3%	12.2% (23/188)	11.5% (11/96)	16.9% (11/65)	NA

a ASD diagnosis according to the psychiatrists of the referring institutions.

**FIGURE 1:**
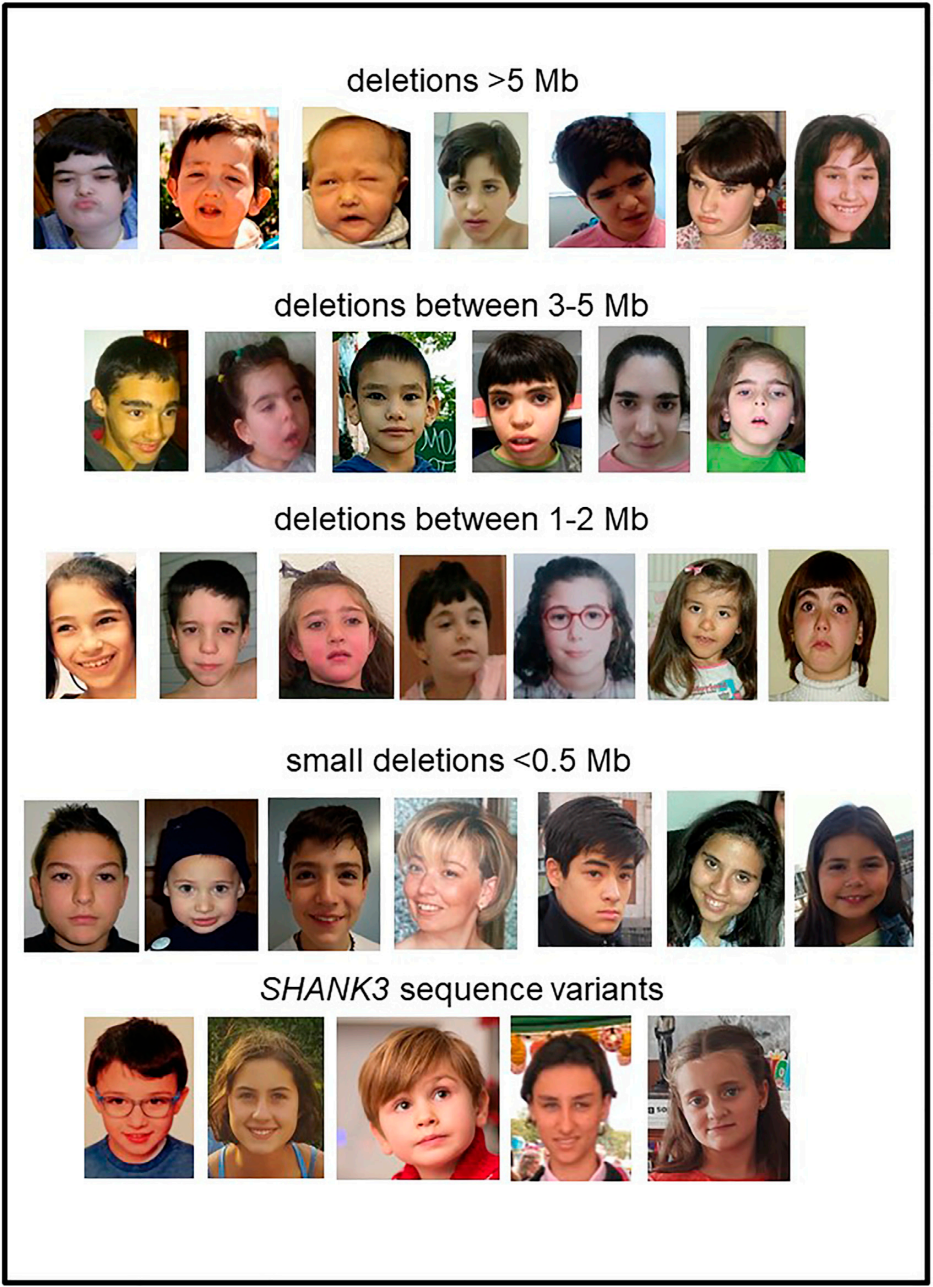
Facial views of individuals with PMS with 22q13.3 deletions or *SHANK3* sequence variants.

Figure 1 shows that facial features are neither typical nor specific for PMS. Patients presented a high degree of facial variability even among individuals with similar deletion size. Significant facial differences can be observed when comparing bigger deletions (>5 Mb) with either small deletions (≤0.5 Mb) or sequence variants in *SHANK3* (Figure 1). Facial features such as bulbous nose, pointed chin, ear anomalies, full eyebrows, long eyelashes, and wide nasal bridge were observed in around 35%–80% of the individuals (Table 2a). These facial features, together with hypotonia, high pain threshold, developmental delay, speech delay, ID, behavior abnormalities, large/fleshly hands, hypoplastic/dysplastic nails, decreased perspiration, and ASD, should be considered as core features of this syndrome (at least in patients with microdeletions; Table 2a). On the other hand, patients with variants in *SHANK3* seemed to have fewer dysmorphic features than patients with microdeletions (Figure 1 and Table 2b).

**FIGURE 2:**
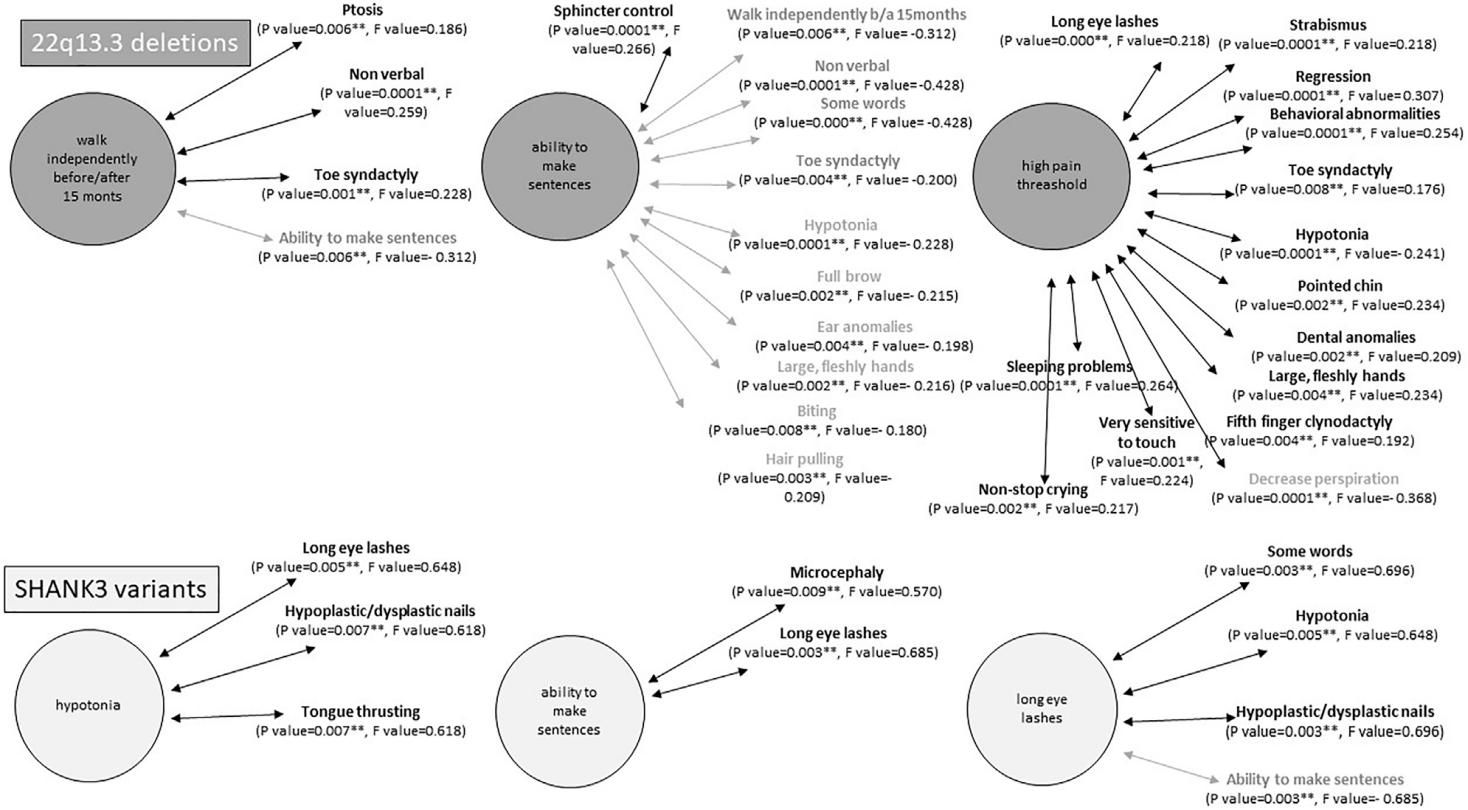
Examples of statistically significant correlations (*p* < 0.001) between intercategorical variables in individuals with 22q13.3 deletions (top) or *SHANK3* variants (bottom). Statistical analyses were performed using Kendal tau_b correlation coefficient. In bold, positive correlations and in gray negative correlations.

Interestingly, many of these core features seem to be inter-related among them. Significant positive correlations were observed when Kendall’s tau_b analysis was performed between categorical variables (Supplementary Table S1). An example with three of these categorical variables is schematized in Figure 2.

Brain MRI studies were performed in 51% (95/187) of individuals in the microdeletion group and 62% (13/21) in the *SHANK3* sequence variant group with abnormal findings found in 38% (36/95) and 15% (2/13), respectively (Table 1a). Abnormal findings included hypoplasia/atrophy of the cerebellar vermis, abnormalities of the corpus callosum (ranging from thinness to agenesis or dysgenesis), abnormalities of the white matter, arachnoid cysts, and hydrocephalus. We also found other abnormalities, such as ventriculomegaly, enlarged cisterna-magna and vermis, prominent metopic suture, cerebral dysplasia with lateral ventricular dilatation, and frontal cerebral hypertrophy.

Speech abilities (evaluated only in patients ≥3 years old; *n* = 199/210, 94.8%) showed severe abnormalities in most of the patients evaluated (148/199, 74.4%). Thirty-five percent of patients (70/199) had no speech at all, around 39% (78/199) had an elementary vocabulary of 10 words or less, and around 26% (51/199) were reported to have a significant vocabulary and the ability to use limited phrases for a short and comprehensible conversation (Table 1a). Table 1 segregates the numbers by deletions and *SHANK3* variants. Remarkably, most of the verbally fluent individuals in the microdeletion group have small deletions.

**FIGURE 3:**
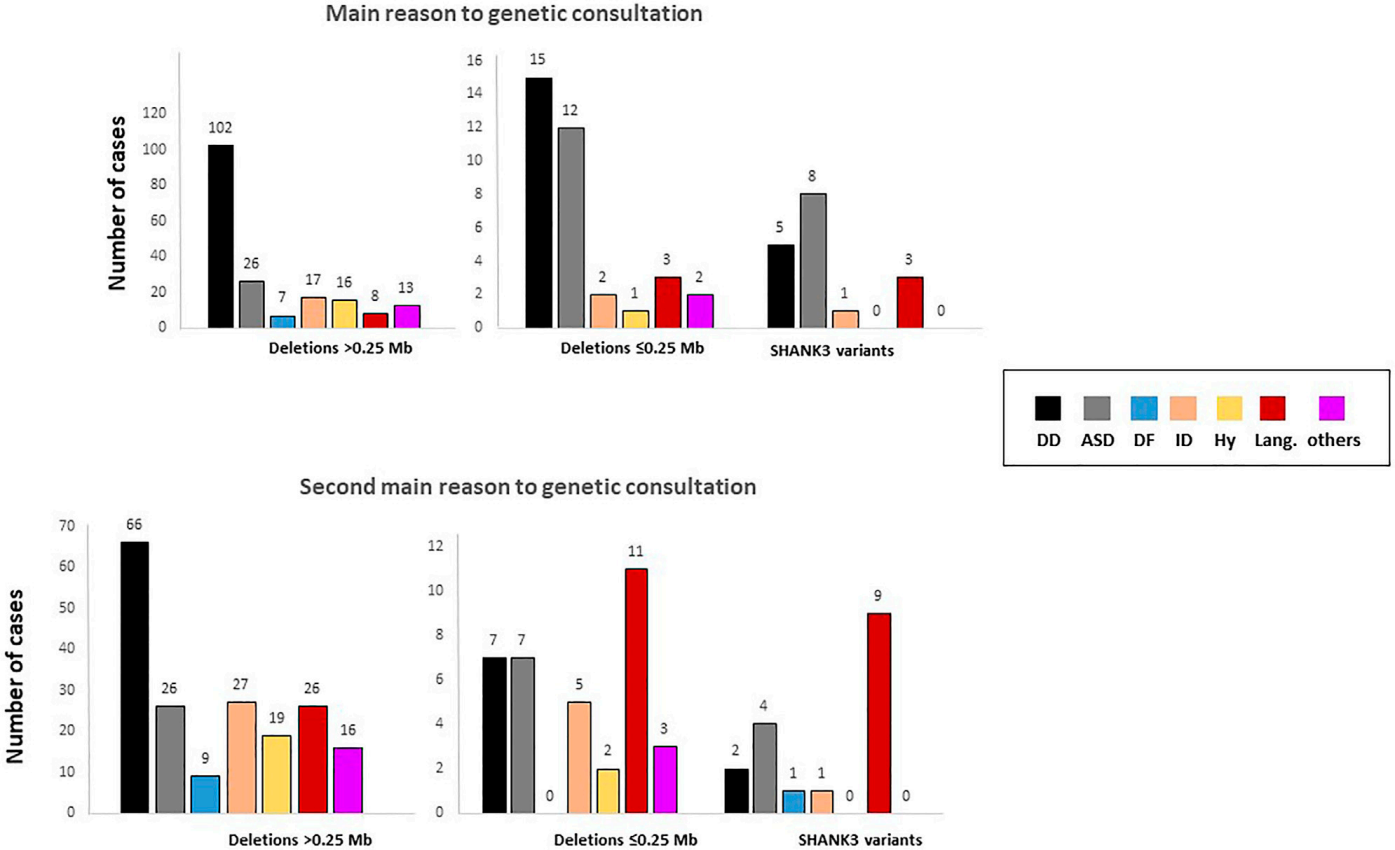
Reasons for referral for genetic evaluation stratified according to the type of genetic defect. Analyses were performed by one-way ANOVA. DD, developmental delay; ASD, autism spectrum disorder; DF, dysmorphic features; ID, intellectual disability; Hy, hypotonia; Lang., language.

The main reason for referral to a genetic consultation in patients with microdeletions was developmental delay, whereas in individuals with sequence variants, ASD and language delay were the most frequent reasons for referral (Figure 3 and Table 1). Similarly, ASD and delayed or absent speech were the main cause of genetic consultation among patients with smaller deletions (≤0.25 Mb). We compared these groups by Chi-square test and z-test (*post hoc*, corrected by Bonferroni). We choose 0.25 Mb as the size of the deletions with the minimal telomeric lost segment, including the *SHANK3* gene. The chi-square test revealed differences between groups constituted by large deletions (>0.25 Mb, 153 cases), small deletions (<0.25 Mb, 36 cases), and *SHANK3* variants (21 cases) for the first- and second-main reasons for referral to genetic consultation (*p* = .0001, F = 43.491 and *p* = .0001, F = 37.491, respectively). These differences were mainly observed between deletions >0.25 Mb and both smaller deletions and variants in *SHANK3* (Figure 3). In addition, hypotonia and dysmorphic features were the main reasons for referral in individuals with medium-size deletions (2.5–5.0 Mb). In patients with deletions ≥5 Mb, the main reason for genetic consultation was severe ID and developmental delay with other severe comorbidities (data not shown).

### Genetic Findings

#### Analysis of 22q13.3 Deletion Breakpoints

**FIGURE 4:**
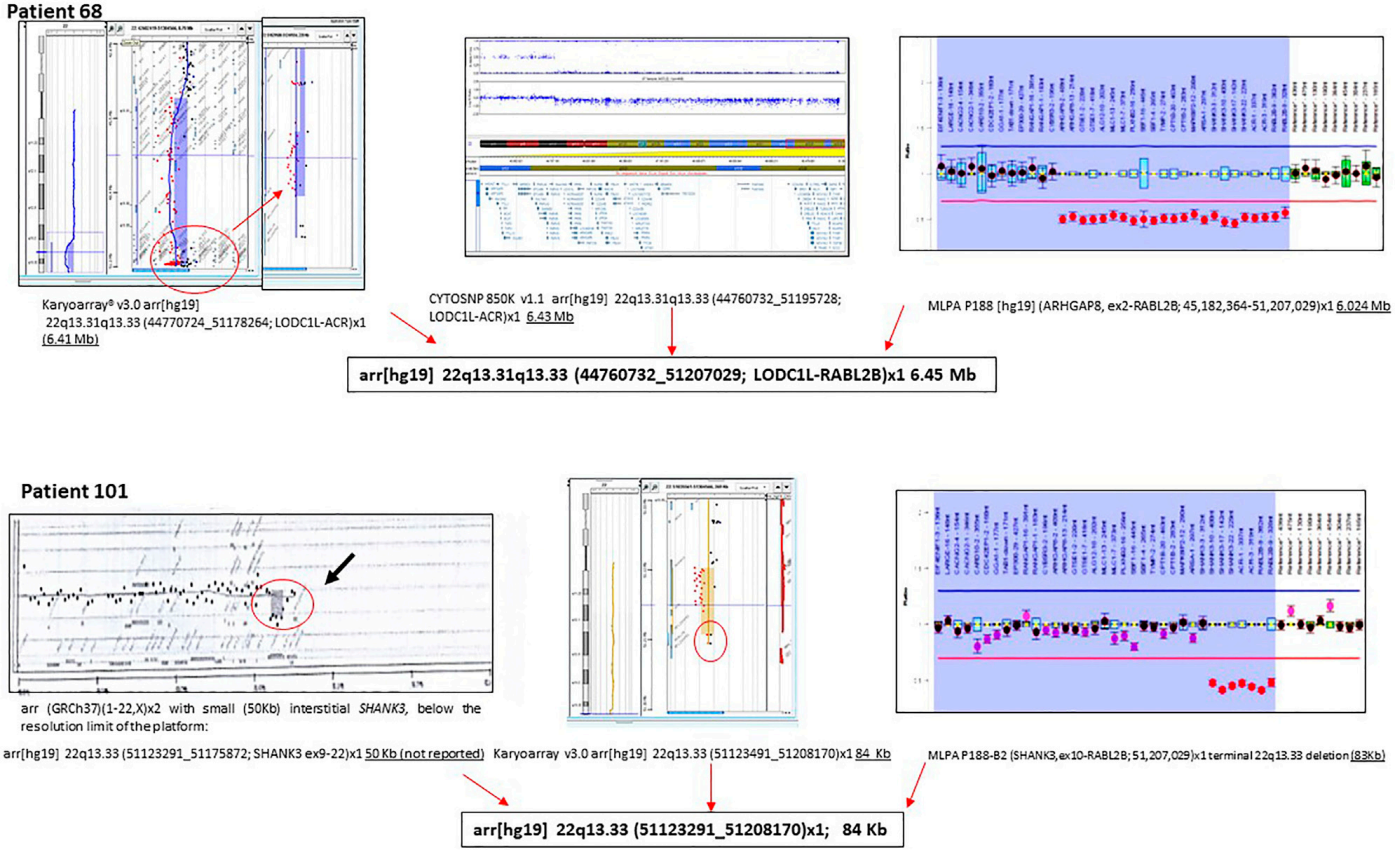
Examples of molecular characterization of two individuals with PMS. Different molecular approaches were used, including CGH-array, SNP array, and MLPA.

We applied different CMA platforms and MLPA approaches to confirm and establish the size of the deletions. Figure 4 illustrates the need to use MLPA for a complete characterization of patients with deletions. This is explained by the lack of probes at the end of the 22q13.33 band in commercial microarrays versus customized microarrays (Supplementary Figure S2). A compilation of additional examples is shown in Supplementary Figure S3.

One-hundred eighty-nine out of 210 individuals carried deletions at 22q13.3 (90%), all of them including *SHANK3* (Table 3). Table 3 also summarizes how the different genomic rearrangements were distributed in the cohort. The number of individuals with ring chromosome 22 (r(22), 20 cases), post-zygotic mosaicism (17 cases), or additional genomic rearrangements (40 cases, including variants of uncertain significance (VUS) and clinically relevant variants in other chromosomes as well as 12 cases with other rearrangements at chromosome 22), is remarkable. Supplementary Table S2 shows the genomic coordinates of the 22q13 deletions and other CNVs identified in the cohort. The mean 22q13 deletion size was 3.52 ± 2.83 Mb (median: 3.29 Mb), ranging from 12 Kb within the last exon of *SHANK3* (individual 51) to 10.30 Mb (individual 170) from the telomere. To our knowledge, the latter is the largest deletion reported so far and was likely not lethal because it is in mosaic form. Cytogenetic data of most of these individuals are shown in Supplementary Table S2.

**TABLE 3:** Summary of genetic findings from the cohort.

Type of genetic alteration	Number of cases
Deletions	189/210 (90%)
Simple terminal deletions	144/189 (76.9%)
Ring 22	20/189 (10.6%)
Mosaic	8/20 (40%)
Unbalanced translocations	13/189 (6.9%)
Inherited	5
De novo	8
Postzygotic mosaic deletions	17/189 (9.0%)
Parental germinal mosaicism	1
Interstitial deletions	12/189 (6.3%)
(including *SHANK3*)	
Additional genomic rearrangements	40/189 (21.1%)
At chromosome 22	12
In other chromosomes	28
*SHANK3* sequence variants	21/210 (10%)
Parental germinal mosaicism	1

**FIGURE 5:**
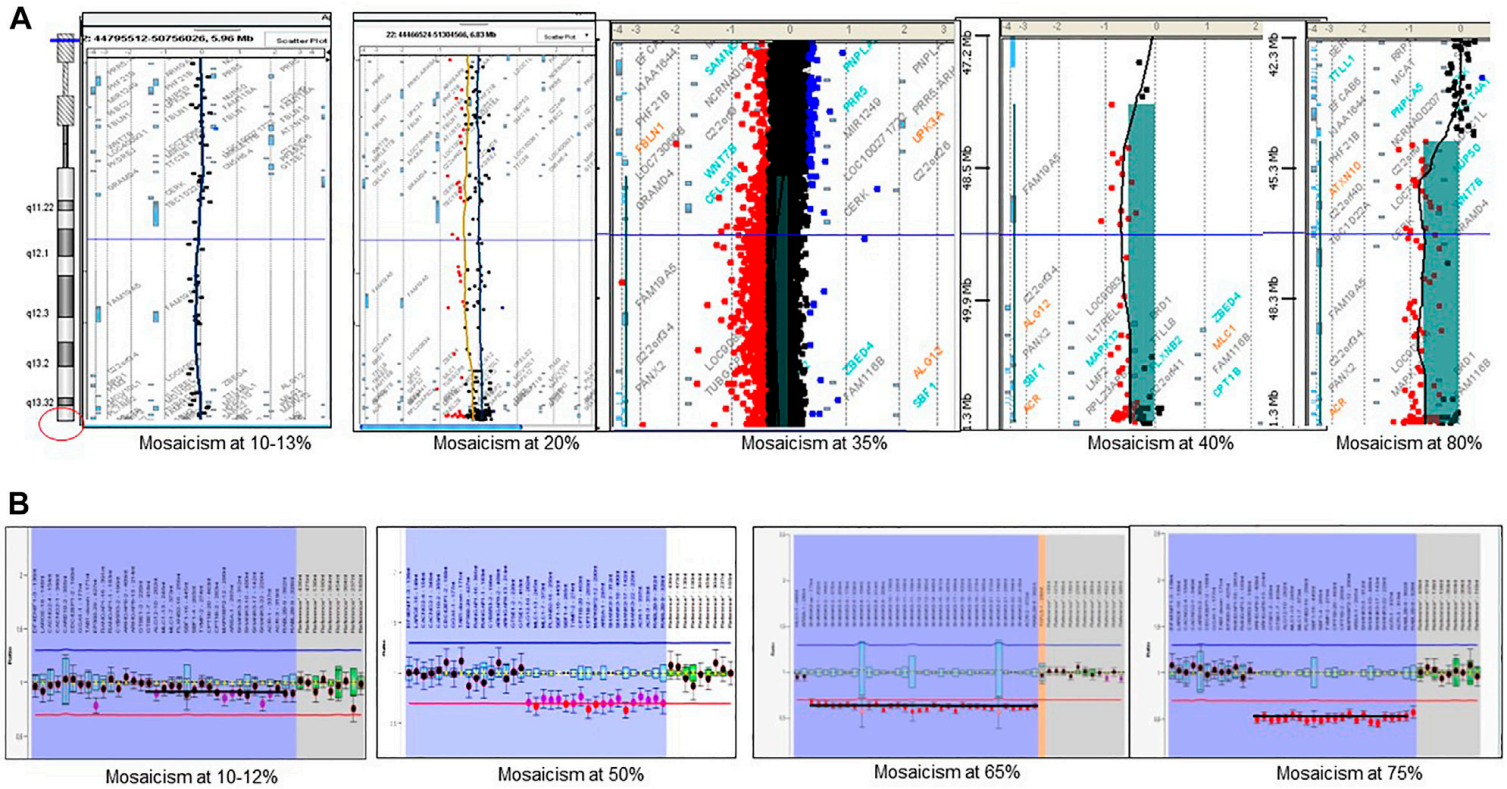
Detection of post-zygotic mosaicism in PMS by using microarrays and MLPA. **(A)** examples of mosaicism detected by CGH-array; **(B)** examples of mosaicism detected by MLPA.

The use of combined SNP arrays and MLPA allowed finding different degrees of post-zygotic mosaicism in microdeletion cases. We found 17 patients with mosaicism ranging from 10% to 82% (Figure 5). In addition, the finding of two siblings with the same deletion (a 48 Kb-interstitial microdeletion with breakpoints within genes *SHANK3* and *RABL2B*, Supplementary Figure S4) suggests parental germinal mosaicism, which was later confirmed as paternal after haplotype analysis using SNP arrays (CytoScan 850K, Illumina).

Breakpoint analyses showed a recurrent 5′breakpoint hot spot, apparently the same described by Bonaglia M. C. et al. (2001). We observed a similar breakpoint in 22 individuals with smaller deletions (coordinates 51123505 to telomere, GCRh37, Supplementary Figure S5). This region is rich in SINEs and LINEs, such as Alu sequences, which could be involved in causing these rearrangements by various mechanisms (Bonaglia et al., 2011; Cooper et al., 2011; Oberman et al., 2015). Our data also point out two additional 3′ recurrent breakpoints (Supplementary Figure S5), which are also extremely rich in Alu sequences. The first recurrent breakpoint was located between coordinates 51146663 and 51175872 (GCRh37; patients 94, 99, and 117) and the second one was located between intron 19 and the end of the last exon of *SHANK3* (NM_001372044.1; patients 31, 57, 75, and 77). Both hypothetical breakpoints were close to the one predicted in a patient reported by Bonaglia M. C. et al. (2001). Additional cases are needed to confirm these new hot spot breakpoints.

### Parental Origin of the Deletions

We tested six highly polymorphic short tandem repeats (STR) to identify the parental origin of the deleted chromosome in 86 trios. In 35 cases (40.7%), the results were noninformative. Among 51 trios with informative findings, we found that deletions originated from the paternally inherited chromosome in 76.5% (39/51) and the maternally inherited chromosome in 23.5% of cases (12/51).

### Sequence Variants in *SHANK3*

In this cohort, we also evaluated 21 patients (10%) carrying *SHANK3* variants (Table 4). All of them were *de novo*; 19 variants were within the penultimate exon (NM_001372044.2), one affected the canonical splicing site at exon 24, and one was located in exon 20. There were 17 frameshift, one nonsense, one splice site, and two missense variants. Some of the variants (Table 4) have been previously described in public databases (ClinVar, LOVD, Varsome) and several publications and are recurrent in our patients (Leblond et al., 2014; Bramswig et al., 2015; Holder & Quach 2016; Thevenon et al., 2016; Yuen et al., 2017; De Rubeis et al., 2018; Zhou et al., 2019; Kaplanis et al., 2020; Feliciano et al., 2019; Lelieveld et al., 2016; Retterer et al., 2016; O´Roak et al., 2014; Farwell et al., 2015; Durand et al., 2007), suggesting several hot spots for *de novo* variants.

**TABLE 4:** *SHANK3* sequence variants identified in this study.

Case	Exon/total exons	Genomic change NC_000022.1(GCRh37/hg19)	Nucleotide change NM_001372044.2	Amino acid change	Effect	ACMG/AMP classification; others
PMS209	20/25	g.51144533dupC	c.2249dupC	p.Leu751ThrfsTer11	frameshift	P (PVS1, PS2, PM2,PP3, PP4)
PMS187^o^	ivs22/ivs24	g.51153476G>A	c.2451+1G>A^a^	?	splice site	P (PVS1, PS2, PM2, PP3,PP5); ClinVar (P, LP)
PMS207	24/25	g.51158717delC	c.2643delC	p.Ala882ArgfsTer73	frameshift	P (PVS1, PS2, PM2, PP4)
PMS124	24/25	g.51159024delG	c.2949delG	p.Pro984ArgfsTer34	frameshift	P (PVS1, PS2, PP4)
PMS213	24/25	g.51159481_51159497delGTGTCTGCCCTGAAGCC	c.3408_3424del	pSer1137GlyfsTer215	frameshift	P (PVS1, PS2, PM2,PP3)
PMS146^o^	24/25	g.51159685_51159686delCT	c.3610_3611delCT^b,c,d,e^	p.Leu1204ValfsTer153	frameshift	P (PVS1, PS2, PM2, PP3, PP5) ClinVar (P, LP)
PMS180^o^	24/25	g.51159685_51159686delCT	c.3610_3611delCT^b,c,d,e^	p.Leu1204ValfsTer153	frameshift	P (PVS1, PS2, PM2, PP3, PP5) ClinVar (P, LP)
PMS208^o^	24/25	g.51159685_51159686delCT	c.3610_3611delCT^b,c,d,e^	p.Leu1204ValfsTer153	frameshift	P (PVS1, PS2, PM2, PP3, PP5) ClinVar (P, LP)
PMS181^m,o^	24/25	g.51159685_51159686delCT	c.3610_3611delCT^b,c,d,e^	p.Leu1204ValfsTer153	frameshift	P (PVS1, PS2, PM2, PP3, PP5) ClinVar (P, LP)
PMS182^m,o^	24/25	g.51159685_51159686delCT	c.3610_3611delCT^b,c,d,e^	p.Leu1204ValfsTer153	frameshift	P (PVS1, PS2, PM2, PP3, PP5) ClinVar (P, LP)
PMS175	24/25	g.51159748C>T	c.3673C>T^n^	p.Pro1225Ser	missense	VUS-LP? (PS2, PM2)
PMS211	24/25	g.51159787delG	c.3712delG	p.Glu1238Argfster19	frameshift	P (PVS1, PS2, PM2, PP3)
PMS185^o^	24/25	g.51159940dupG	c.3865dupG^c,d,f,g,h,i,j,k^	p.Ala1289GlyfsTer69	frameshift	P (PVS1, PS2, PM2,PP3, PP5); ClinVar (P)
PMS212^o^	24/25	g.51159940dupG	c.3865dupG^c,d,f,g,h,i,j,k^	p.Ala1289GlyfsTer69	frameshift	P (PVS1, PS2, PM2,PP3, PP5); ClinVar (P)
PMS165	24/25	g.51160025_51160037del GGGCCCAGCCCCC	c.3950_3962del	p.Arg1317LeufsTer25	frameshift	P (PVS1, PS2, PM2, PP3, PP5); ClinVar (P) CClinPP5)
PMS198^o^	24/25	g.51160025dupG	c.3952dupG	p.Ala1318GlyfsTer40	frameshift	P (PVS1, PS2, PM2, PP3; PP5); ClinVar (P)
PMS214^o^	24/25	g.51160025dupG	c.3952dupG	p.Ala1318GlyfsTer40	frameshift	P (PVS1, PS2, PM2, PP3; PP5); ClinVar (P)
PMS137	24/25	g.51160235dupG	c.4160dupG	p.Ser1391LeufsTer16	frameshift	LP (PVS1, PS2, PM2)
PMS177	24/25	g.51160291_51160312delGAGCCACCCCCTGCCCCTGAGT	c.4216-4237del	p.Glu1406LeufsTer35	frameshift	P (PVS1, PS2, PM2, PP3)
PMS201	24/25	g.51160349G>A	c.4274G>A	p.Arg1425His	missense	VUS-LP (PS2, PM2, PP3)
PMS145^o^	24/25	g.51160594C>T	c.4519C>T^l^	p.Gln1507Ter	nonsense	P (PVS1,PS2, PM2, PP3)

^a^ Bramswig et al. (2015), Holder and Quach (2016), Yuen et al. (2017); ^b^ Leblond et al. (2014); ^c^ De Rubeis et al. (2018); ^d^ Zhou et al. (2019); ^e^ Kaplanis et al. (2020); ^f^ Feliciano et al. (2019); ^g^ Lelieveld et al. (2016); ^h^ Retterer et al. (2016); ^i^ O'Roak et al. (2014); ^j^ Farwell et al. (2015); ^k^ Durand et al. (2007); ^l^ Thevenon et al. (2016); ^m^Individuals PMS181 and PMS182 are siblings; ^n^The variant c.3673C>T(p.Pro1225Ser) has been previously described in two individuals of African descent (gnomAD v2.1.1.), a fact that may question its association with the clinical features observed in the patient; ^o^Variants described previously in unrelated individuals or recurrent in our cohort. P, pathogenic; LP, likely pathogenic; VUS, variant of uncertain significance.

The interpretation of these two missense variants within *SHANK3* remains difficult (Table 4). We classified them as VUS-likely pathogenic by following ACMG/AMP criteria based on *de novo* condition, the individuals’ clinical features, their absence in European non-Finnish population in gnomAD, the domain of the protein affected, *in silico* pathogenicity scores, and its medium-high level of conservation position in the evolution. However, the missense *SHANK3* variant c.3673C>T(p.Pro1225Ser) was observed in two independent individuals of African descent (total allele frequency 7 × 10^−6^; gnomAD v2.1.1), a finding that may question its association with the clinical features observed in the patient.

Finally, the presence of the same *SHANK3* variant in male monochorionic dizygotic twins suggested potential gonadal mosaicism in one of the parents (data not shown). Haplotype analysis using SNP array suggested a paternal origin of the variant. We also have the suspicion for another case with parental mosaicism in a family with two affected twins.

### Genotype-Phenotype Analysis

#### Individual GFAP

**FIGURE 6:**
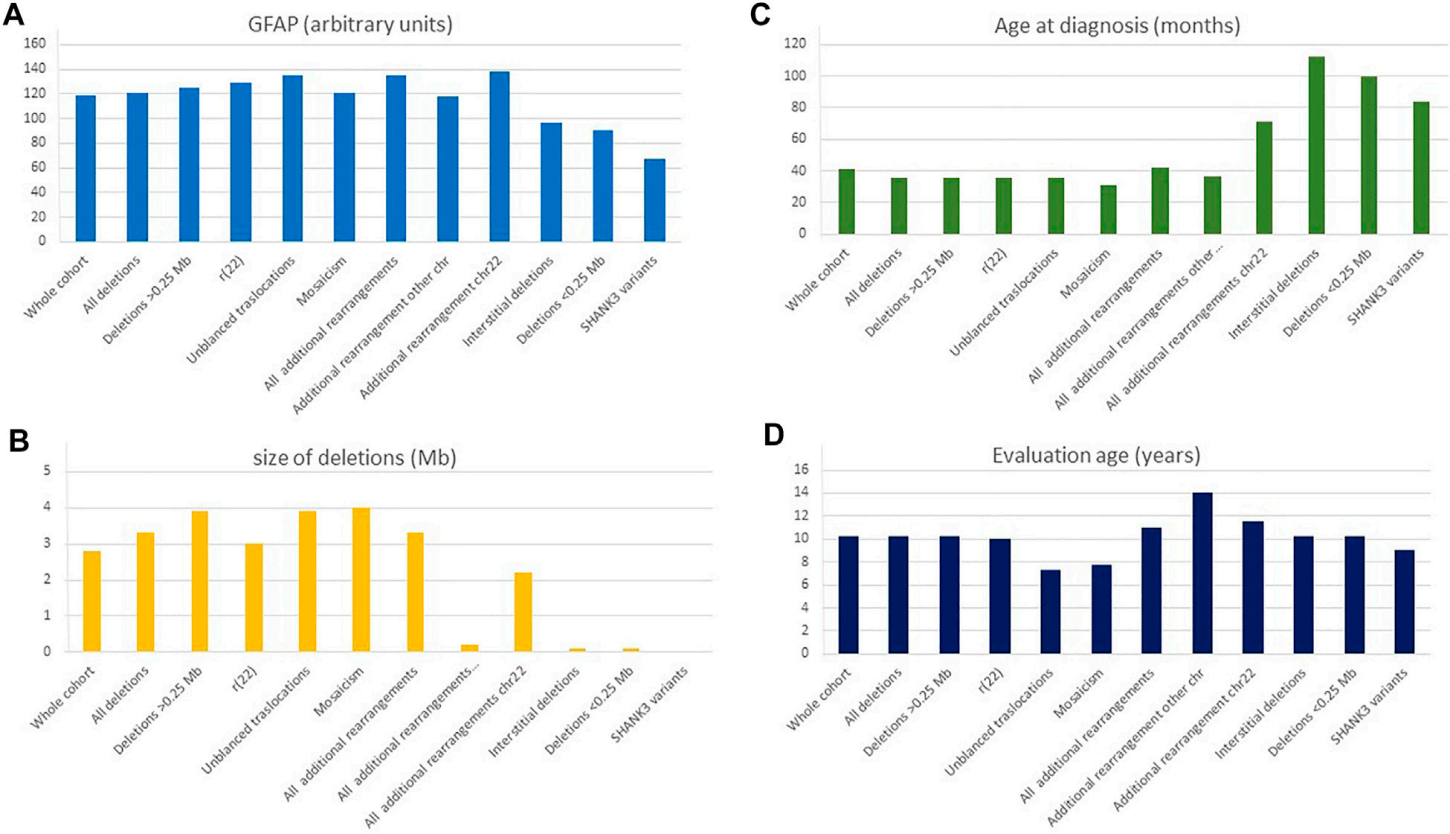
Distribution of continuous variables according to the type of genetic defect. **(A)** GFAP, global function assessment of the patient (arbitrary values); **(B)** Size of the deletions (Mb); **(C)** Age at diagnosis (months); **(D)** Age at evaluation (years). *ASD diagnosis according to the psychiatrists of the referring institutions.

The significant clinical and genetic heterogeneity observed in patients with PMS suggests the type of genetic defect modulates the clinical features. Thus, we propose a numerical score of the GFAP, constructing a continuous variable based on a prioritization array of different “core” clinical weighted-HPO items (*see* Methods). These variables were based on comorbidity items, developmental delay, speech delay, dysmorphic features, and behavior items. Figure 6A shows the median values for GFAP for the whole cohort and different types of genetic defects. Figures 6B–D shows median values for other continuous variables (age at diagnosis and evaluation and size of deletions) in the different groups.

### Comparative Analysis Between Genetic Subgroups

We compared 10 subgroups of individuals with different types of genetic defects: *1*) large deletions (>0.25 Mb; mean size ± SD, 4.29 ± 2.50), *2*) smaller deletions (≤0.25 Mb, 0.10 ± 0.05), *3*) interstitial deletions (1.94 ± 3.55 Mb), *4*) *SHANK3* sequence variants, *5*) ring 22 (3.53 ± 2.44 Mb), *6*) unbalanced translocations (3.69 ± 1.61 Mb), *7*) mosaic deletions (3.5 ± 3.48 Mb), *8*) additional rearrangement at chromosome 22 (3.32 ± 2.02 Mb), *9*) additional rearrangement in other chromosomes (2.62 ± 2.26 Mb), and *10*) all cases with additional rearrangements (2.99 ± 2.26 Mb) (Table 5). Bonferroni or T3-Dunnett *post hoc* tests reveal that the significant differences in the variable “size of deletion” were mainly due to differences between large (>0.25 Mb) and small (≤0.25 Mb) or interstitial deletions, and between small deletions and ring 22 or unbalanced translocations (Table 5).

**TABLE 5:** Comparison between groups with different types of genetic alterations.

Variable	*p*-value/F-value	Statistical test	Pairs of groups with test significant differences after *post hoc* test
Size of deletion^a^	0.0001/40.46	ANOVA	(1.2) (1.3) (2.5) (2.6)
Age at evaluation	0.556/0.59	ANOVA	none
Age at diagnosis	0.008/4.03	ANOVA	(1.2)
GFAP	0.0001/11.24	ANOVA	(1.2) (1.3) (1.4) (1.6) (2.6) (4.5) (4.6) (4.7) (4.8) (4.9) (4.10)
Walk independently before/after 15 months	0.0001/27.51	Chi square	(1.2) (1.4) (2.4) (4.8) (4.9) (4.10)
Single words	0.005/12.89	Chi square	(1.2)
Ability to make sentences	0.0001/27.11	Chi square	(1.2) (1.3) (2.4) (2.6)
Full brow	0.010/11.34	Chi square	(1.4)
Dental anomalies	0.0044/8.09	Chi square	(1.4) (2.4) (4.9) (4.10)
Deep set eyes	0.037/11.84	Chi square	(1.4) (4.6) (4.10)
Toe syndactyly	0.001/17.15	Chi square	(1.2) (1.4)
Large fleshy hands	0.001/17.93	Chi square	(1.2) (1.3)
Sphincter control	0.0001/17.93	Chi square	(1.4) (2.4) (4.8) (4.9) (4.10) (7.8) (7.9)
Very sensitive to touch	0.0001/11.52	Chi square	(1.4) (2.4) (3.4) (4.6) (4.9)
Parental origin	0.021/11.60	Chi square	(8.9)
Recurrent infections	0.016/12.21	Chi square	(1.8)
Hair pulling	0.013/12.74	Chi square	(1.7) (7.9)
Gastrointestinal anomalies	0.002/17.22	Chi square	(1.7) (1.8) (1.10)
Sleeping problems	0.020/11.69	Chi square	(1.4) (1.10) (4.10)
Epicanthus	0.046^FET^/5.14	Chi square	(1.4)
Full/puffy eyelids	0.026/7.82	Chi square	(1.4)
Poor visual contact	0.043^FET^/4.22	Chi square	(1.4)
Formal ASD evaluation	0.040^FET^/4.22	Chi square	(1.4)
Abnormal emotional response	0.047^FET^/3.67	Chi square	(1.4)
Growth, centile >95th	0.054^FET^/3.29	Chi square	(1.4)
Hypotonia	0.059^FET^/3.55	Chi square	(1.4)

Group 1 (deletions >0.25 Mb, mean size ± SD, 4.29 ± 2.50); group 2 (smaller deletions ≤0.25 Mb, 0.10 ± 0.05); group 3 (interstitial deletions, 1.94 ± 3.55 Mb); group 4 (*SHANK3* variants); group 5 (ring 22, 3.53 ± 2.44 Mb); group 6 (unbalanced translocations, 3.69 ± 1.61 Mb); group 7 (mosaic deletions, 3.5 ± 3.48 Mb); group 8 (additional rearrangement at chromosome 22, 3.32 ± 2.02 Mb); group 9 (additional rearrangement in other chromosomes, 2.62 ± 2.26 Mb), and group 10 (all cases with additional rearrangements, 2.99 ± 2.26 Mb). FET, corrected by Fisher’s exact test; GFAP, global functional assessment of the patient.

a Group 4 (*SHANK3* variants) was not included in the analysis of deletion size.

Using the GFAP, we observed significant differences mainly between patients with large deletions compared with patients with small deletions, interstitial deletions, and sequence variants (Table 5). Remarkably, no significant differences were detected between small deletions and individuals with sequence variants in *SHANK3* (Table 5).

Pearson statistical analysis was performed to explore correlations between these continuous variables. We observed significant direct correlations between size of the deletion and GFAP (Pearson value = 0.33, *p* = .0001) as well as inverse correlations between age at diagnosis and size of the deletions (Pearson value = −0.240, *p* = .001) and GFAP (Pearson value = −0.133, *p* = .03). Altogether, our data suggest that the age at diagnosis seems to be inversely related to the degree of difficulty at diagnosis. Indeed, patients with small deletions (below 0.25 Mb; mean 0.10 ± 0.05 Mb) were diagnosed later (mean 7.61 ± 4.47 years) than those with large-size deletions (˃0.25 Mb, 4.35 ± 2.62 Mb, mean age at diagnosis: 5.52 ± 7.87 years). This fact was also observed in patients with interstitial deletions (mean age at diagnosis 9.75 ± 8.07 years and 1.91 ± 3.51 Mb for deletion size) and *SHANK3* gene variants (mean age at diagnosis 7.86 ± 4.49 years).

Individuals with r(22), mosaic deletions, and unbalanced translocations affecting the 22q13 band were diagnosed significantly earlier than the average (mean ages 5.59, 4.41, and 3.57 years, respectively) even though the mean deletion size in those cases was 3–4 Mb (3.19, 3.24, and 3.91 Mb, respectively) similar to the average of the cohort (median 3.08 Mb).

Although individuals with small deletions and *SHANK3* variants showed similar findings in most of the categorical variables (Table 5), a remarkable difference was observed in “the ability to make sentences” between the two groups, with 30/65 (46.2%, Supplementary Table S3) of individuals with deletions below 0.25 Mb able to make sentences compared with 5/18 (27.7%, Table 1a) among those with *SHANK3* variants. Interestingly, we also found significant differences in the variable “parental origin” between groups with additional rearrangements (at chromosome 22 vs. other chromosomes). As expected, significant differences were found between all deletions and individuals with *SHANK3* variants, mostly affecting dysmorphic features (Table 5).

No statistically significant differences were detected between gender and continuous variables (size of the deletion, age of diagnosis, age of evaluation or GFAP, Student’s *t*-test, data not shown). However, significant differences were observed between gender and several categorical variables (seizures, decreased perspiration, microcephaly, fifth finger clinodactyly, and lymphedema; chi-square test, *p* = 0.023, 0.056, 0.008, 0.029 and 0.001, respectively; data not shown), with higher frequencies in females.

Finally, we observed significant differences between parental origin and GFAP (*p* = 0.048, Student’s *t*-test) and two categorical variables, high pain threshold and lymphedema (chi-square test, *p* = 0.039 and 0.027, respectively, *n* = 51). In all cases, maternal origin (*n* = 12) was associated with higher GFAP values and with a worse prognosis (Table 5).

### Genotype-Phenotype Correlations

We applied Ward’s hierarchical cluster analysis using deletion size as the unique variable to test how individuals with microdeletions group according to their deletion size. Individuals were grouped into four clusters (the number was established by BIC and AIC algorithms) as follows: cluster 1: 0.52 ± 0.51 Mb (64 individuals), cluster 2: 3.39 ± 0.77 Mb (66 individuals), cluster 3: 6.10 ± 0.69 Mb (29 individuals), and cluster 4: 8.27 ± 0.74 Mb (28 individuals). Extended variable frequencies in each cluster are shown in Supplementary Table S3. One-way ANOVA followed by a *post hoc* test (Bonferroni or T3-Dunnett) revealed statistically significant differences between age at diagnosis, GFAP, and size of deletions in different clusters (*p* = 0.009, 0.0001, and 0.0001, respectively, Table 6). Supplementary Figure S6 shows that some clinical findings, such as “ability to make sentences” or “walk independently before/after 15 months,” were preferentially associated with cluster 1. In fact, in cluster 1 (deletions 0.52 ± 0.51 Mb), 53.8% of these individuals were able to make sentences (35/65), followed by 15.6% (10/64) in cluster 2 and only 3.7% (1/27) in clusters 3 and 4. The chi-square test followed by z *post hoc* test with Bonferroni correction showed significant differences among clusters for several categorical variables (Table 6).

**TABLE 6:** Comparison between Ward’s clusters obtained using deletion size.

Variable	*p*-value/F-value	Statistical test	Pairs of clusters with significant differences after *post hoc* test
Size of deletion	0.001/7.509	ANOVA	(1.2) (1.3) (1.4) (2.3) (2.4)
Age at diagnosis	0.009/3.861	ANOVA	(1.2)
GFAP	0.001/7.509	ANOVA	(1.2) (1.3) (2.3) (2.4)
Age at evaluation	0.086/1.951	ANOVA	none
Walk independently before/after 15 months	0.0001/18.996	Chi square	(1.2) (1.4)
Growth, percentile >95th	0.020/9.867	Chi square	(2.4)
Ability to make sentences	0.0001/27.996	Chi square	(1.2) (1.3) (1.4)
Some words	0.0001/17.906	Chi square	(1.2) (1.3)
Hypotonia	0.003/13.726	Chi square	(1.3)
Microcephaly	0.012/10.897	Chi square	(1.3)
Macrocephaly	0.004/13.512	Chi square	(1.4) (2.4)
Sphincter control	0.009/11.604	Chi square	(1.3)
Renal and urogenital anomalies	0.009/11.504	Chi square	(1.4)
Lymphedema	0.0001/26.883	Chi square	(1.4) (2.4)
Ear anomalies	0.009/11.504	Chi square	(1.4)
Biting	0.037/8.494	Chi square	(1.2)
Nonstop crying	0.044/8.116	Chi square	(3.4)

Mean deletion size cluster 1 (0.52 ± 0.51 Mb), cluster 2 (3.39 ± 0.77 Mb), cluster 3 (6.10 ± 0.69 Mb), and cluster 4 (8.27 ± 0.74 Mb). GFAP, global functional assessment of the patients.

When Ward’s clusters were dissected by frequencies of these variables (in percentages), we observed higher frequencies of several core features, considered as a better prognosis, in cluster 1 than in other clusters (Supplementary Table S3). On the other hand, higher percentages of other core items, reflecting comorbidity (normally associated with a worse prognosis; renal and urogenital abnormalities, hearing problems, lymphedema, no words, or growth above the 95th percentile, Supplementary Table S3) mapped preferentially in cluster 4, which is associated with large deletions. Finally, other items seemed to correlate directly (toe syndactyly, ear anomalies, GFAP, MRI anomalies, abnormal emotional response*,* or renal and urogenital anomalies) or inversely (age at diagnosis) to the size of the deletions (Supplementary Table S3).

Linear regression was used to obtain a coefficient of correlation to deletion size at 22q13 for each feature (Table 7). The coefficient of correlation ranged between 0 and 0.7. “F value” was examined to determine if the coefficient of correlation was significant. For most features, no correlation to deletion size was found. However, several clinical features were found to have a statistically significant correlation with the size of the deletion (Table 7), including the ability to make sentences, lymphedema, macrocephaly, renal and urogenital anomalies, and brain MRI anomalies. At a significance level of 0.05, one would expect 1 in 20 significant correlations by chance, whereas 14/61 (23%) correlations for the size of deletion were obtained. With a similar approach, we identified 6/61 (9.8%) correlations with age at diagnosis and 8/61 (13.1%) with age at evaluation.

**TABLE 7:** Comparison of clinical features and the size of the 22q13 deletion, age at diagnosis and age at evaluation using linear regression to obtain a coefficient of correlation.

Clinical feature	Coefficient of correlation	Significance F
**Dependent variable: size of deletion**		
Ability to make sentences	0.37	0.0001
Lymphedema	0.49	0.0001
Macrocephaly	0.53	0.002
Renal and urogenital anomalies	0.55	0.010
Seizures	0.57	0.014
Other genomic rearrangements	0.59	0.021
Sphincter control	0.61	0.011
Abnormal brain MRI	0.63	0.013
Deep set eyes	0.65	0.011
Growth, percentile >95th	0.66	0.037
Herniae	0.67	0.024
Abnormal emotional response	0.68	0.037
Toe syndactyly	0.69	0.036
Epicanthal folds	0.70	0.046
**Dependent variable: age at diagnosis**		
Sphincter control	0.23	0.003
Biting	0.29	0.017
Seizures	0.33	0.024
Dolichocephaly	0.37	0.020
Lip/palate anomalies	0.41	0.026
Nonverbal	0.43	0.046
**Dependent variable: age at evaluation**		
Brain MRI	0.27	0.0001
Sphincter control	0.35	0.002
ASD diagnosis^a^	0.39	0.015
Dolichocephaly	0.43	0.012
Ability to make sentences	0.46	0.010
Seizures	0.50	0.004
Obesity	0.52	0.025
Poor visual contact	0.54	0.029

a ASD diagnosis according to the psychiatrists of the referring institutions.

## Discussion

We describe one of the largest series of patients with PMS characterized by CMA and other genetic approaches, including karyotype, MLPA, and FISH. We also explored the high genetic, and phenotypic variability observed in PMS individuals. Although the true prevalence of this rare disease is still unknown, it is among the most common subtelomeric microdeletion syndromes (Delahaye et al., 2009). Previous findings show that PMS is diagnosed in around 0.5% of individuals with ASD and ID (Cooper et al., 2011; Betancur and Buxbaum, 2013; Leblond et al., 2014; Chen et al., 2017; Samogy-Costa et al., 2019). Previous data suggest that the prevalence of this syndrome remains underestimated worldwide due to several reasons:

a) The lack of a distinctive phenotype without significant dysmorphic features (Figure 1). In most cases, individuals carrying *SHANK3* variants and small deletions do not have a distinctive facial appearance.
b) High genetic and clinical variability. We observed marked intracohort variability. Analysis of GFAP revealed significant differences depending on the type of genetic defect and the type of rearrangements found in individuals. We found additional rearrangements in 21.2% of the cases. Some of them involved other OMIM-related syndromes (Supplementary Table S2), including hereditary neuropathy with liability to pressure palsies (OMIM#162500), affecting *PMP22*; Chromosome 15q11.2 deletion syndrome BP1-BP2 (OMIM#615656), affecting *NIPA1-NIPA2*; 15q13.3 deletion syndrome (OMIM#2612001), affecting *CHRNA7*, and 16p11.2 microdeletion syndrome (OMIM#611913), which may contribute partially to the variability of some individuals. Previous studies also report the presence of additional rearrangements with putative clinical relevance in individuals with PMS (Tabet et al., 2017; Samogy-Costa et al., 2019). Interestingly, our data show that individuals with additional rearrangements and, in particular, those with small 22q13 deletions had higher values of GFAP (associated with worse prognosis) than cases with simple small deletions. In our series, some of the patients carried the same additional CNVs reported by Tabet and others (2017), in most cases inherited from a reportedly healthy parent. We do not know the consequences of these findings or if it is just a coincidence. Most of these and other similar CNVs (15q11.2 deletions and duplications, 15q13.3 deletions and duplications, 16p13.11 deletions, 16p12.1 deletions, 16p11.2 proximal and distal deletions, 17q12 deletions and duplications, and 22q11.21 duplications) are linked to susceptibility loci for a variety of pediatric diseases (Girirajan and Eichler, 2010; Cooper et al., 2011). For some of these CNVs, the enrichment in affected individuals (mainly ID, ASD, or DD cases) in comparison with healthy controls seems to give them a putative pathogenic classification (Rosenfeld et al., 2013).
c) The difficulty in detecting chromosome 22 microdeletions in routine cytogenetic analysis even at the 550–850 band level of resolution. Our data show that small terminal deletions, interstitial deletions, and *SHANK3* variants were diagnosed later than those carrying other type of rearrangements, such as ring chromosomes, mosaic deletions, or unbalanced translocations. Thus, most cases were diagnosed in tertiary hospitals that applied CMA testing as a first-tier test through its laboratory routines for individuals with ID, ASD, and congenital malformations, following international guidelines (Miller et al., 2010). Misdiagnosis or underdiagnosis of mosaicism could be observed when using CMA as a unique tool. Mosaicism lower than 15% cannot be easily detected by CMA (Figure 3) owing to the variability of the assay and the fact that most of the commercial CMA platforms do not have a significant number of probes at the end of the telomere of chromosome 22 (Supplementary Figure S5). FISH or MLPA combined with CMA must be applied in suspected patients. We found an unexpectedly high number of post-zygotic mosaicism (17/189; 9.0%) in patients with microdeletions when compared with a previous report, which established a mosaic frequency of around 2.5%–5.8% for deletions at 22q13.3 (Samogy-Costa et al., 2019). It is not easy to predict the expected clinical features in patients with mosaicism though patients with <10% of mosaicism in blood can present a complete manifestation of the disease (Phelan et al., 2018). We also found two independent families with suspected gonadal mosaicism. This aspect is important because it complicates genetic counseling. Germinal mosaicism in PMS is not frequent, but it has been described in a few families (Tabolacci et al., 2005; Durand et al., 2007; Gauthier et al., 2009; Nemirovsky et al., 2015; Zwanenburg et al., 2016).

In PMS individuals with terminal deletions diagnosed with CMA, it is essential to rule out the presence of r(22). Confirmation of r(22) has significant implications for clinical management because individuals with r(22) have an increased risk of tumors in the nervous system due to biallelic loss of the *NF2* (neurofibromatosis type 2) gene (Lyons-Warren et al., 2017; Ziats et al., 2020). We observed three out of 20 patients with r(22) with neurofibromatosis type 2; these three individuals were included in the series reported by Zyats and others (2020). The prevalence of tumors associated with r(22) is unknown. Thus, we recommend follow-up of PMS patients carrying r(22) and highlight the importance of karyotyping individuals with terminal deletions of the long arm of chromosome 22.

d) Difficulties in testing *SHANK3* variants. Implementing exome or panels to analyze *SHANK3* variants was rare and expensive during the period of recruitment of this cohort in our country. However, in recent years (2019–2020), we recruited 18 patients with *SHANK3* variants.

**FIGURE 7:**
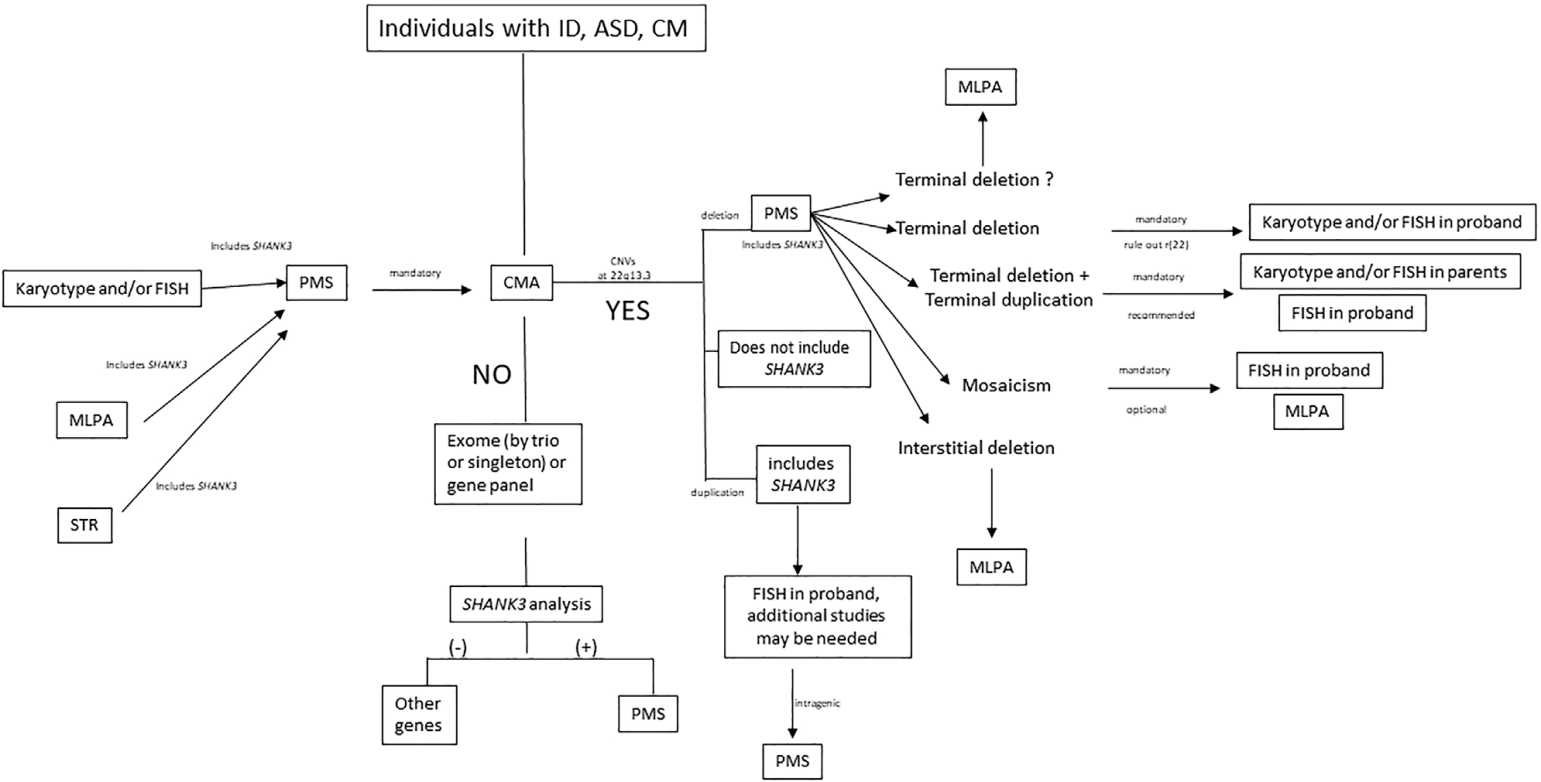
Laboratory algorithm for management of samples suspected of PMS. ID, intellectual disability; ASD, autism spectrum disorder; CM, congenital malformations; PMS, Phelan-McDermid syndrome; CMA, chromosome microarray analysis; MLPA, multiplex ligation-dependent probe amplification; STR, short tandem repeat; CNV, copy number variation; r(22), ring chromosome 22.

We propose an algorithm for laboratory management of individuals with PMS (Figure 7). We recommend CMA as a first-tier test for patients with ID and ASD to determine the exact deletion size, define the deletion breakpoints, and detect additional genomic rearrangements, such as terminal duplications in other chromosomes. Most patients also need other molecular approaches, such as MLPA or FISH, for accurate laboratory characterization (Supplementary Figure S5). Terminal deletions need karyotyping to rule out a r(22), and FISH is mandatory in parents when suspicion of unbalanced or balanced translocation is suspected. Low-grade mosaicism may be detected by applying FISH in the proband. When other techniques, such as FISH or MLPA, established the diagnosis of PMS as the first test (Figure 7), CMA is still mandatory to complete the diagnosis of individuals (to determine the affected genes, deletion size, other rearrangements, etc.). Finally, when all cytogenetic and molecular approaches are negative in individuals with ID or ASD with other clinical features of PMS, we recommend an exome-analysis (trio or singleton) with extensive analysis of *SHANK3* sequence variants (Figure 7).

It is also remarkable that, although formal ASD studies were only performed in 20% (43/210) of the cohort, 29/43 (67%) of them have an ASD diagnosis according to the psychiatrists of the referring institutions. Thus, for PMS individuals, formal ASD evaluation is mandatory. Sixty individuals of this cohort are included in a recent study of the behavioral profile in PMS performed by our colleagues (Burdeus-Olavarrieta et al., 2021).

### Genotype-Phenotype Correlations

It is suggested that the haploinsufficiency of *SHANK3* is the most significant contributor to PMS. We believe that *SHANK3* is a major contributor to the neurocognitive features of the syndrome, but not the only one. Other genes may contribute to the PMS phenotype by modulating *SHANK3* action. Several authors review a possible effect of different genes in the PMS phenotype (Tabet et al., 2017; Mitz et al., 2018; Ziats et al., 2019; Li et al., 2020; Ricciardello et al., 2021), but how those genes contribute is still unknown.

Only a few studies investigate putative relations between the size of the deletions and clinical features of PMS, and the causality remains unclear (Cusmano-Ozog et al., 2007; Dhar et al., 2010; Sarasua et al., 2011; Soorya et al., 2013; Sarasua et al., 2014a; Sarasua et al., 2014b; Tabet et al., 2017; Samogy-Costa et al., 2019). The clinical features of patients with pathogenic variants in *SHANK3* overlap with those of individuals with deletions, giving this gene an important role in the spectrum of clinical features of PMS.

We found that speech skills, one of the main features of the syndrome, might be directly associated with the size and/or mapping of the deletion. Indeed, most individuals who can make sentences (aged older than 3 years) had smaller deletions, supporting previously described observations (Sarasua et al., 2014a; Samogy-Costa et al., 2019; Brignell et al., 2021). In addition, among individuals with *SHANK3* variants, 27% (5/18) of patients in this study were able to maintain short conversations, compared with 18% (3/17) and 38% (3/8) of individuals verbally fluent reported by De Rubeis et al. (2018) and Xu et al. (2020), respectively.

Our data also support significant differences between individuals with *SHANK3* variants and small deletions in the ability to make sentences. Thus, other genes or some interaction nearby could modulate language abilities. In fact, a recent study also showed that *SHANK3* seemed necessary but not exclusive for expressive language in PMS individuals (Brignell et al., 2021).

Additional differences between individuals with *SHANK3* variants and those with small deletions were also observed for cognitive features, such as sleeping anomalies or sphincter control, with higher frequencies in individuals with *SHANK3* variants than in the smaller deletion group. As expected, differences in several facial dysmorphic features were observed between individuals with deletions and *SHANK3* variants.

The cluster analysis showed a positive correlation between deletion size and GFAP, brain MRI abnormalities, ear anomalies, and toe syndactyly as well as a negative correlation between deletion size and age at diagnosis and abnormal emotional response. It is also clear that several clinical features mapped preferentially in specific regions of the clusters. Indeed, two clear genomic regions can be associated with the size of the cranium. Whereas medium- and large-size deletions seem to be associated with macrocephaly, microcephaly seems to be present only in patients with small deletions. We established an interval between 0.40 and 3.4 Mb linked to microcephaly and between 4.50 and 8 Mb from the telomere related to macrocephaly. This fact suggests the contribution of at least two independent genes for alterations in the cranium size. Interestingly, there are no more than 10 high dosage-sensitive genes (ClinGen, http://www.clinicalgenome.org) in the latter interval. Among them is *GRAMD4*, which has been established experimentally to have protein-protein interaction with PIAS1 (Supplementary Figure S7). PIAS1 is a member of the ubiquitin protein family, like PIAS4. The *PIAS4* gene has been involved in macro/microcephaly in distal 19p13.3 microdeletion/microduplication syndrome (Nevado et al., 2015; Tenorio et al., 2020).

The existence of interstitial deletions not including *SHANK3* (Wilson et al., 2008; Disciglio et al., 2014; Ha et al., 2017; this study), which partly overlap some clinical features of PMS (Supplementary Table S4), may also indirectly support a role for additional genes in the clinical spectrum of PMS. At this point, we cannot rule out a positional/regulating effect on *SHANK3* in all these cases, nor global alteration of topological chromatin organization (TAD; topological association domains) as is been suggested by others (Kurtas et al., 2018; Srikanth et al., 2021) rather than simply by the deletion of dosage-sensitive genes. This hypothesis needs to be explored in future studies.

### Correlations by Age

A previous large cohort study reported a small but significant increase with age of several clinical findings in PMS, including sensory dysfunction, reduced response to pain, epilepsy, and lymphedema (Sarasua et al., 2014b). Similarly, the risk of psychiatric disorders in PMS increases with age (Denayer et al., 2012; Verhoeven et al., 2012; Kolevzon et al., 2019). Regarding the correlation of clinical features with age, our data cannot support any solid conclusion about the contribution of age to the clinical features of PMS. We found in our cohort six and eight items of 61 that rejected this null hypothesis (∼10% and 13%) for age at diagnosis and age at evaluation, respectively. This is twice the number expected by chance.

## Conclusions

Here, we report a large series of Spanish and South American patients with PMS, focusing on phenotype-genotype correlations. The analysis of individuals with sequence variants and their comparison with patients with small deletions support the notion that *SHANK3* is essential in most core phenotypic findings of PMS but is not the unique one. Additional genes may modulate the whole phenotype in PMS individuals with microdeletions.

The existence of different types of rearrangements and genomic variations may explain the high variability observed in PMS individuals. Finally, an accurate laboratory approach for PMS individuals using a diagnostic algorithm is proposed to offer appropriate management, follow-up, and genetic counselling to these families.

## Spanish PMS Working Group

INGEMM, Madrid, Spain (Rocío Mena, Roser Lleguer, Victoria Fernández-Montaño, Rubén Martín, Blanca Fernández, Fé García-Santiago, Victoria Gómez del Pozo, Carolina Peña; Spanish PMS Association (Norma Alhambra, Carlos García, Juan Ramón Rodríguez); Servicio de Neuropediatría, Hospital Universitario La Paz, Madrid, Spain (Antonio Martínez-Bermejo); Hospital Central de Asturias, Oviedo, Spain (Ignacio Málaga); Hospital San Joan de Deu, Barcelona, Spain (Antonio Federico Martínez-Monseny, Judith Armstrong, Jennifer Anticona, Cristina Hernando-Davalillo, Adrián Alcalá San Martí, Loreto Martorell, Delia Yubero, Tania Nunes, Mar O´Callaghan, Carlos Ortez, Xenia Alonso, Federico Ramos, Jesús Casas López); Hospital Virgen de la Arrixaca, Murcia, Spain (Vanesa López-González, M. Juliana Ballesta); Q- Genomics Laboratory Barcelona, Spain (Lluís Armengol); Hospital Virgen del Rocío, Sevilla, Spain (Antonio González-Meneses; Salud Borrego); Hospital Universitario la Fé, Valencia, Spain (Mónica Roselló); NIM-Genetics, Madrid, Spain (Javier Suela); Hospital Son Espases, Palma de Mallorca, Spain (Ángeles Pérez-Granero); Hospital Clinic, Barcelona, Spain (Laia Rodríguez-Revenga).

## Data Availability

The datasets presented in this study can be found in online repositories. The names of the repository/repositories and accession number(s) can be found below: DECIPHER Genomics, accession no: 432868 - 433079, IMMGPMS1 - IMMGPMS211.
