# Supplementary material for: Variability in Phelan-McDermid Syndrome in a Cohort of 210 Individuals
Source: Front Genet. 2022 Apr 12;13:652454. doi: 10.3389/fgene.2022.652454 (PMC9044489; doi:10.3389/fgene.2022.652454)
Supplement: Supplementary file 2 [file Presentation1.PPTX]

## Slide 1
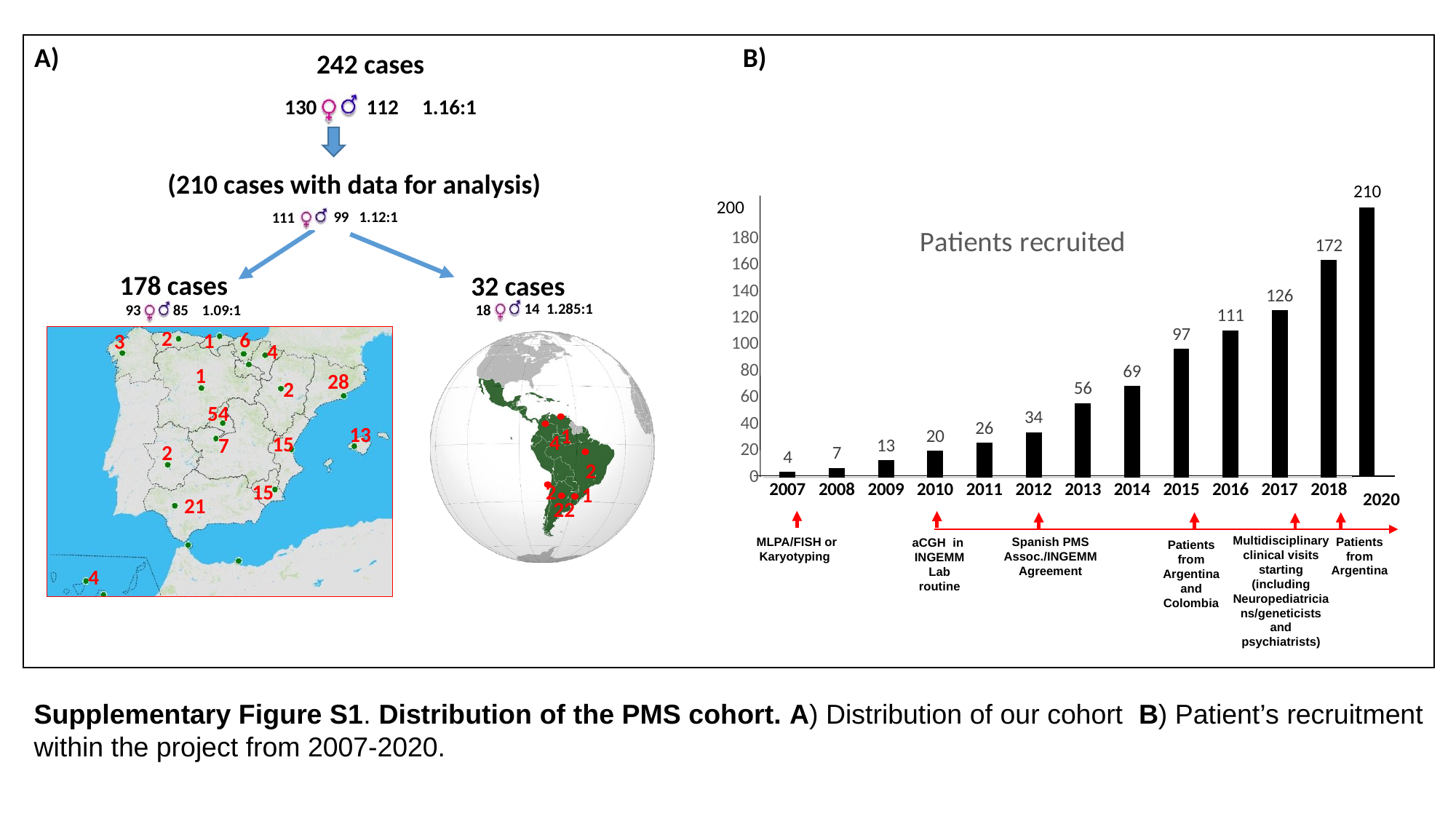

A)
B)
210
200
### Chart: Patients recruited
| Category | |
|---|---|
| 2007 | 4.0 |
| 2008 | 7.0 |
| 2009 | 13.0 |
| 2010 | 20.0 |
| 2011 | 26.0 |
| 2012 | 34.0 |
| 2013 | 56.0 |
| 2014 | 69.0 |
| 2015 | 97.0 |
| 2016 | 111.0 |
| 2017 | 126.0 |
| 2018 | 164.0 |2020
Multidisciplinary clinical visits starting (including Neuropediatricians/geneticists and psychiatrists)
MLPA/FISH or Karyotyping
Patients from Argentina
Spanish PMS Assoc./INGEMM Agreement
aCGH in INGEMM Lab routine
Patients from Argentina and Colombia
242 cases
130
112 1.16:1
(210 cases with data for analysis)
 99 1.12:1
111
178 cases
32 cases
14 1.285:1
93
18
85 1.09:1
2
6
1
3
4
2
2
22
4
1
28
2
54
13
1
15
7
2
15
1
21
4
Supplementary Figure S1. Distribution of the PMS cohort. A) Distribution of our cohort B) Patient’s recruitment within the project from 2007-2020.
