# Supplementary material for: Variability in Phelan-McDermid Syndrome in a Cohort of 210 Individuals
Source: Front Genet. 2022 Apr 12;13:652454. doi: 10.3389/fgene.2022.652454 (PMC9044489; doi:10.3389/fgene.2022.652454)
Supplement: Supplementary file 3 [file Presentation4.PPTX]

## Slide 1
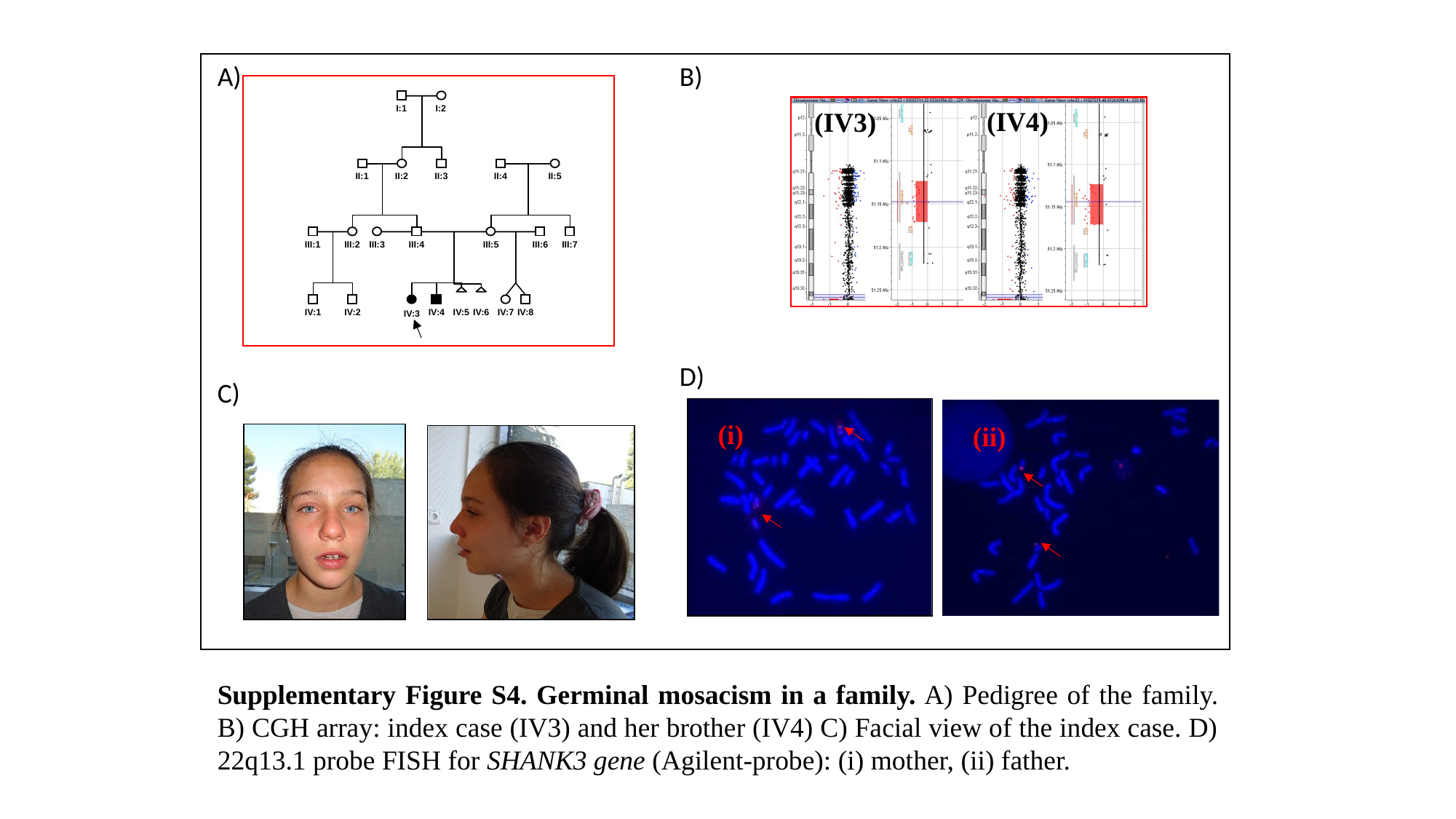

A)
B)
I:1
I:2
II:1
II:2
II:3
II:4
II:5
III:1
III:2
III:3
III:4
III:5
III:6
III:7
IV:1
IV:2
IV:4
IV:5
IV:6
IV:7
IV:8
IV:3
(IV4)
(IV3)
D)
C)
(i)
(ii)
Supplementary Figure S4. Germinal mosacism in a family. A) Pedigree of the family. B) CGH array: index case (IV3) and her brother (IV4) C) Facial view of the index case. D) 22q13.1 probe FISH for SHANK3 gene (Agilent-probe): (i) mother, (ii) father.
