# Supplementary material for: Variability in Phelan-McDermid Syndrome in a Cohort of 210 Individuals
Source: Front Genet. 2022 Apr 12;13:652454. doi: 10.3389/fgene.2022.652454 (PMC9044489; doi:10.3389/fgene.2022.652454)
Supplement: Supplementary file 4 [file Presentation6.PPTX]

## Slide 1
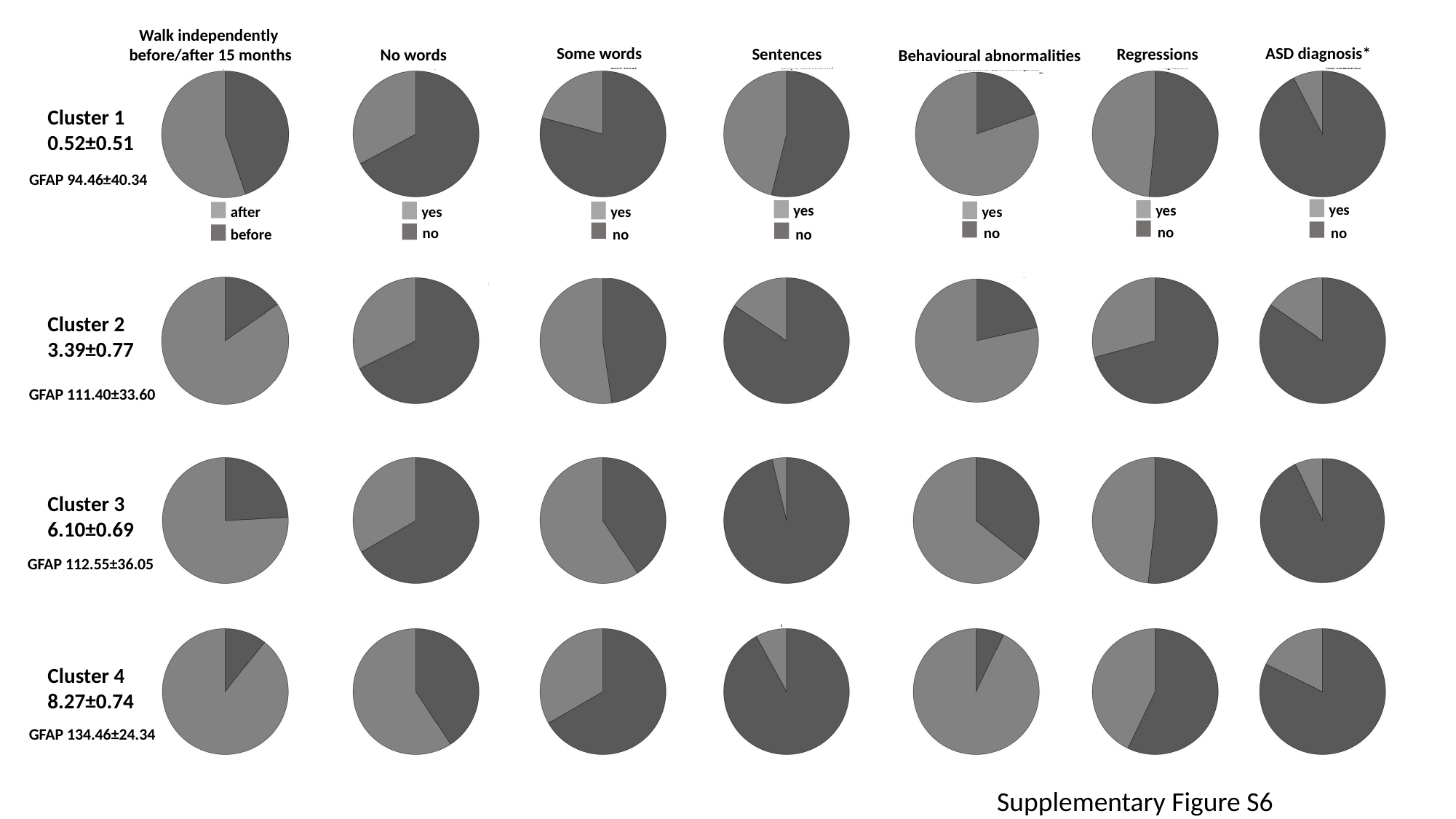

Walk independently
before/after 15 months
Some words
ASD diagnosis*
Sentences
Regressions
No words
Behavioural abnormalities
Cluster 1
0.52±0.51
GFAP 94.46±40.34
yes
yes
yes
after
yes
yes
yes
no
no
no
no
before
no
no
Cluster 2
3.39±0.77
GFAP 111.40±33.60
Cluster 3
6.10±0.69
GFAP 112.55±36.05
Cluster 4
8.27±0.74
GFAP 134.46±24.34
Supplementary Figure S6
