# Supplementary material for: Variability in Phelan-McDermid Syndrome in a Cohort of 210 Individuals
Source: Front Genet. 2022 Apr 12;13:652454. doi: 10.3389/fgene.2022.652454 (PMC9044489; doi:10.3389/fgene.2022.652454)
Supplement: Supplementary file 5 [file Table3.DOCX]

**Supplementary Table S3. Frequencies of the variables on different Clusters identified by Ward´s Cluster analysis based on deletion size.**

| **Variable** | **Cluster 1** | **Cluster 2** | **Cluster 3** | **Cluster 4** |
| --- | --- | --- | --- | --- |
| Gender | 34F/32M | 37F/29M | 14F/15M | 19F/9M |
| Size of deletion (Mb) | Mean: 0.5±0.5  (median, 0.2)  range 0.02-1.9 | Mean: 3.40±0.7  (median, 3.4)  range 2.0-4.7 | Mean: 6.1±0.76  (median, 4)  (range 5.0-7.1 | Mean: 8.2±0.7  (median, 8.0)  range 7.2-10.3 |
| Additional rearrangement | 16/66 (23.9%) | 19/66 (28.8%) | 5/29 (17.2%) | 4/28 (14.3%) |
| Walk independently before 15 months | 30/66 (45.5%) | 10/65 (15.4%) | 7/29 (24.10%) | 3/28 (10.7%) |
| Non verbally fluent | 22/66(33.3%) | 21/65 (32.3%) | 9/27 (33.3%) | 16/27 (59.3%) |
| Some words | 14/66 (21.1%) | 34/65 (52.3%) | 16/27 (59.3%) | 9/27 (33.3%) |
| Ability to make sentences | 30/65 (44.8%) | 10/64 (15.6%) | 1/27 (3.7%) | 2/25 (8.0%) |
| Hypotonia | 41/65 (61.2%) | 50/65 (76.9%) | 26/28 (92.9%) | 25/28 (89.3%) |
| Behaviour alterations | 53/66 (44.8%) | 51/65 (78.5%) | 18/28 (64.3%) | 26/28 (92.9%) |
| Regressions | 32/66 (79.1%) | 32/65 (49.2%) | 14/29 (48.3%) | 12/28 (42.9%) |
| Seizures | 16/66 (24.2%) | 19/65 (29.2%) | 10/28 (35.7%) | 13/28 (46.4%) |
| High pain threshold | 45/66 (68.2%) | 41/65 (63.1%) | 17/28 (60.7%) | 22/28 (78.6%) |
| Decreased perspiration | 33/66 (50.0%) | 36/65 (55.4%) | 16/29 (55.2%) | 14/28 (50.0%) |
| Increased perspiration | 3/66 (4.5%) | 4/65 (6.2%) | 3/28 (3.7%) | 1/28 (3.6%) |
| Microcephaly | 16/66 (24.2%) | 17/65 (25.8%) | 0/29 (0.0%) | 3/28 (10.7%) |
| Macrocephaly | 13/66 (19.7%) | 11/65 (16.9%) | 12/29 (41.4%) | 13/28 (46.0%) |
| GFAP | Mean:94±40.2  (median, 92) | Mean:111.5±37 (median, 114.8) | Mean:112±37 (median, 108) | Mean: 135±24.7 (median, 139.3) |
| Dolichocephaly | 10/66 (15.2%) | 12/65 (18.5%) | 8/29 (27.6%) | 8/28 (28.6%) |
| Flat midface | 8/66 (12.1%) | 8/65 (12.3%) | 2/29 (7.1%) | 8/28 (28.6%) |
| Epicanthal folds | 22/66 (33.3%) | 14/65 (21.5%) | 10/29 (34.5%) | 8/28 (28.6%) |
| Strabismus | 14/66 (21.2%) | 14/65 (21.5%) | 9/28 (32.1%) | 12/28 (42.9%) |
| Ptosis | 12/66 (18.2%) | 11/66 (16.9%) | 3/28 (10.7%) | 8/28 (28.6%) |
| Deep set eyes | 11/66 (16.7%) | 13/66 (20.0%) | 11/28 (39.3%) | 9/28 (32.1%) |
| Long eyelashes | 37/66 (55.2%) | 33/66 (50.8%) | 17/29 (60.7%) | 20/28 (71.4%) |
| Full brow | 22/66 (33.3%) | 27/66 (41.5%) | 11/28 (39.3%) | 14/28 (50.0%) |
| Full/puffy eyelids | 8/66 (12.1%) | 19/65 (29.2%) | 8/28 (28.6%) | 8/28 (28.6%) |
| Wide nasal bridge | 34/66 (51.5%) | 33/65 (50.8%) | 16/28 (57.1%) | 22/28 (78.6%) |
| Bulbous nose | 36/66 (54.5%) | 36/65 (55.4%) | 19/28 (67.9%) | 17/28 (60.7%) |
| Ear anomalies | 25/66 (37.9%) | 27/65 (41.5%) | 16/28 (57.1%) | 19/28 (67.9%) |
| Full/puffy cheeks | 9/66 (13.6%) | 19/65 (29.2%) | 6/28 (21.4%) | 8/28 (28.6%) |
| Dental anomalies | 31/66 (47.0%) | 27/65 (41.5%) | 12/28 (42.9%) | 18/28 (64.3%) |
| Pointed chin | 45/66 (68.2%) | 32/65 (49.2%) | 16/28 (57.1%) | 16/28 (53.1%) |
| Toe syndactyly | 12/66 (18.2%) | 17/65(26.2%) | 10/28 (35.7%) | 12/28 (42.9%) |
| Hypoplastic/dysplastic nails | 24/66 (36.4%) | 26/65(40.0%) | 12/28 (42.9%) | 14/28 (50.0%) |
| Large, fleshy hands | 30/66 (45.5%) | 32/65 (49.2%) | 20/28 (71.4%) | 19/28 (67.9%) |
| Fifth finger clinodactyly | 13/66 (19.7%) | 12/65 (18.5%) | 4/28 (14.3%) | 6/28 (21.4%) |
| Cardiac anomalies | 10/66 (15.2%) | 10/65 (15.2%) | 5/28 (17.2%) | 5/28 (17.9%) |
| Sleeping problems | 16/66 (24.2%) | 16/65 (24.6%) | 3/28 (10.7%) | 10/28 (35.7%) |
| Ophthalmological problems | 12/66 (18.2%) | 12/66 (18.5%) | 9/28 (32.1%) | 8/28 (28.6%) |
| Renal and urological abnormalities | 10/66 (15.20%) | 13/65 (19.7%) | 6/28 (21.4%) | 13/28 (46.4%) |
| Lip/palate abnormalities | 4/66 (6.1%) | 5/65 (7.7%) | 2/28 (6.9%) | 6/28 (21.4%) |
| Skin anomalies | 11/66 (16.7%) | 14/65 (21.5%) | 8/28 (28.6%) | 9/28 (32.1%) |
| Recurrent infections | 9/66 (13.6%) | 13/65 (20.0%) | 6/28 (21.4%) | 2/28 (7.1%) |
| Herniae | 1/66 (1.5%) | 4/65 (6.2%) | 4/28 (14.3%) | 3/28 (10.7%) |
| Brain MRI studies | 25/65 (38.5%) | 38/65 (58.5%) | 15/29 (51.7%) | 17/28 (60.7%) |
| Brain MRI anomalies | 7/25 (28.0%) | 14/38 (36.8%) | 6/15 (40.0%) | 9/17 (52.1%) |
| Obesity | 0/66 (0%) | 1/65 (1.5%) | 1/29 (3.4%) | 2/28 (7.1%) |
| Hearing problems | 7/65 (10.6%) | 5/65 (7.7%) | 2/28 (7.1%) | 6/28 (21.4%) |
| Lymphedema | 3/65 (4.5%) | 2/65 (3.1%) | 3/28 (10.7%) | 10/28 (35.7%) |
| Gastrointestinal problems | 11/65 (16.7%) | 14/65 (21.5%) | 1/28 (3.6%) | 8/28 (28.6%) |
| Poor visual contact | 32/65 (48.5%) | 39/65 (60.0%) | 15/28 (53.6%) | 20/28 (71.4%) |
| Biting | 18/65 (26.9%) | 32/65 (49.2%) | 8/28 (28.6%) | 12/28 (42.9%) |
| Very sensitive to touch | 17/65 (35.8%) | 25/65 (38.5%) | 9/28 (32.1%) | 10/28 (35.7%) |
| Uncontrolled laughter | 19/66 (28.8%) | 24/65 (36.9%) | 10/28 (35.7%) | 16/28 (57.1%) |
| Impulsive | 36/66 (54.5%) | 33/65 (50.8%) | 14/28 (50.0%) | 14/28 (50.0%) |
| Excessive yelling | 23/66 (35.4 %) | 22/65 (33.8%) | 8/28 (28.6%) | 16/28 (57.1%) |
| Hair pulling | 11/66 (16.7%) | 15/65 (23.1%) | 7/28 (25.0%) | 9/28 (32.1%) |
| Skin picking | 14/66 (21.2%) | 17/65 (26.1%) | 6/28 (21.4%) | 6/28 (21.4%) |
| Non-stop crying | 8/66 (12.1%) | 11/65 (16.9%) | 0/28 (0.0%) | 7/28 (25.0%) |
| Aggressive | 12/66 (18.2%) | 15/65 (23.1%) | 6/28 (21.4%) | 3/28 (3.7%) |
| Tongue thrusting | 25/66 (37.9%) | 17/65 (26.10%) | 10/28 (35.7%) | 10/28 (35.7%) |
| Abnormal emotional response | 40/66 (60.6%) | 35/65 (53.8%) | 11/28 (39.3%) | 12/28 (42.9%) |
| Formal ASD evaluation | 12/66 (18.2%) | 12/65 (18.5%) | 6/27 (22.4%) | 5/28 (17.9%) |
| ASD diagnosis* | 5/12 (41.6%) | 10/12 (83.3%) | 2/6 (33.3%) | 5/5 (100.0%) |
| Age at diagnosis (months) | Mean:94.1±80.8 (Median, 74)  range 6-454 | Mean: 64.1±85 (Median, 36)  range 1-552 | Mean: 75.2±110.3 (Median, 36) range 1-434 | Mean: 28.2±55.7 (Median, 9.5) range 1-264 |
| Growth centile > 95% | 19/67 (28.4%) | 16/66 (24.2%) | 9/29 (31.0%) | 16/28 (57.1%) |
| Growth centile < 3% | 5/67 (7.5%) | 12/66 (18.2%) | 3/29 (10.3%) | 3/28 (10.7%) |

See Figure 6C for significant statistical differences between clusters pairs. ASD, autism spectrum disorders; F, female; Mb, megabase; MRI, magnetic resonance image; GFAP, global functional assessment of the patient; M, male. *ASD diagnosis according to the psychiatrists of the referring institutions.
