# Supplementary material for: Variability in Phelan-McDermid Syndrome in a Cohort of 210 Individuals
Source: Front Genet. 2022 Apr 12;13:652454. doi: 10.3389/fgene.2022.652454 (PMC9044489; doi:10.3389/fgene.2022.652454)
Supplement: Supplementary file 7 [file Table4.DOCX]

**Supplementary Table S4.** Description and frequencies of clinical features of individuals with 22q13.3 deletions that do not include *SHANK3* gene. Three patients are included from this cohort (PMS59, PMS114, PMS136), and 14 were reported previously (Wilson et al., 2008; Disciglio et al.; 2014 and Ha et al., 2017).

1. **Categorical variables**

|  | | | **Frequency** | **Percentage** |
| --- | --- | --- | --- | --- |
| **Gender** | | Male | 8 | 57.1 |
|  |  | Female | 6 | 42.9 |
|  |  | Total | 14 | 100 |
|  | | |  |  |
| **Growth** | | Centile <95th | 5 | 38.5 |
|  |  | Normal | 2 | 15.4 |
|  |  | Centile >95th | 6 | 46.2 |
|  |  | Total | 13 | 100 |
|  | | |  |  |
| **Developmental delay** | | Walk independently > 15 months | 15 | 93.8 |
|  |  | Walk independently < 15 months | 1 | 6.2 |
|  |  | Total | 16 | 100 |
|  | | |  |  |
| **Delayed or absent speech** | | No  Yes  Total | 0  16  16 | 0  100  100 |
|  | | |  |  |
| **Hypotonia** | | No | 4 | 26.7 |
|  |  | Yes | 11 | 73.3 |
|  |  | Total | 15 | 100 |
|  | | |  |  |
| **Behavioral abnormalities** | | No  Yes  Total | 0  3  3 | 0  100  100 |
|  | | |  |  |
| **Regression** | | No | 2 | 66.7 |
|  |  | Yes | 1 | 33.3 |
|  |  | Total | 3 | 100 |
|  | | |  |  |
| **Seizures** | | No | 13 | 86.7 |
|  |  | Yes | 2 | 13.3 |
|  |  | Total | 15 | 100 |
|  | | |  |  |
| **High pain threshold** | | No | 2 | 66.7 |
|  |  | Yes | 1 | 33.3 |
|  |  | Total | 3 | 100 |
|  | | |  |  |
| **Decreased perspiration** | | No | 2 | 66.7 |
|  |  | Yes | 1 | 33.3 |
|  |  | Total | 3 | 100 |
|  | | |  |  |
| **Macrocephaly** | | No | 9 | 52.9 |
|  |  | Yes | 8 | 47.1 |
|  |  | Total | 17 | 100 |
| **Microcephaly** | | No | 4 | 80 |
|  |  | Yes | 1 | 20 |
|  |  | Total | 5 | 100 |
|  | | |  |  |
| **Dolichocephaly** | | No | 3 | 75 |
|  |  | Yes | 1 | 25 |
|  |  | Total | 4 | 100 |
|  | | |  |  |
| **Flat midface** | | No  Yes  Total | 3  0  3 | 100  0  100 |
|  | | |  |  |
| **Epicanthal folds** | | No | 2 | 40 |
|  |  | Yes | 3 | 60 |
|  |  | Total | 5 | 100 |
|  | | |  |  |
| **Strabismus** | | No | 3 | 75 |
|  |  | Yes | 1 | 25 |
|  |  | Total | 4 | 100 |
|  | | |  |  |
| **Ptosis** | | No | 2 | 50 |
|  |  | Yes | 2 | 50 |
|  |  | Total | 4 | 100 |
|  | | |  |  |
| **Deep set eyes** No | | | 3 | 42.85 |
| Yes | | | 4 | 57.15 |
| Total | | | 7 | 100 |
|  | | |  |  |
| **Long eye lashes** | | No | 2 | 66.7 |
|  |  | Yes | 1 | 33.3 |
|  |  | Total | 3 | 100 |
|  | | |  |  |
| **Full brow** | | No | 2 | 66.7 |
|  |  | Yes | 1 | 33.3 |
|  |  | Total | 3 | 100 |
|  | | |  |  |
| **Full/puffy eyelids** | | No | 2 | 66.7 |
|  |  | Yes | 1 | 33.3 |
|  |  | Total | 3 | 100 |
|  | | |  |  |
| **Wide nasal bridge** | | No | 3 | 42.9 |
|  |  | Yes | 4 | 57.1 |
|  |  | Total | 7 | 100 |
|  | | |  |  |
| **Bulbous nose** | | No | 2 | 40 |
|  |  | Yes | 3 | 60 |
|  |  | Total | 5 | 100 |
|  | | |  |  |
| **Ears anomalies** | | No | 2 | 28.6 |
|  |  | Yes | 5 | 71.4 |
|  |  | Total | 7 | 100 |
|  | | |  |  |
| **Full puffy cheeks** | | No | 3 | 75 |
|  |  | Yes | 1 | 25 |
|  |  | Total | 4 | 100 |
|  | | |  |  |
| **Widely spaced teeth, maloclussion** | | No | 2 | 66.7 |
|  |  | Yes | 1 | 33.3 |
|  |  | Total | 3 | 100 |
|  | | |  |  |
| **Pointed chin** | | No | 1 | 20 |
|  |  | Yes | 4 | 80 |
|  |  | Total | 5 | 100 |
|  | | |  |  |
| **Toe syndactyly** | | No  Yes  Total | 3  0  3 | 100  0  100 |
|  | | |  |  |
| **Hypoplastic/dysplastic toenails** | | No | 2 | 66.7 |
|  |  | Yes | 1 | 33.3 |
|  |  | Total | 3 | 100.0 |
|  | | |  |  |
| **Large fleshy hands** | | No | 3 | 60 |
|  |  | Yes | 2 | 40 |
|  |  | Total | 5 | 100 |
|  | | |  |  |
| **Cardiac disease** | | No | 3 | 42.9 |
|  |  | Yes | 4 | 57.1 |
|  |  | Total | 7 | 100 |
|  | | |  |  |
| **Ophthalmological anomalies** | | No | 3 | 21.42 |
| Yes | | | 14 | 78.38 |
| Total | | | 17 | 100 |
| **Sphincter control** | | No  Yes | 3 | 21.42 |
|  | | Total | 14 | 78.38 |
|  | | | 17 | 100 |
| **Renal and urological abnormalities** | | No | 13 | 81.3 |
|  |  | Yes | 3 | 18.8 |
|  |  | Total | 16 | 100 |
|  | | |  |  |
| **Lip/palate anomalies** | | No | 3 | 75.0 |
|  |  | Yes | 1 | 25.0 |
|  |  | Total | 4 | 100 |
|  | | |  |  |
| **Sleep disturbances** | | No | 4 | 80.0 |
|  |  | Yes | 1 | 20.0 |
|  |  | Total | 5 | 100 |
|  | | |  |  |
| **Skin anomalies** | | No  Yes  total | 3  0  3 | 100  0  100 |
|  | | |  |  |
| **Recurrent infections** | | No | 3 | 60,0 |
|  |  | Yes | 2 | 40,0 |
|  |  | Total | 5 | 100,0 |
|  | | |  |  |
| **Herniae** | | No  Yes  Total | 3  0  3 | 100  0  100 |
|  | | |  |  |
| **Brain MRI anomalies** | | No | 6 | 42.9 |
|  |  | Yes | 8 | 57.1 |
|  |  | Total | 14 | 100 |
|  | | |  |  |
| **Obesity** | | No | 2 | 66.7 |
|  |  | Yes | 1 | 33.3 |
|  |  | Total | 3 | 100 |
|  | | |  |  |
| **Hearing problems** | | No | 2 | 40 |
|  |  | Yes | 3 | 60 |
|  |  | Total | 5 | 100 |
|  | | |  |  |
| **Lymphedema** | | No  Yes  Total | 3  0  3 | 100  0  100 |
|  | | |  |  |
| **Gastrointestinal anomalies** | | No | 5 | 45.5 |
|  |  | Yes | 6 | 54.5 |
|  |  | Total | 11 | 100 |
|  | | |  |  |
| **Poor visual contact** | | No | 1 | 33.3 |
|  |  | Yes | 2 | 66.7 |
|  |  | Total | 3 | 100 |
|  | | |  |  |
| **Biting** | | No | 2 | 66.7 |
|  |  | Yes | 1 | 33.3 |
|  |  | Total | 3 | 100 |
|  | | |  |  |
| **Very sensitive to touch** | | No  Yes  Total | 3  0  3 | 100  0  100 |
|  | | |  |  |
| **Uncontrolled laughter** | No  Yes  Total | | 3  0  3 | 100  0  100 |
|  | | |  |  |
| **Impulsive** | | No | 1 | 33.3 |
|  |  | Yes | 2 | 66.7 |
|  |  | Total | 3 | 100.0 |
|  | | |  |  |
| **Excess yelling** | | No  Yes  Total | 3  0  3 | 100  0  100 |
|  | | |  |  |
| **Hair pulling** | | No  Yes  Total | 3  0  3 | 100  0  100 |
|  | | |  |  |
| **Non-stop crying** | | No  Yes  Total | 3  0  3 | 100  0  100 |
|  | | |  |  |
| **Aggressive behavior** | | No  Yes  Total | 3  0  3 | 100  0  100 |
|  | | |  |  |
| **Tongue thrusting** | | No  Yes  Total | 3  0  3 | 100  0  100 |
|  | | |  |  |
| **Abnormal emotional response** | | No | 2 | 66.7 |
|  |  | Yes | 1 | 33.3 |
|  |  | Total | 3 | 100 |
|  | | |  |  |
| **ASD diagnosis*** | | No | 3 | 50.0 |
|  |  | Yes | 3 | 25.0 |
|  |  | Total | 9 | 100 |
|  |  |  |  |  |
|  | | |  |  |
| **Other rearrangements** | | No | 2 | 66.7 |
|  |  | Yes | 1 | 33.3 |
|  |  | Total | 3 | 100.0 |
|  | | |  |  |

*****ASD diagnosis according to the psychiatrists of the referring institutions.

1. **Continuous variables**

|  | | | | | | | | |
| --- | --- | --- | --- | --- | --- | --- | --- | --- |
|  |  |  |  |  |  | |  |  |
|  | **N** | **Range** | **Minimum** | **Maximum** | **Mean** | **Standard error** | **Standard deviation** | **Variance** |
| **Size of deletion (Mb)** | 17 | 6.9 | .003 | 6.9 | 3.9 | .5 | 2.3 | 5.2 |
| **GFAP (arbitrary value)** | 3 | 56.0 | 22.0 | 78.0 | 45.3 | 16.8 | 29.1 | 849.3 |
| **Age at diagnosis (months)** | 3 | 139.0 | 25.0 | 164.0 | 76.0 | 44.2 | 76.5 | 5857.0 |

Mb, megabase; GFAP, global functional assessment of the patient.
