# Supplementary material for: Variability in Phelan-McDermid Syndrome in a Cohort of 210 Individuals
Source: Front Genet. 2022 Apr 12;13:652454. doi: 10.3389/fgene.2022.652454 (PMC9044489; doi:10.3389/fgene.2022.652454)
Supplement: Supplementary file 8 [file Presentation3.PPTX]

## Slide 1
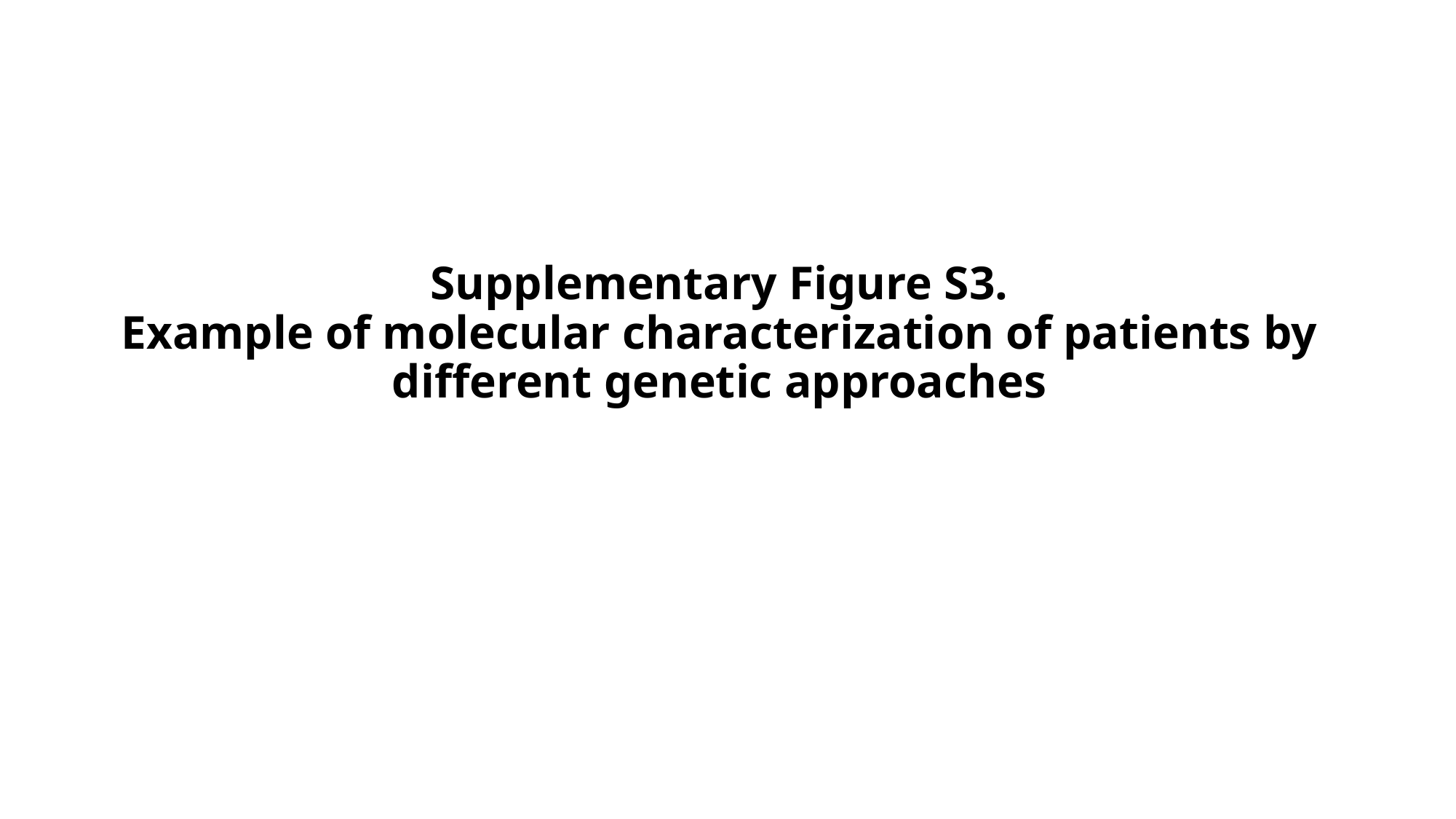

# Supplementary Figure S3. Example of molecular characterization of patients by different genetic approaches

## Slide 2
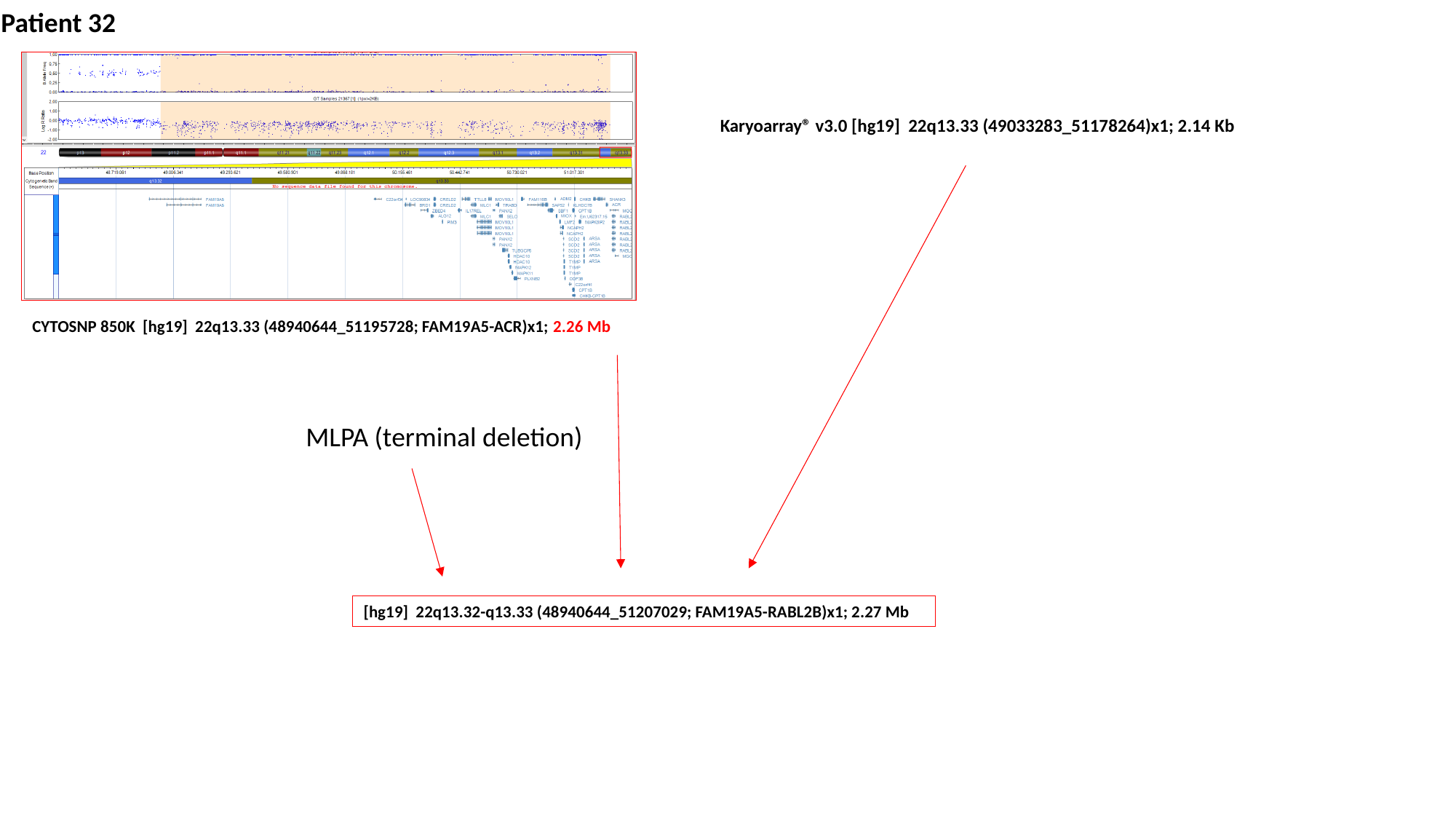

Patient 32
Karyoarray® v3.0 [hg19] 22q13.33 (49033283_51178264)x1; 2.14 Kb
CYTOSNP 850K [hg19] 22q13.33 (48940644_51195728; FAM19A5-ACR)x1; 2.26 Mb
MLPA (terminal deletion)
[hg19] 22q13.32-q13.33 (48940644_51207029; FAM19A5-RABL2B)x1; 2.27 Mb

## Slide 3
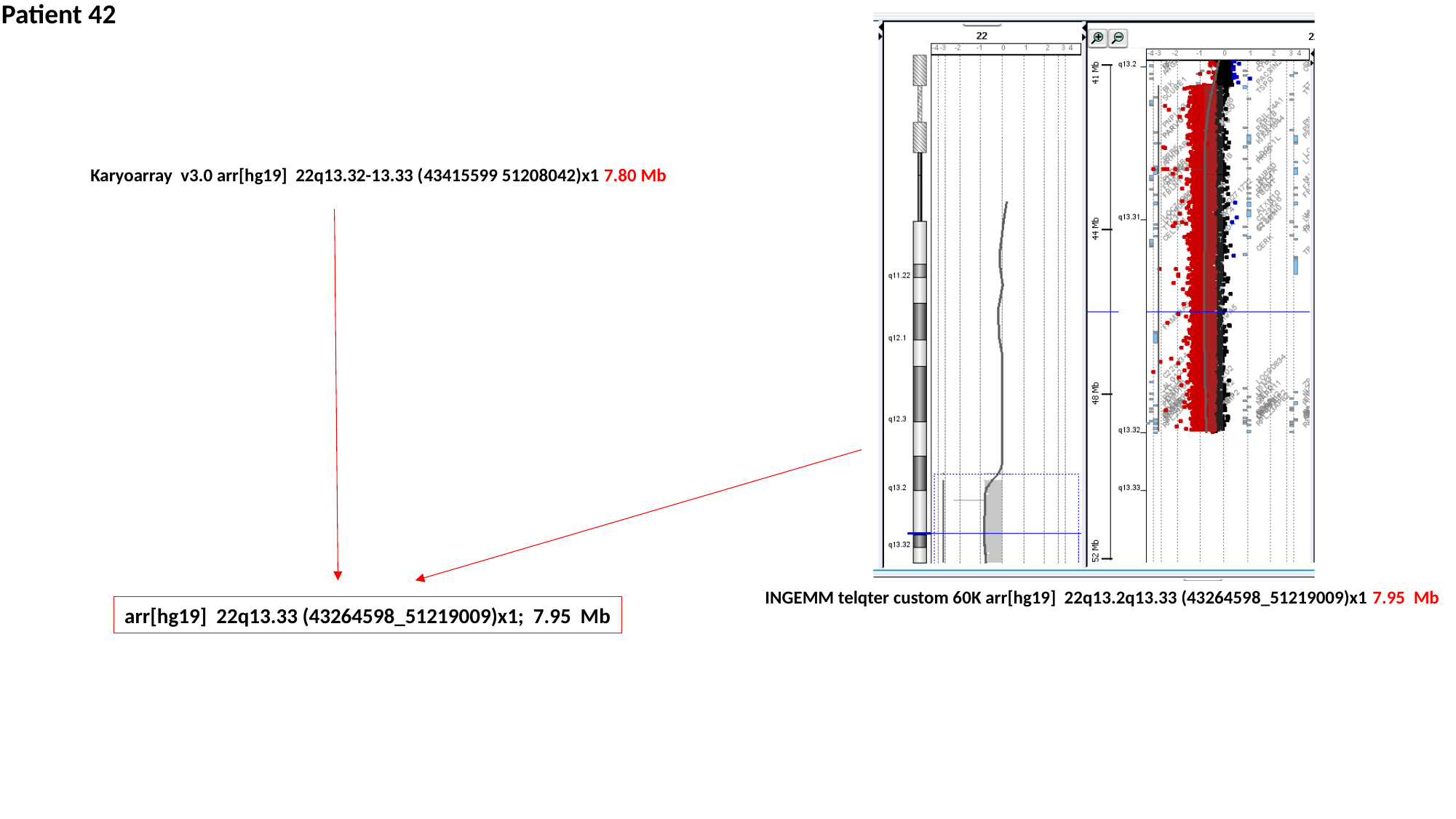

Patient 42
Karyoarray v3.0 arr[hg19] 22q13.32-13.33 (43415599 51208042)x1 7.80 Mb
 INGEMM telqter custom 60K arr[hg19] 22q13.2q13.33 (43264598_51219009)x1 7.95 Mb
arr[hg19] 22q13.33 (43264598_51219009)x1; 7.95 Mb

## Slide 4
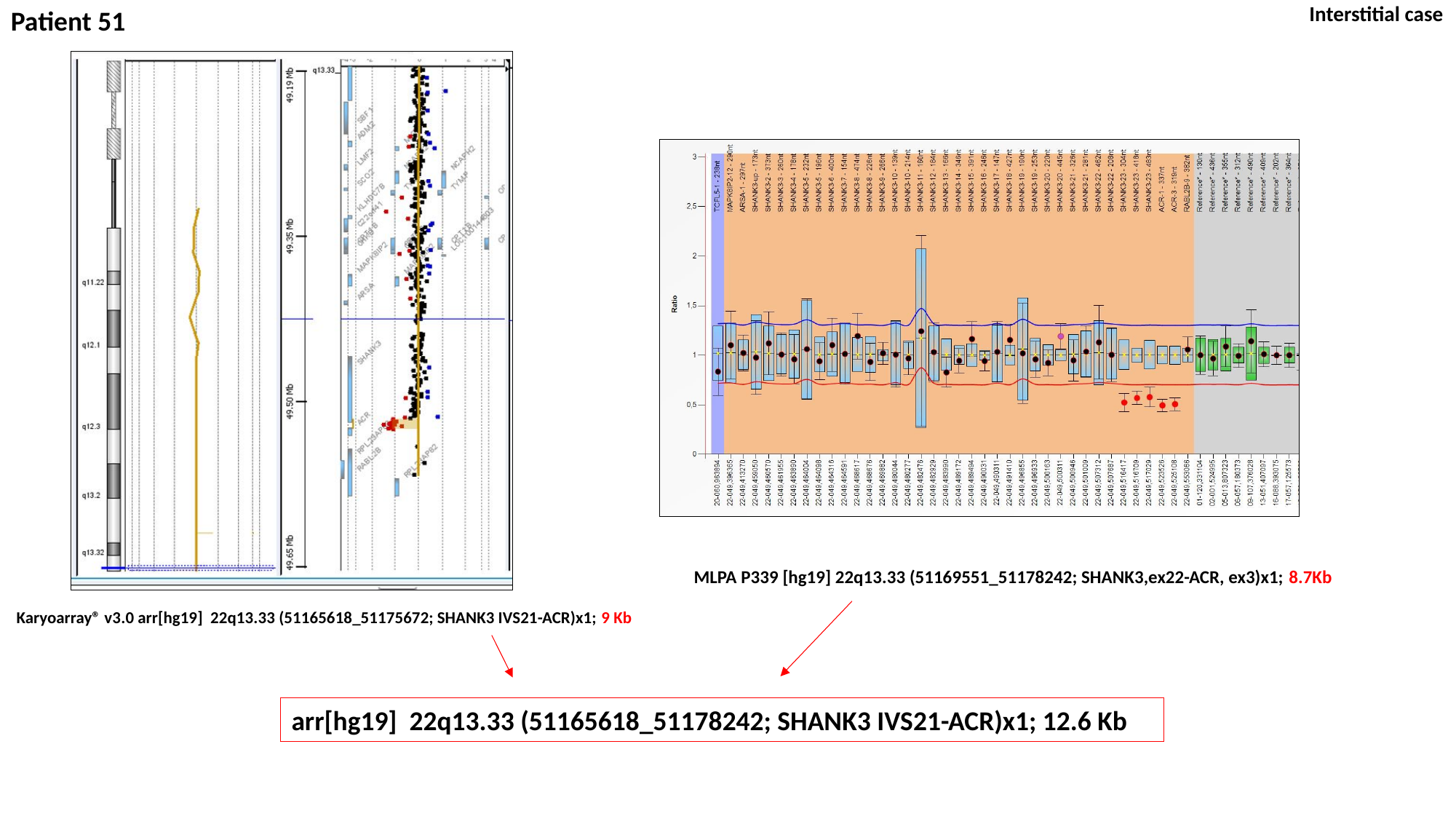

Interstitial case
Patient 51
MLPA P339 [hg19] 22q13.33 (51169551_51178242; SHANK3,ex22-ACR, ex3)x1; 8.7Kb
Karyoarray® v3.0 arr[hg19] 22q13.33 (51165618_51175672; SHANK3 IVS21-ACR)x1; 9 Kb
arr[hg19] 22q13.33 (51165618_51178242; SHANK3 IVS21-ACR)x1; 12.6 Kb

## Slide 5
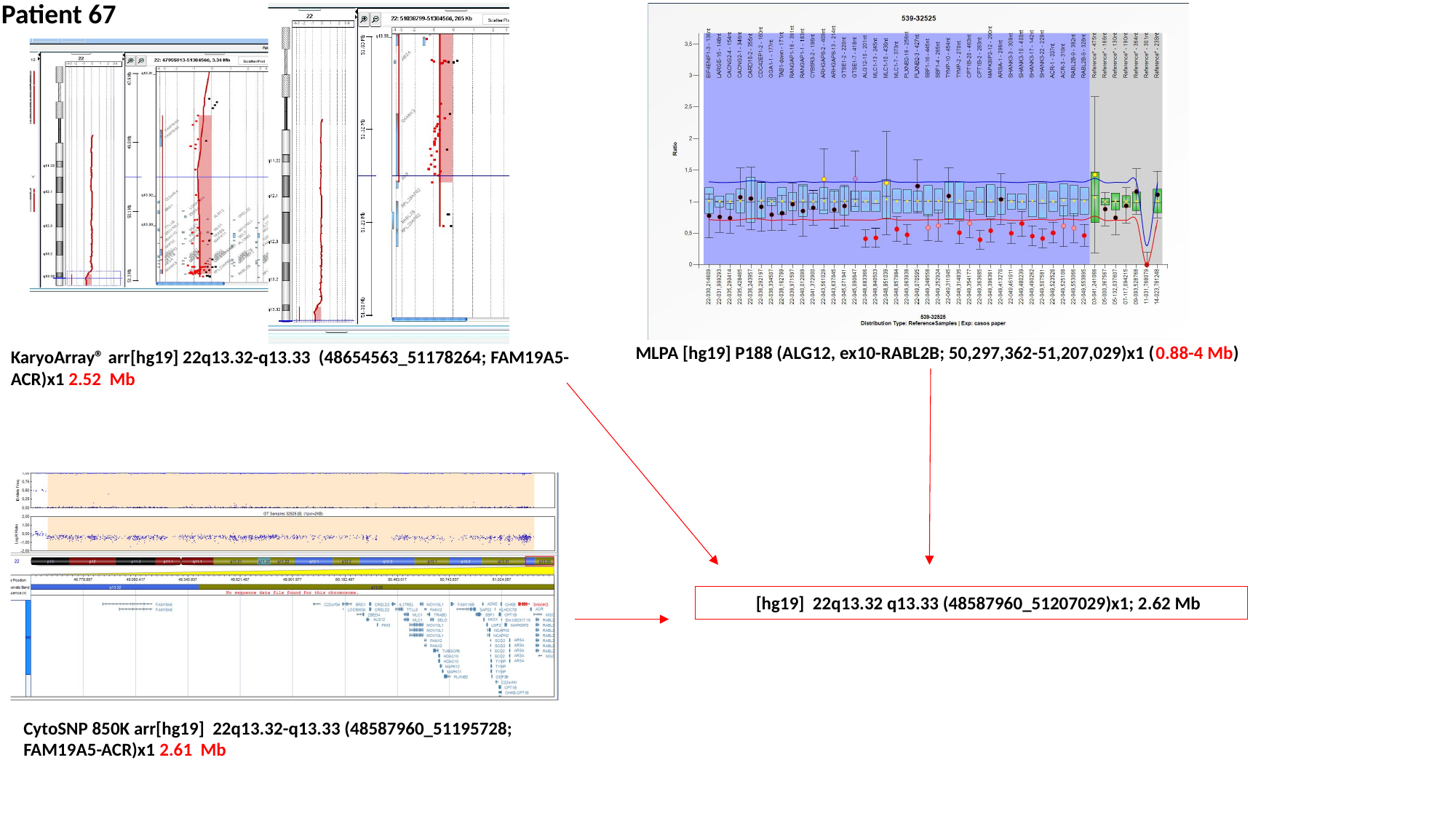

Patient 67
MLPA [hg19] P188 (ALG12, ex10-RABL2B; 50,297,362-51,207,029)x1 (0.88-4 Mb)
KaryoArray® arr[hg19] 22q13.32-q13.33 (48654563_51178264; FAM19A5-ACR)x1 2.52 Mb
 [hg19] 22q13.32 q13.33 (48587960_51207029)x1; 2.62 Mb
CytoSNP 850K arr[hg19] 22q13.32-q13.33 (48587960_51195728; FAM19A5-ACR)x1 2.61 Mb

## Slide 6
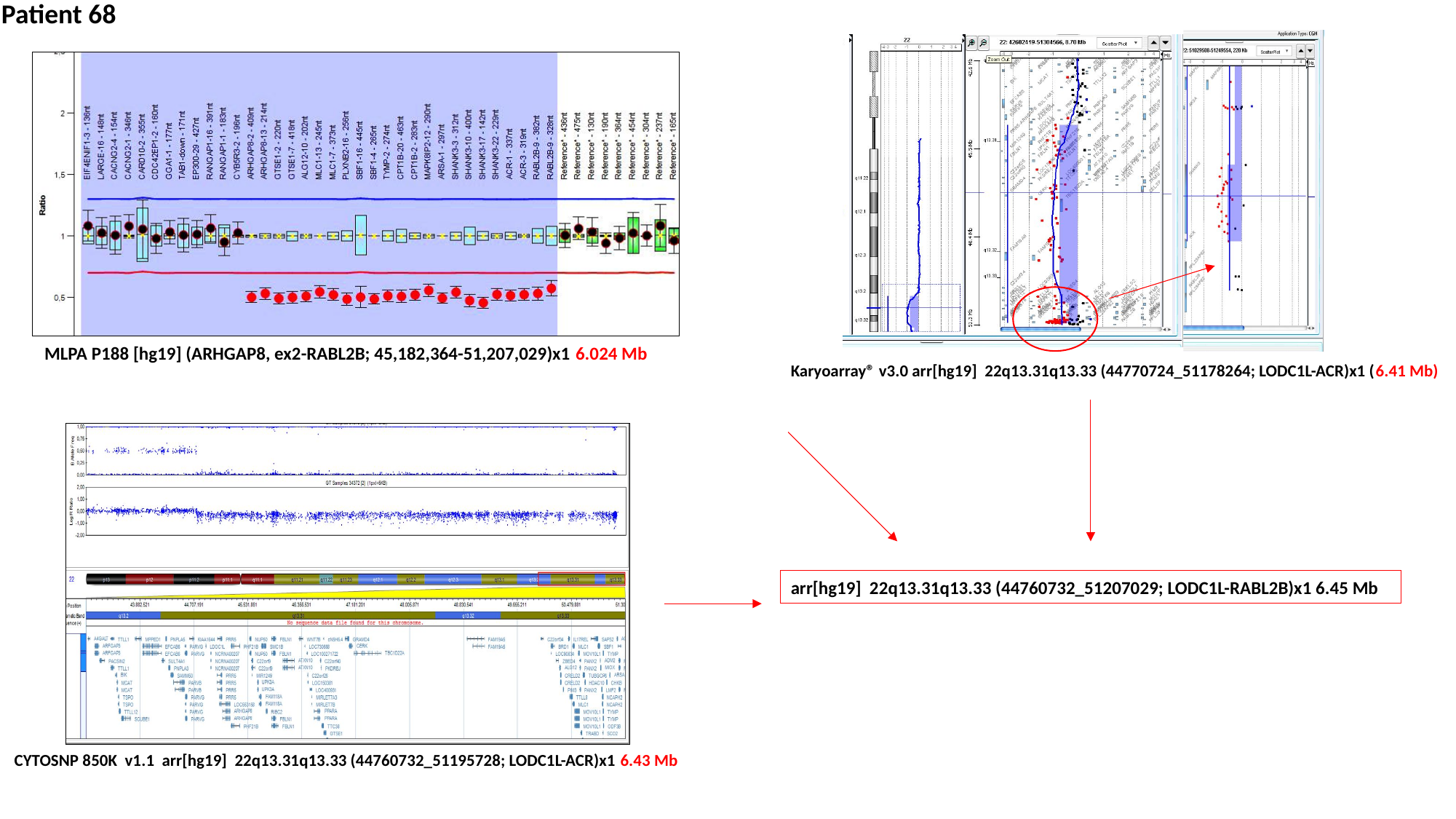

Patient 68
MLPA P188 [hg19] (ARHGAP8, ex2-RABL2B; 45,182,364-51,207,029)x1 6.024 Mb
Karyoarray® v3.0 arr[hg19] 22q13.31q13.33 (44770724_51178264; LODC1L-ACR)x1 (6.41 Mb)
arr[hg19] 22q13.31q13.33 (44760732_51207029; LODC1L-RABL2B)x1 6.45 Mb
 CYTOSNP 850K v1.1 arr[hg19] 22q13.31q13.33 (44760732_51195728; LODC1L-ACR)x1 6.43 Mb

## Slide 7
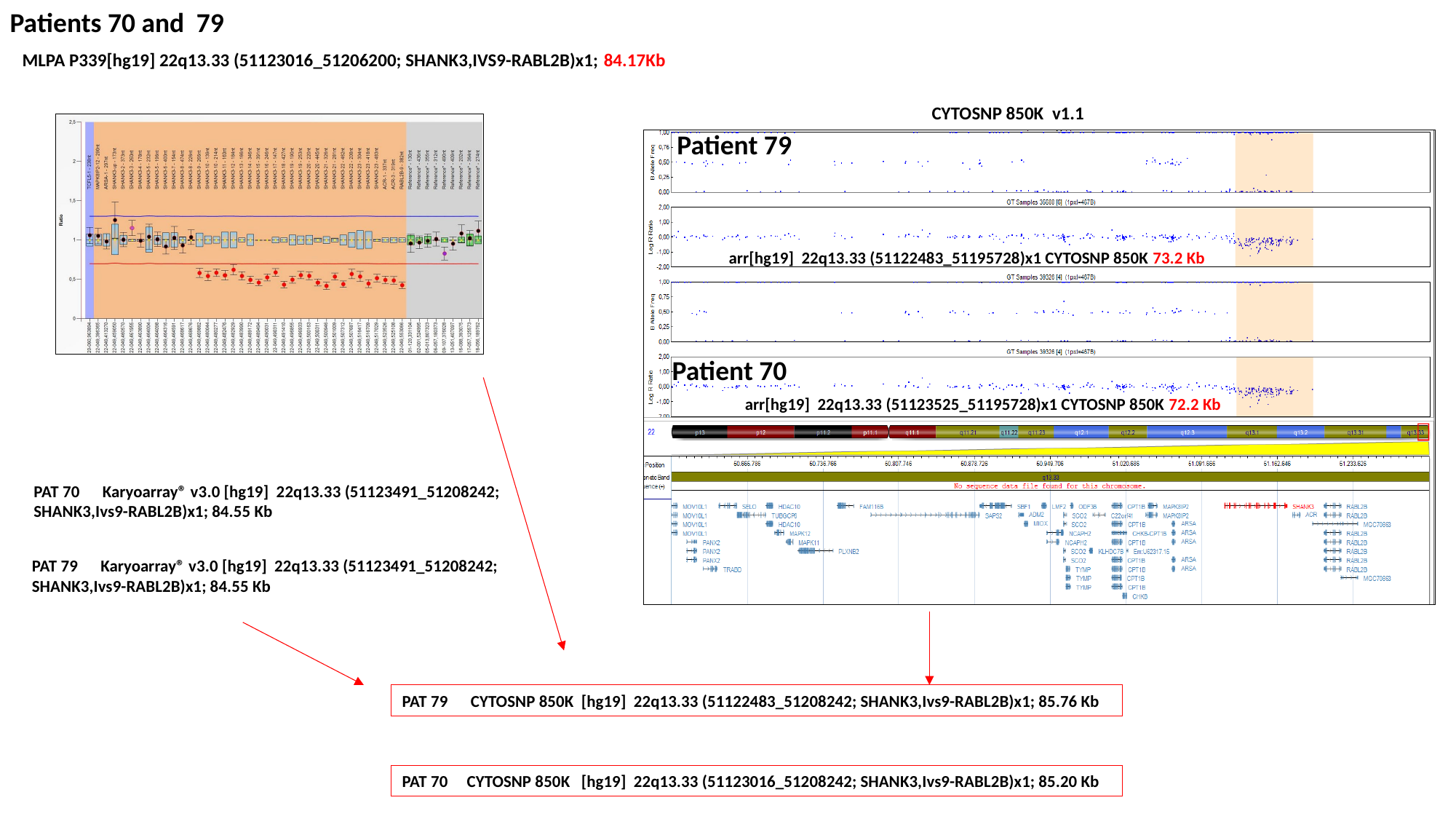

Patients 70 and 79
MLPA P339[hg19] 22q13.33 (51123016_51206200; SHANK3,IVS9-RABL2B)x1; 84.17Kb
CYTOSNP 850K v1.1
Patient 79
arr[hg19] 22q13.33 (51122483_51195728)x1 CYTOSNP 850K 73.2 Kb
Patient 70
arr[hg19] 22q13.33 (51123525_51195728)x1 CYTOSNP 850K 72.2 Kb
PAT 70 Karyoarray® v3.0 [hg19] 22q13.33 (51123491_51208242;
SHANK3,Ivs9-RABL2B)x1; 84.55 Kb
PAT 79 Karyoarray® v3.0 [hg19] 22q13.33 (51123491_51208242;
SHANK3,Ivs9-RABL2B)x1; 84.55 Kb
PAT 79 CYTOSNP 850K [hg19] 22q13.33 (51122483_51208242; SHANK3,Ivs9-RABL2B)x1; 85.76 Kb
PAT 70 CYTOSNP 850K [hg19] 22q13.33 (51123016_51208242; SHANK3,Ivs9-RABL2B)x1; 85.20 Kb

## Slide 8
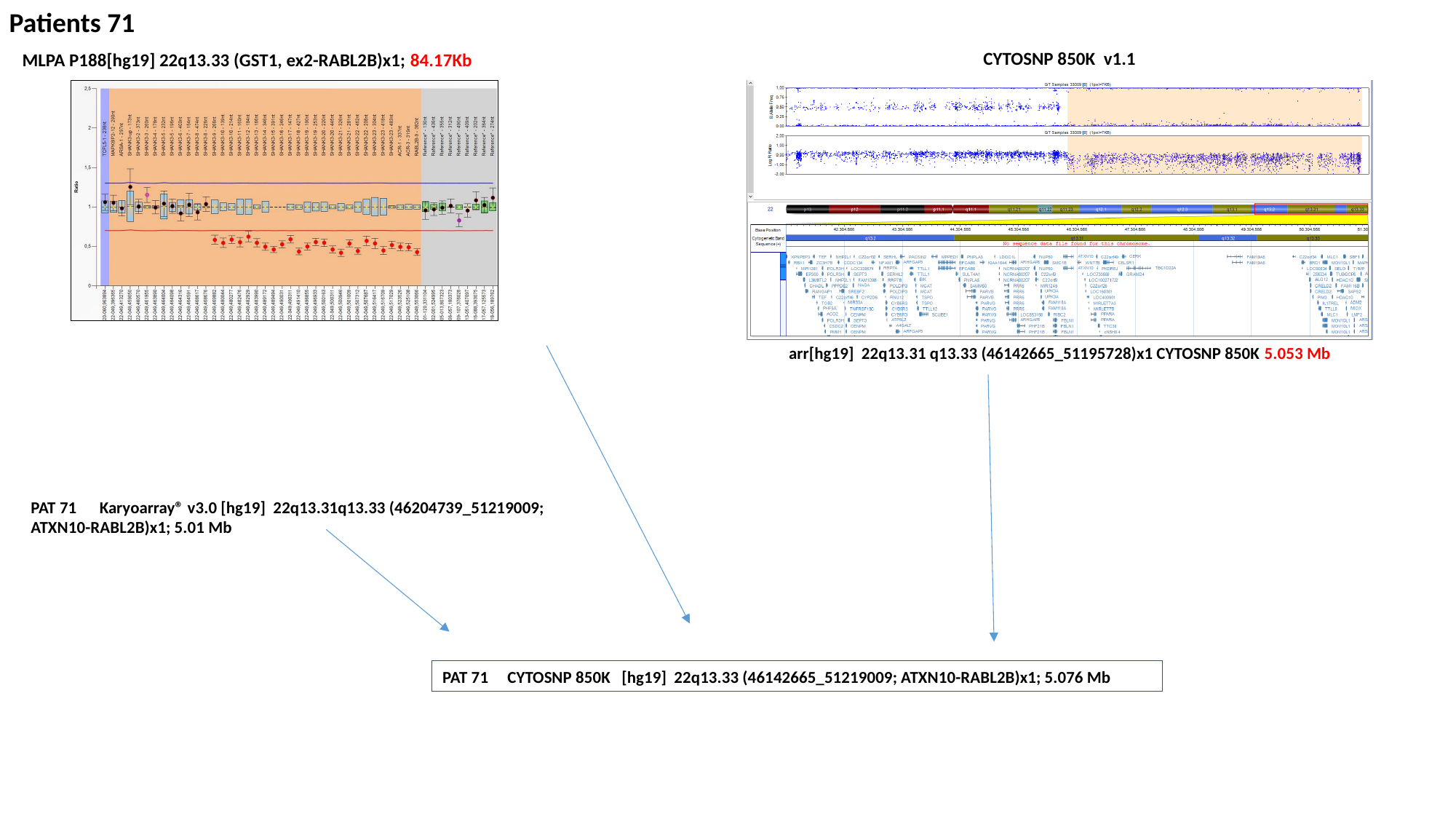

Patients 71
CYTOSNP 850K v1.1
MLPA P188[hg19] 22q13.33 (GST1, ex2-RABL2B)x1; 84.17Kb
arr[hg19] 22q13.31 q13.33 (46142665_51195728)x1 CYTOSNP 850K 5.053 Mb
PAT 71 Karyoarray® v3.0 [hg19] 22q13.31q13.33 (46204739_51219009;
ATXN10-RABL2B)x1; 5.01 Mb
PAT 71 CYTOSNP 850K [hg19] 22q13.33 (46142665_51219009; ATXN10-RABL2B)x1; 5.076 Mb

## Slide 9
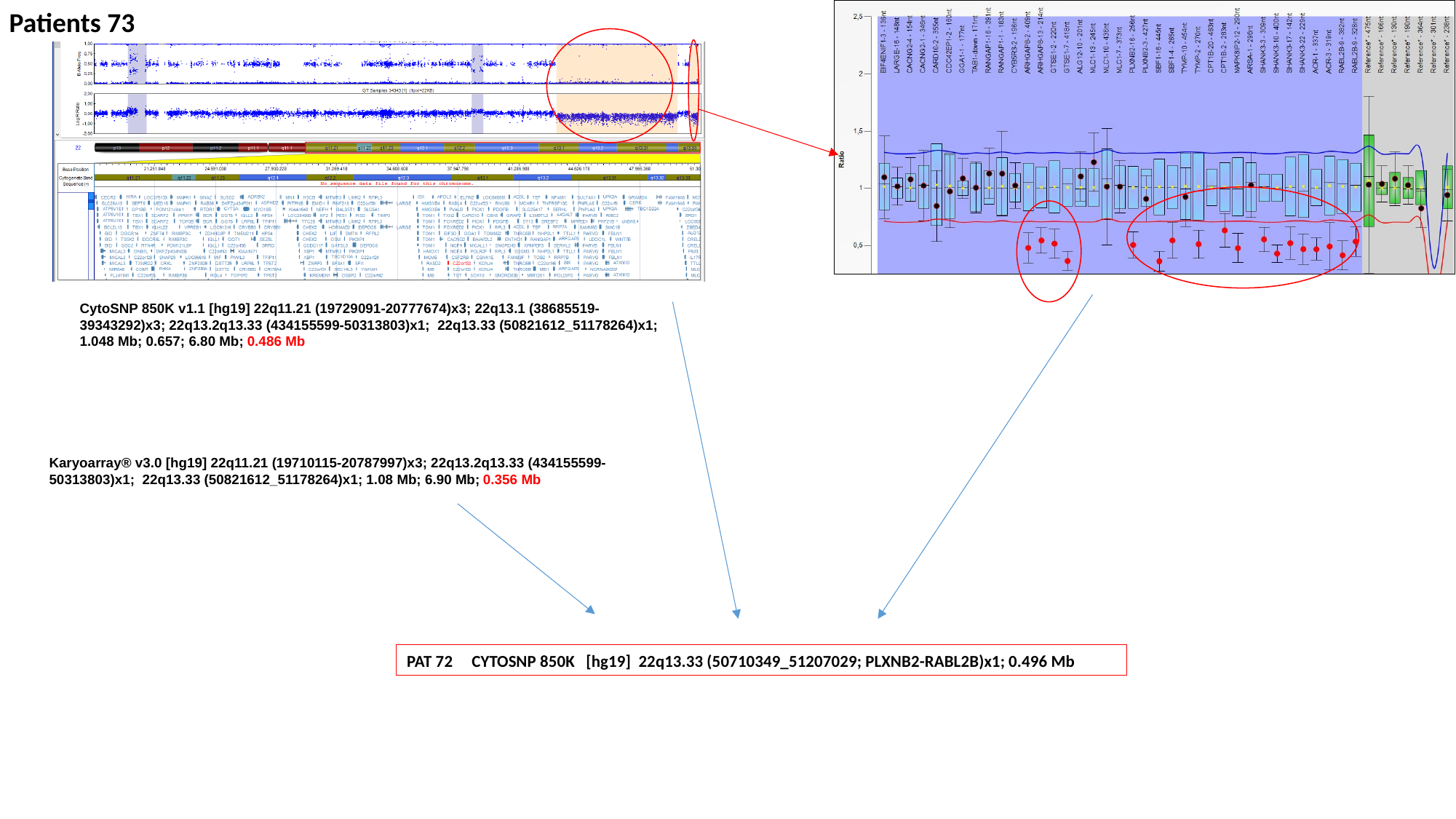

Patients 73
CytoSNP 850K v1.1 [hg19] 22q11.21 (19729091-20777674)x3; 22q13.1 (38685519-39343292)x3; 22q13.2q13.33 (434155599-50313803)x1; 22q13.33 (50821612_51178264)x1; 1.048 Mb; 0.657; 6.80 Mb; 0.486 Mb
Karyoarray® v3.0 [hg19] 22q11.21 (19710115-20787997)x3; 22q13.2q13.33 (434155599-50313803)x1; 22q13.33 (50821612_51178264)x1; 1.08 Mb; 6.90 Mb; 0.356 Mb
PAT 72 CYTOSNP 850K [hg19] 22q13.33 (50710349_51207029; PLXNB2-RABL2B)x1; 0.496 Mb

## Slide 10
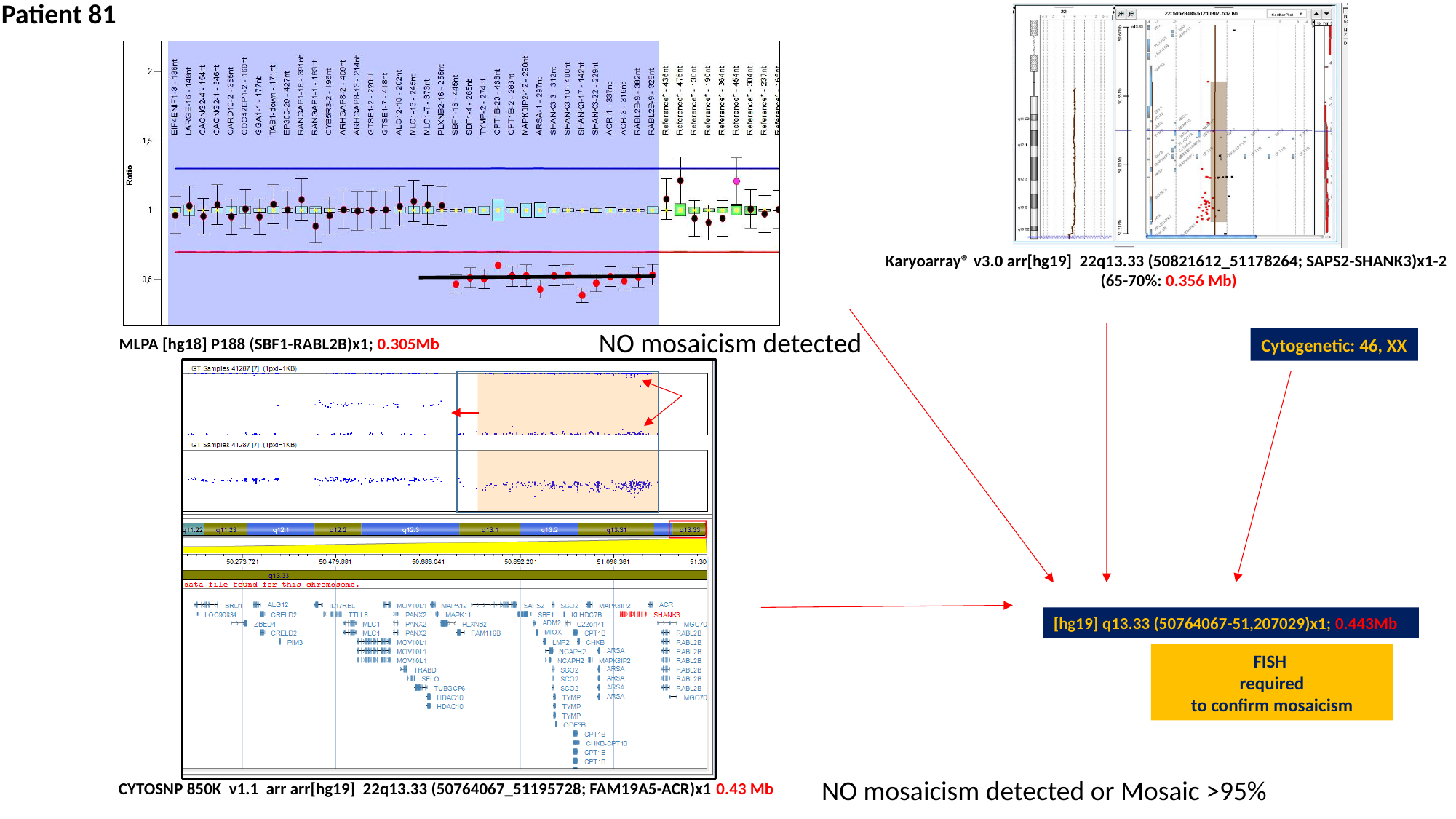

Patient 81
Karyoarray® v3.0 arr[hg19] 22q13.33 (50821612_51178264; SAPS2-SHANK3)x1-2
(65-70%: 0.356 Mb)
NO mosaicism detected
MLPA [hg18] P188 (SBF1-RABL2B)x1; 0.305Mb
Cytogenetic: 46, XX
[hg19] q13.33 (50764067-51,207029)x1; 0.443Mb
FISH
 required
to confirm mosaicism
NO mosaicism detected or Mosaic >95%
 CYTOSNP 850K v1.1 arr arr[hg19] 22q13.33 (50764067_51195728; FAM19A5-ACR)x1 0.43 Mb

## Slide 11
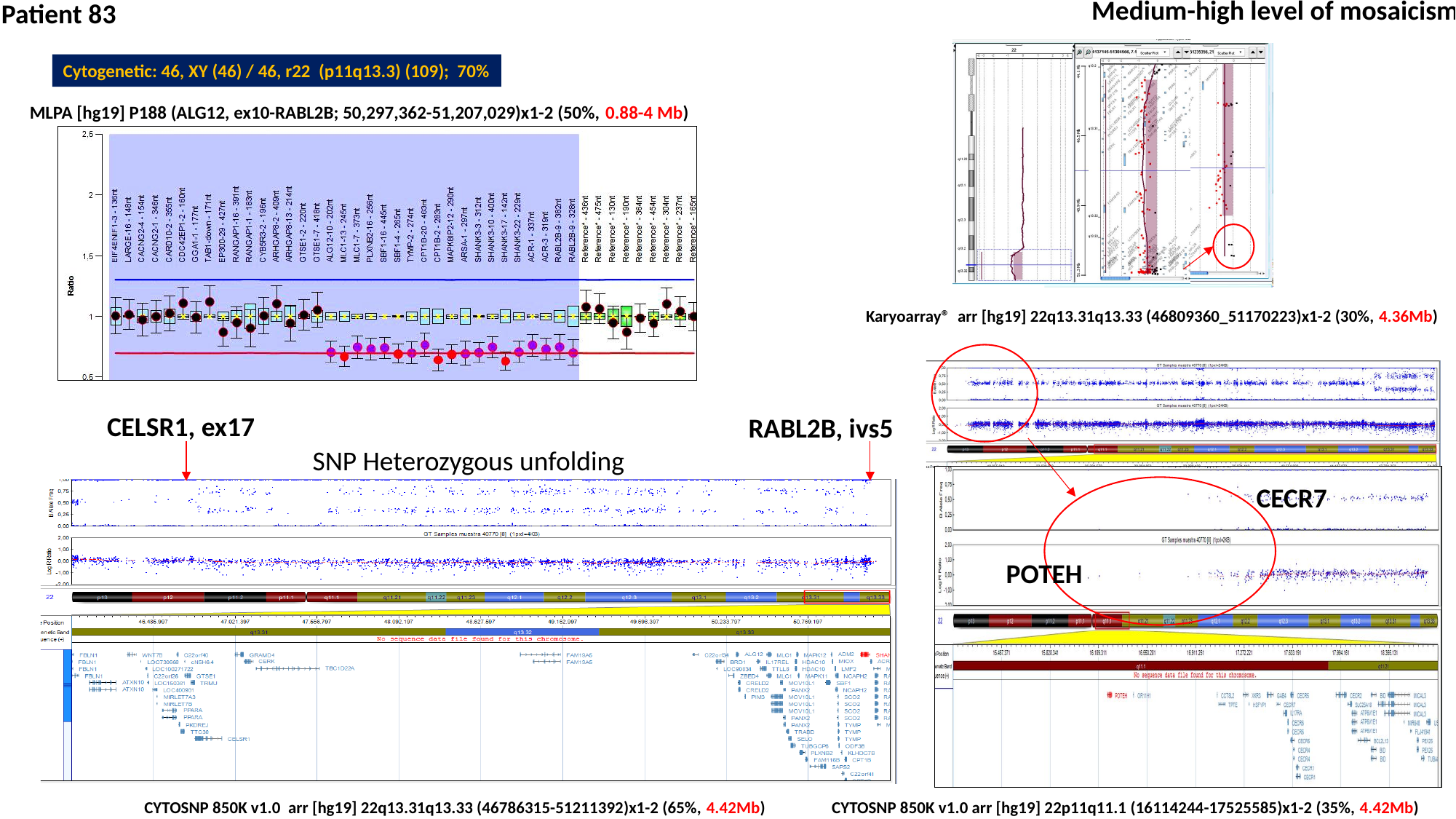

Medium-high level of mosaicism
Patient 83
Cytogenetic: 46, XY (46) / 46, r22 (p11q13.3) (109); 70%
MLPA [hg19] P188 (ALG12, ex10-RABL2B; 50,297,362-51,207,029)x1-2 (50%, 0.88-4 Mb)
Karyoarray® arr [hg19] 22q13.31q13.33 (46809360_51170223)x1-2 (30%, 4.36Mb)
CELSR1, ex17
RABL2B, ivs5
SNP Heterozygous unfolding
CECR7
POTEH
CYTOSNP 850K v1.0 arr [hg19] 22q13.31q13.33 (46786315-51211392)x1-2 (65%, 4.42Mb)
CYTOSNP 850K v1.0 arr [hg19] 22p11q11.1 (16114244-17525585)x1-2 (35%, 4.42Mb)

## Slide 12
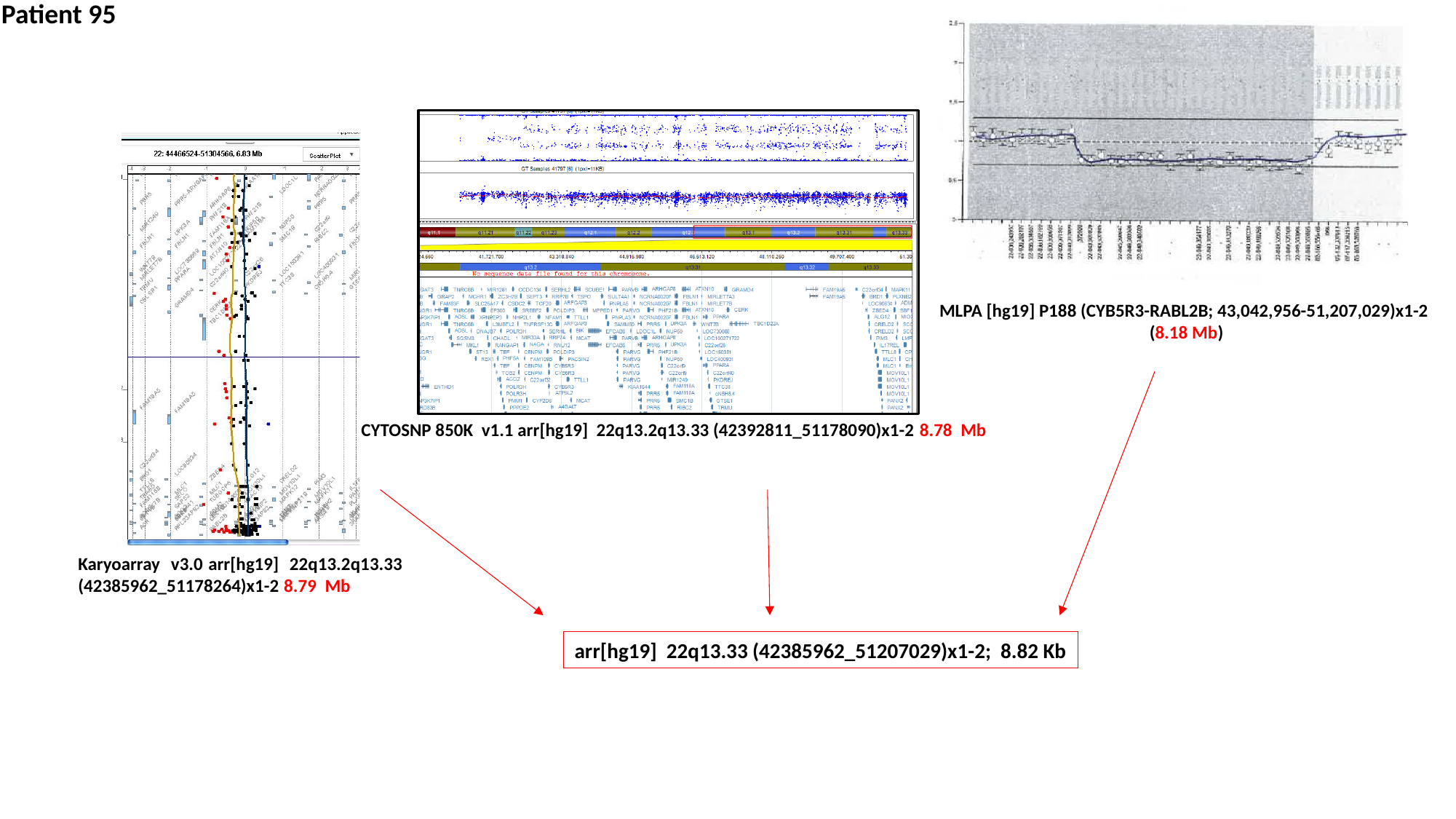

Patient 95
MLPA [hg19] P188 (CYB5R3-RABL2B; 43,042,956-51,207,029)x1-2
(8.18 Mb)
 CYTOSNP 850K v1.1 arr[hg19] 22q13.2q13.33 (42392811_51178090)x1-2 8.78 Mb
Karyoarray v3.0 arr[hg19] 22q13.2q13.33 (42385962_51178264)x1-2 8.79 Mb
arr[hg19] 22q13.33 (42385962_51207029)x1-2; 8.82 Kb

## Slide 13
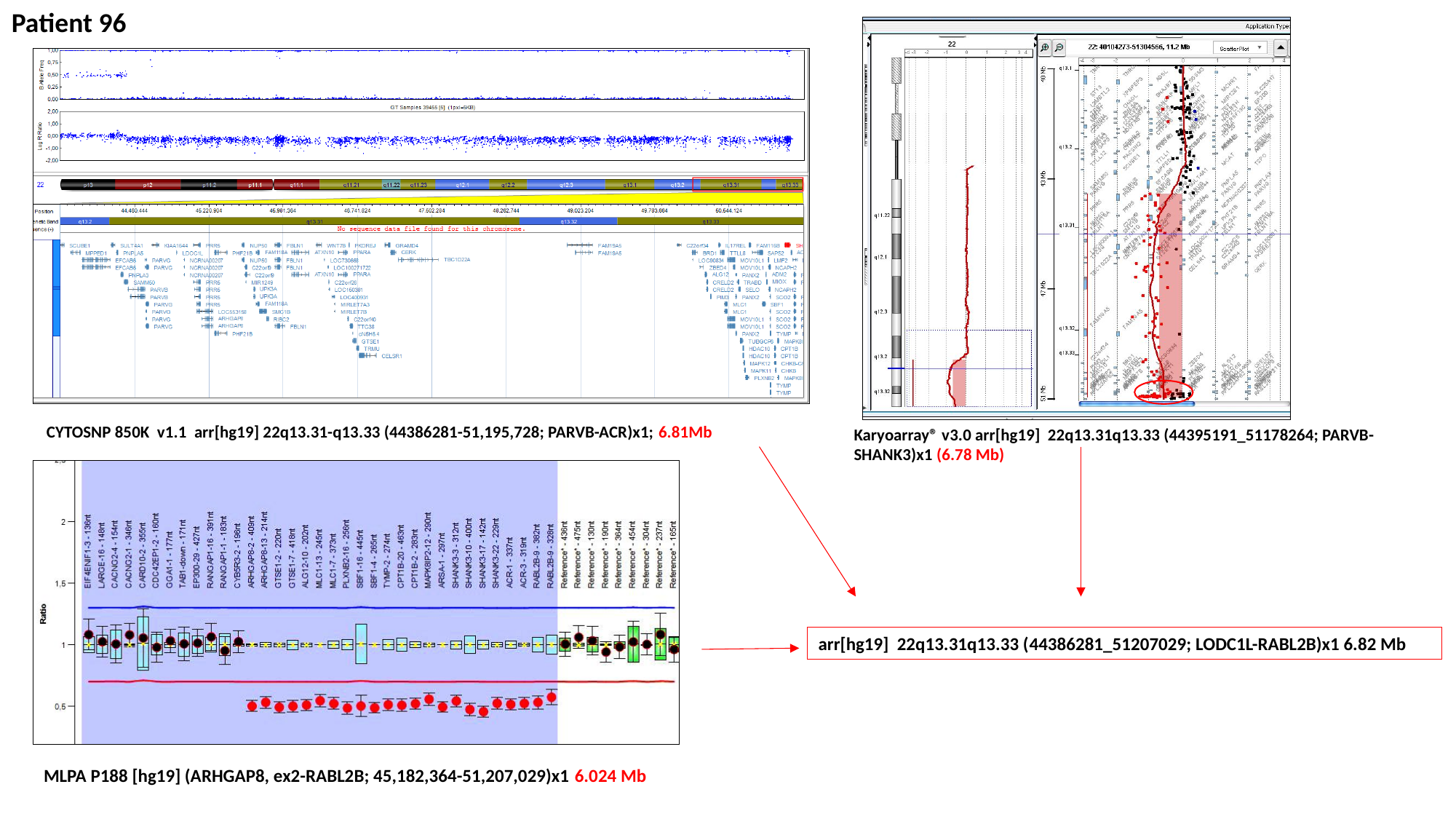

Patient 96
 CYTOSNP 850K v1.1 arr[hg19] 22q13.31-q13.33 (44386281-51,195,728; PARVB-ACR)x1; 6.81Mb
Karyoarray® v3.0 arr[hg19] 22q13.31q13.33 (44395191_51178264; PARVB-SHANK3)x1 (6.78 Mb)
arr[hg19] 22q13.31q13.33 (44386281_51207029; LODC1L-RABL2B)x1 6.82 Mb
MLPA P188 [hg19] (ARHGAP8, ex2-RABL2B; 45,182,364-51,207,029)x1 6.024 Mb

## Slide 14
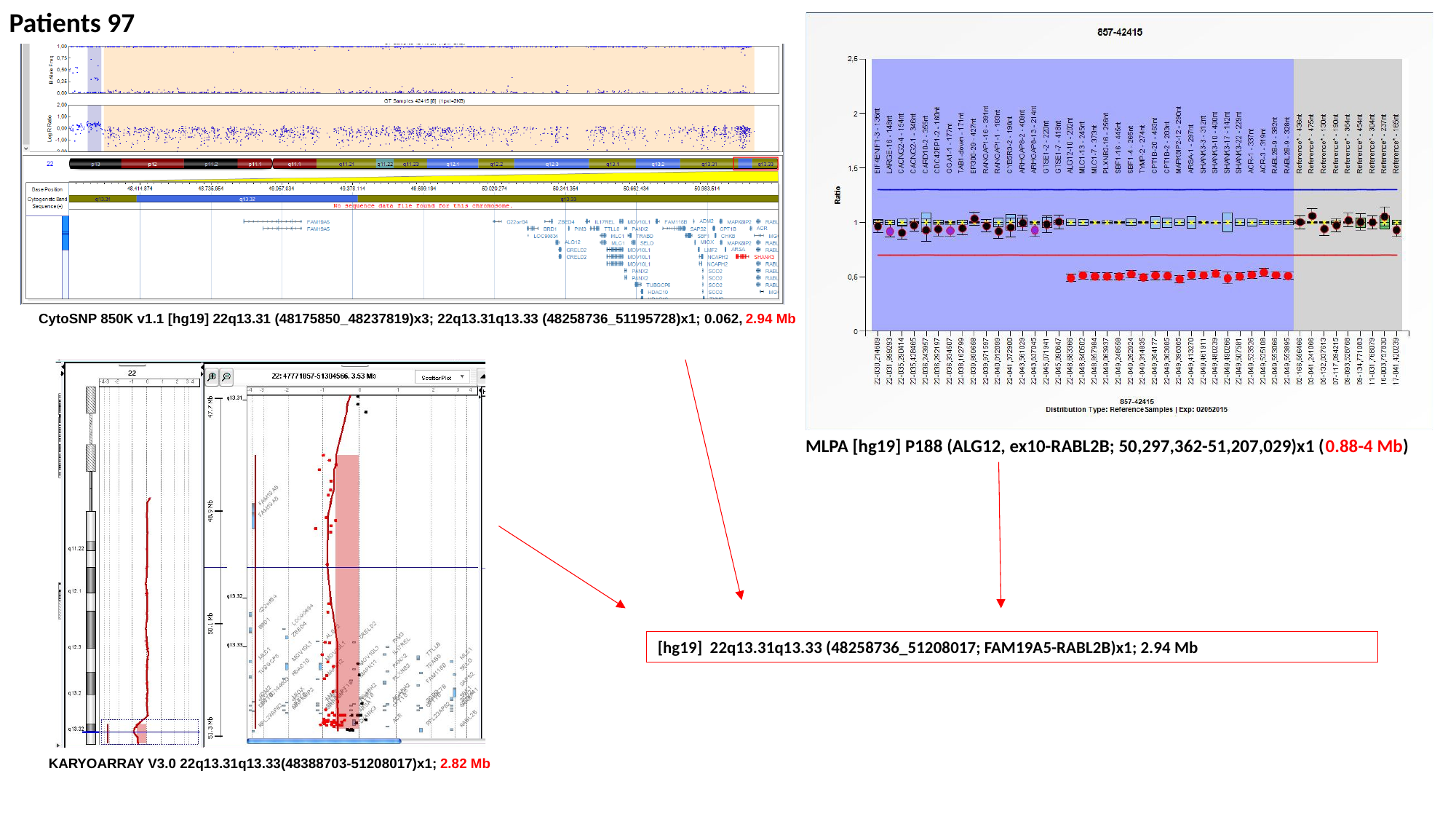

Patients 97
CytoSNP 850K v1.1 [hg19] 22q13.31 (48175850_48237819)x3; 22q13.31q13.33 (48258736_51195728)x1; 0.062, 2.94 Mb
MLPA [hg19] P188 (ALG12, ex10-RABL2B; 50,297,362-51,207,029)x1 (0.88-4 Mb)
[hg19] 22q13.31q13.33 (48258736_51208017; FAM19A5-RABL2B)x1; 2.94 Mb
KARYOARRAY V3.0 22q13.31q13.33(48388703-51208017)x1; 2.82 Mb

## Slide 15
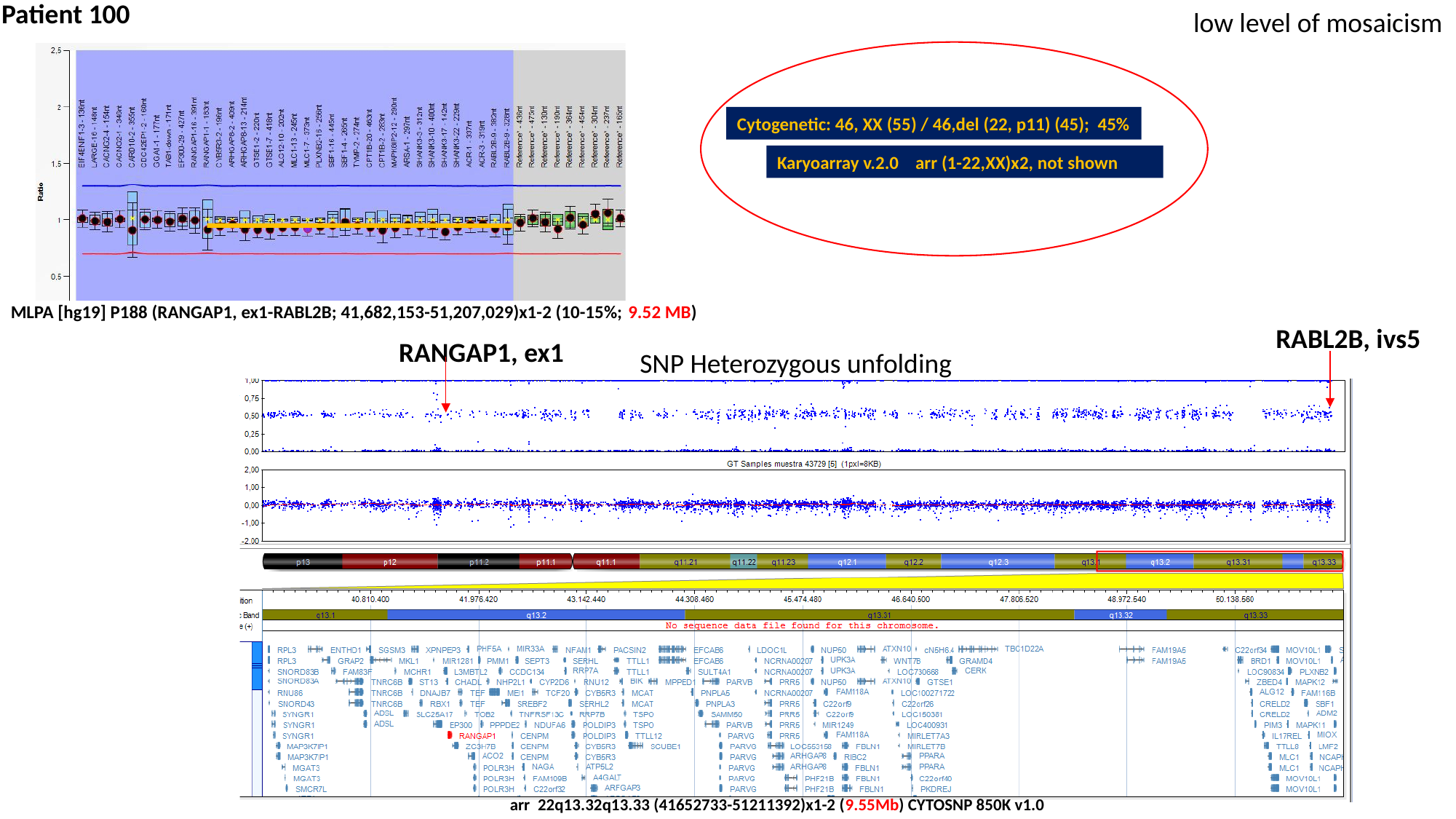

low level of mosaicism
Patient 100
Cytogenetic: 46, XX (55) / 46,del (22, p11) (45); 45%
Karyoarray v.2.0 arr (1-22,XX)x2, not shown
MLPA [hg19] P188 (RANGAP1, ex1-RABL2B; 41,682,153-51,207,029)x1-2 (10-15%; 9.52 MB)
RABL2B, ivs5
RANGAP1, ex1
SNP Heterozygous unfolding
arr 22q13.32q13.33 (41652733-51211392)x1-2 (9.55Mb) CYTOSNP 850K v1.0

## Slide 16
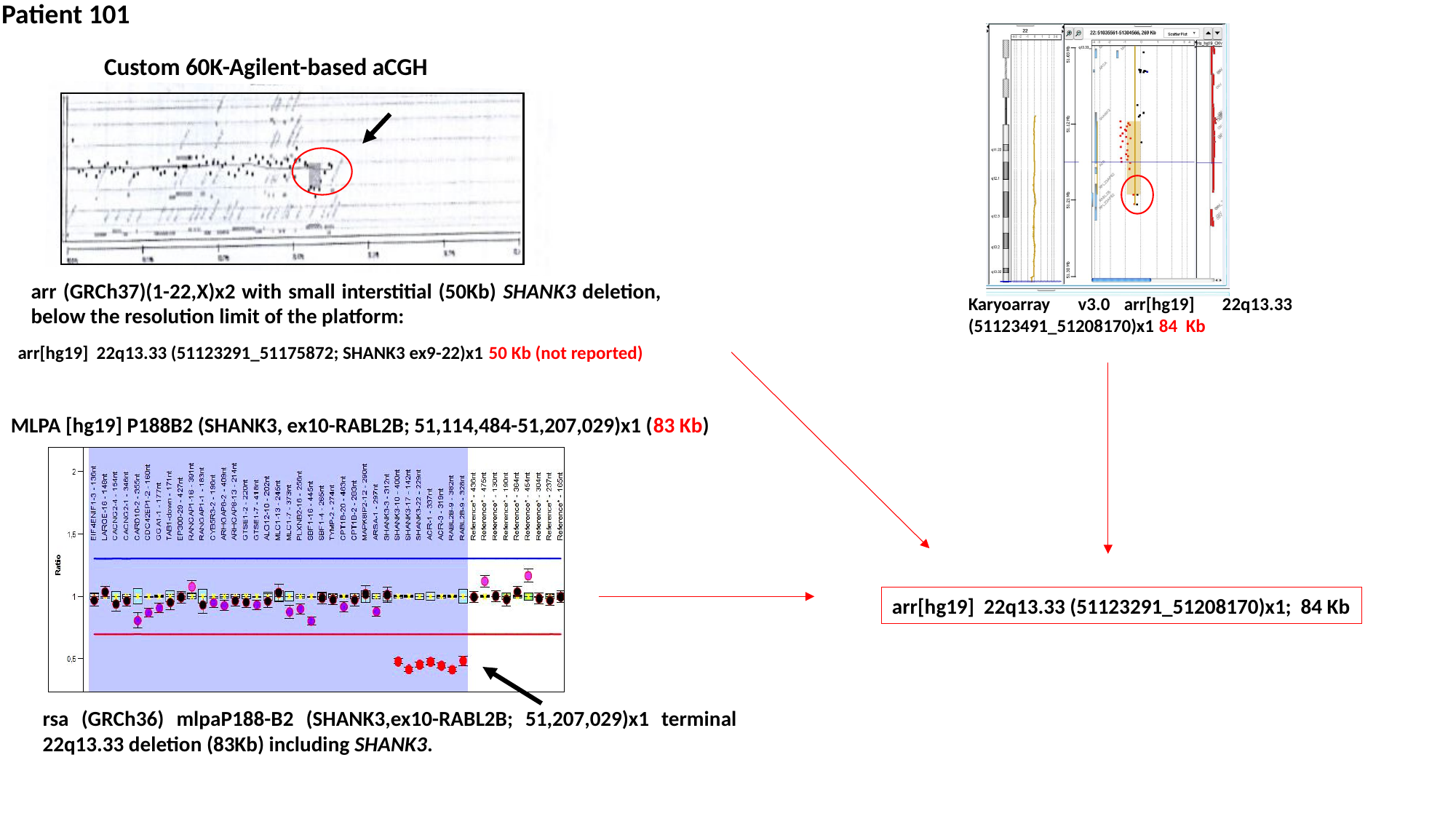

Patient 101
Custom 60K-Agilent-based aCGH
arr (GRCh37)(1-22,X)x2 with small interstitial (50Kb) SHANK3 deletion, below the resolution limit of the platform:
Karyoarray v3.0 arr[hg19] 22q13.33 (51123491_51208170)x1 84 Kb
 arr[hg19] 22q13.33 (51123291_51175872; SHANK3 ex9-22)x1 50 Kb (not reported)
MLPA [hg19] P188B2 (SHANK3, ex10-RABL2B; 51,114,484-51,207,029)x1 (83 Kb)
arr[hg19] 22q13.33 (51123291_51208170)x1; 84 Kb
rsa (GRCh36) mlpaP188-B2 (SHANK3,ex10-RABL2B; 51,207,029)x1 terminal 22q13.33 deletion (83Kb) including SHANK3.

## Slide 17
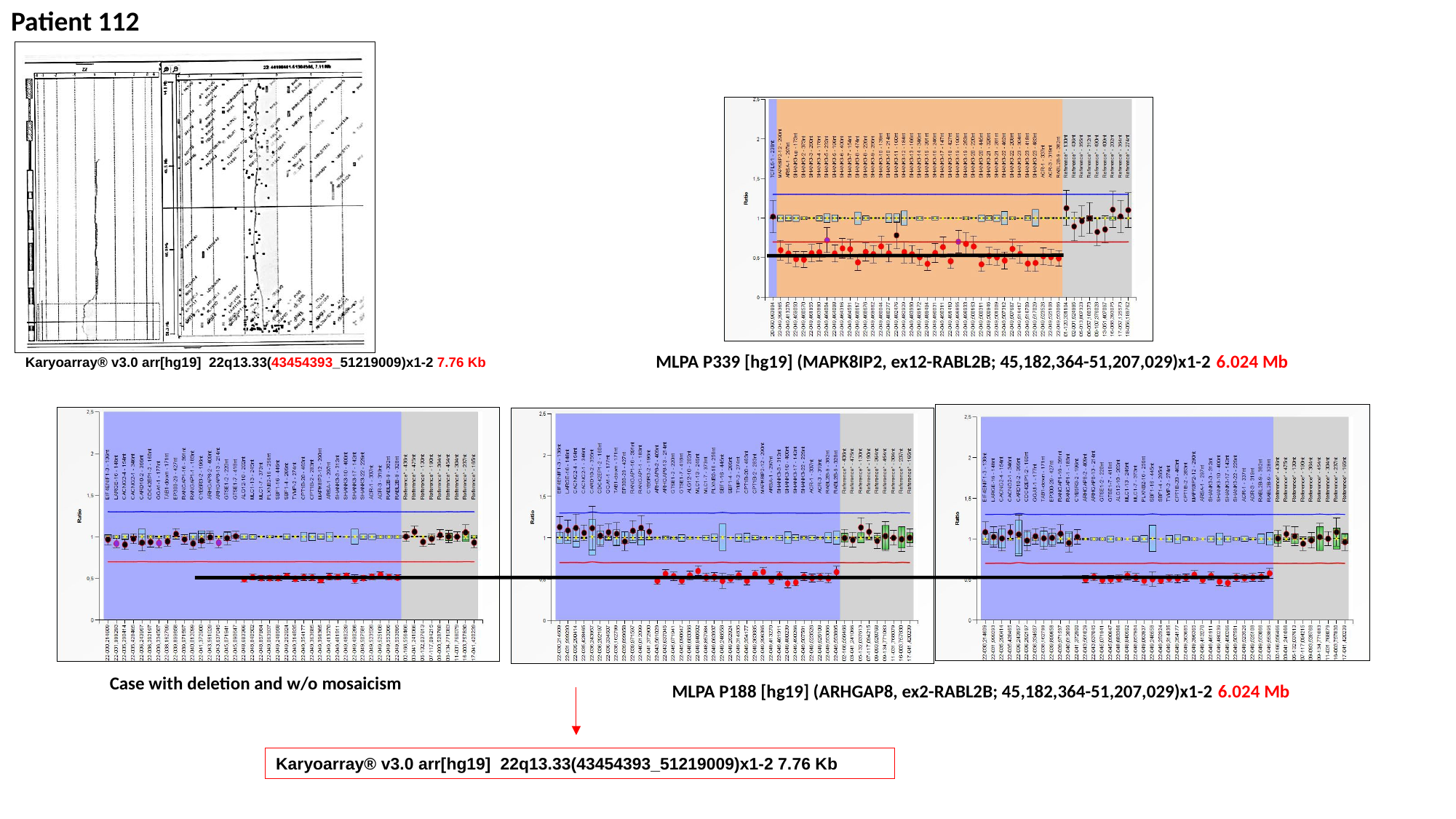

Patient 112
MLPA P339 [hg19] (MAPK8IP2, ex12-RABL2B; 45,182,364-51,207,029)x1-2 6.024 Mb
Karyoarray® v3.0 arr[hg19] 22q13.33(43454393_51219009)x1-2 7.76 Kb
Case with deletion and w/o mosaicism
MLPA P188 [hg19] (ARHGAP8, ex2-RABL2B; 45,182,364-51,207,029)x1-2 6.024 Mb
Karyoarray® v3.0 arr[hg19] 22q13.33(43454393_51219009)x1-2 7.76 Kb

## Slide 18
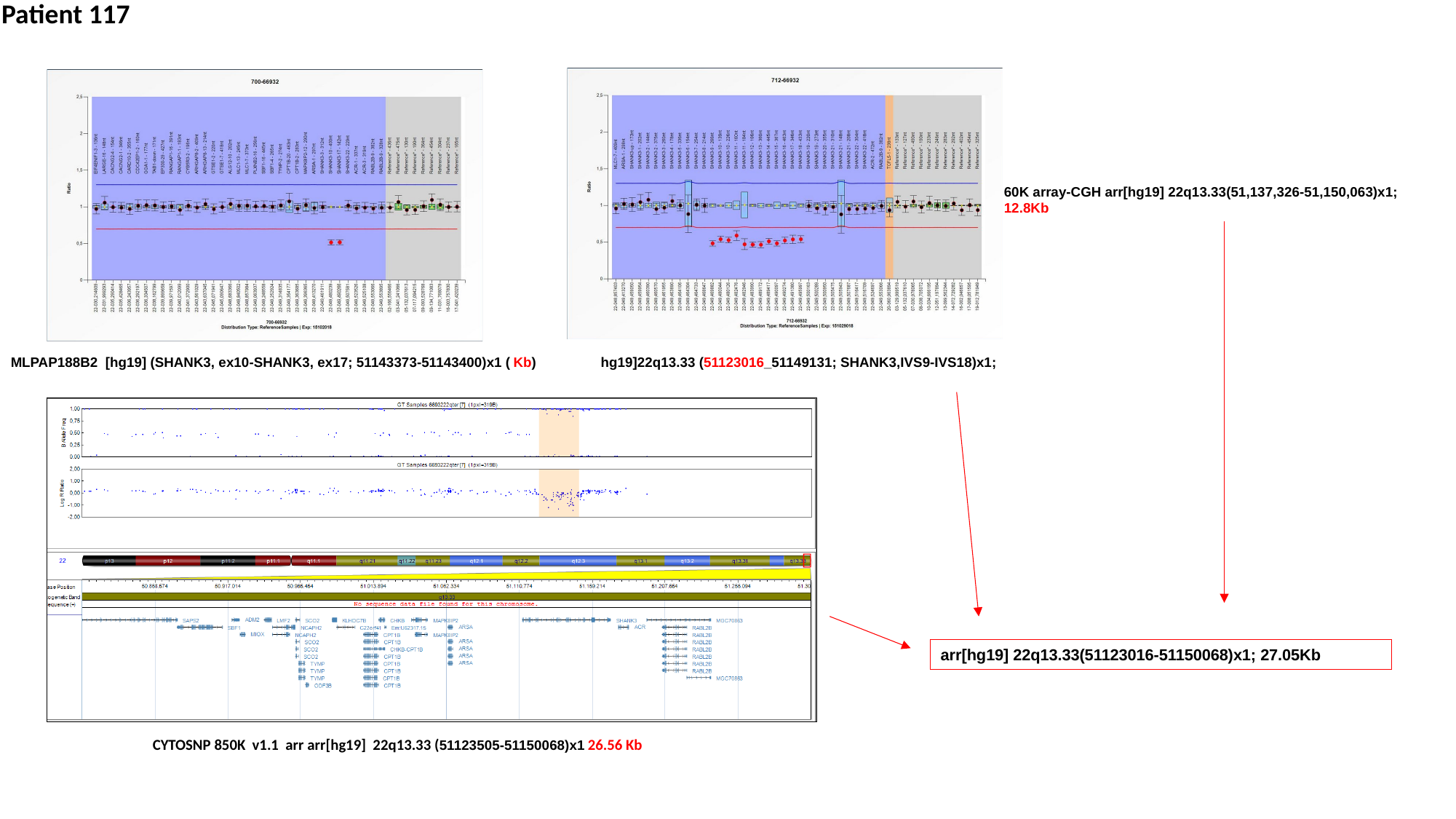

Patient 117
60K array-CGH arr[hg19] 22q13.33(51,137,326-51,150,063)x1; 12.8Kb
MLPAP188B2 [hg19] (SHANK3, ex10-SHANK3, ex17; 51143373-51143400)x1 ( Kb)
hg19]22q13.33 (51123016_51149131; SHANK3,IVS9-IVS18)x1;
arr[hg19] 22q13.33(51123016-51150068)x1; 27.05Kb
CYTOSNP 850K v1.1 arr arr[hg19] 22q13.33 (51123505-51150068)x1 26.56 Kb

## Slide 19
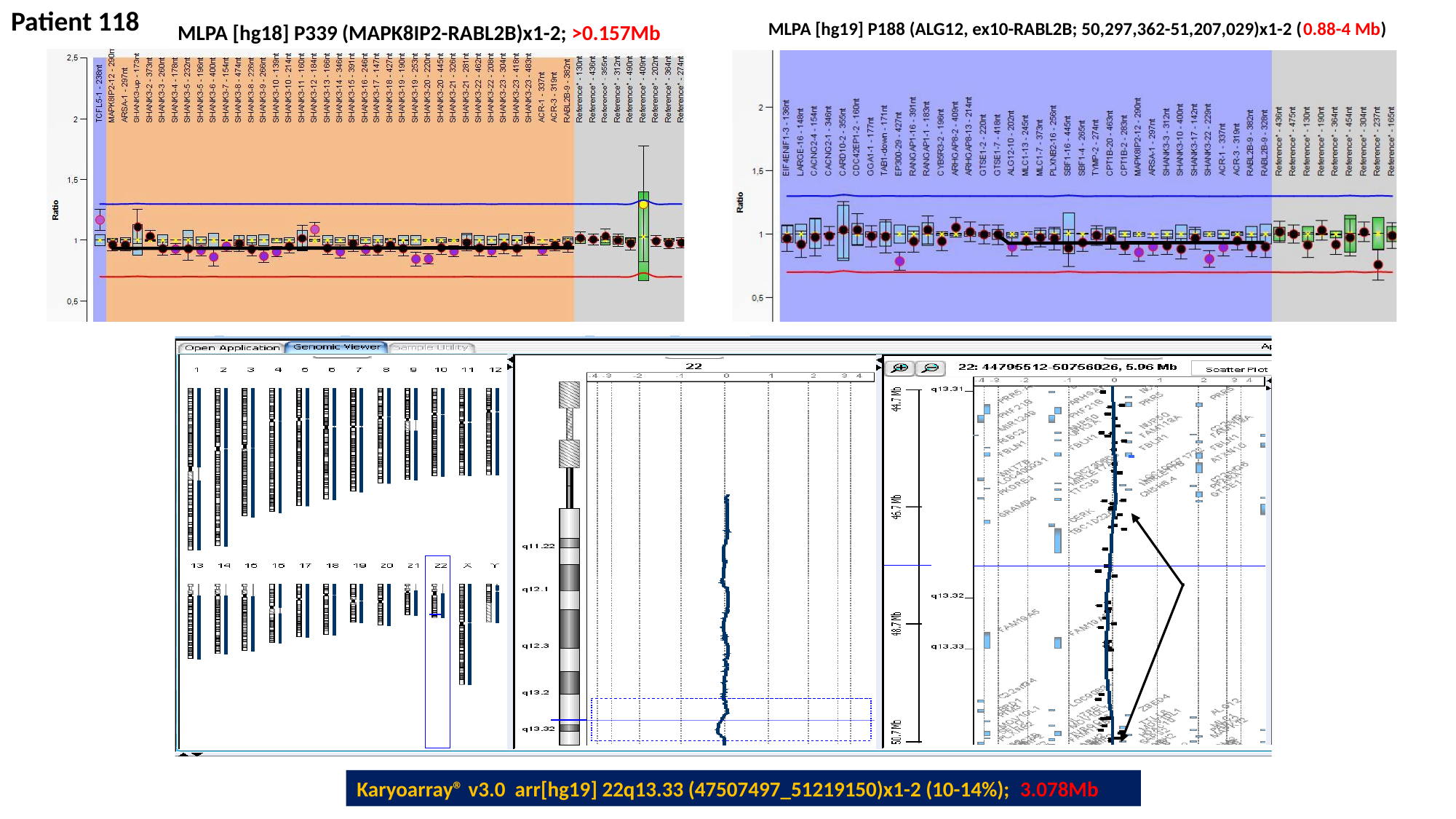

Patient 118
MLPA [hg19] P188 (ALG12, ex10-RABL2B; 50,297,362-51,207,029)x1-2 (0.88-4 Mb)
MLPA [hg18] P339 (MAPK8IP2-RABL2B)x1-2; >0.157Mb
Karyoarray® v3.0 arr[hg19] 22q13.33 (47507497_51219150)x1-2 (10-14%); 3.078Mb

## Slide 20
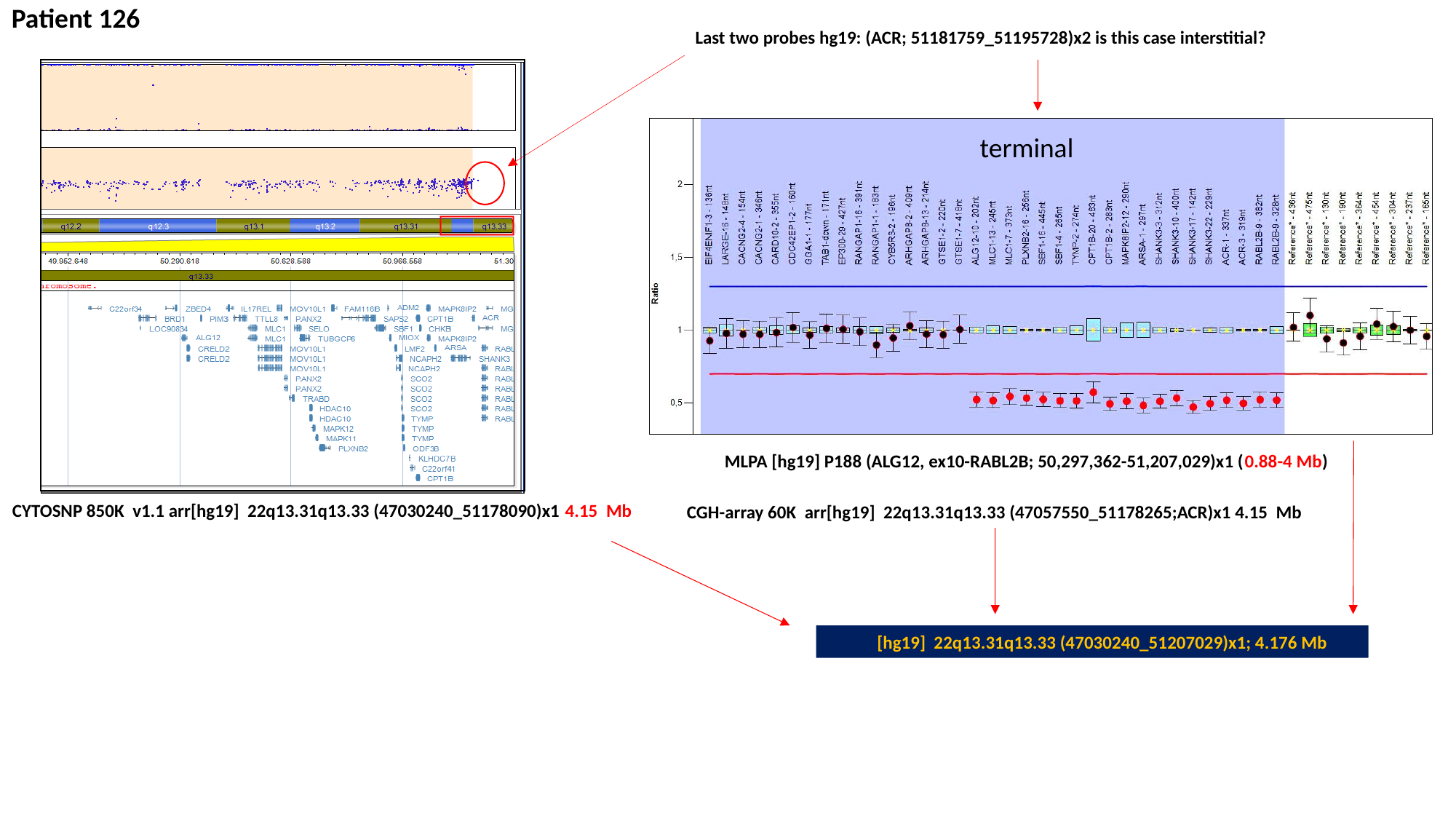

Patient 126
Last two probes hg19: (ACR; 51181759_51195728)x2 is this case interstitial?
terminal
MLPA [hg19] P188 (ALG12, ex10-RABL2B; 50,297,362-51,207,029)x1 (0.88-4 Mb)
 CYTOSNP 850K v1.1 arr[hg19] 22q13.31q13.33 (47030240_51178090)x1 4.15 Mb
CGH-array 60K arr[hg19] 22q13.31q13.33 (47057550_51178265;ACR)x1 4.15 Mb
 [hg19] 22q13.31q13.33 (47030240_51207029)x1; 4.176 Mb

## Slide 21
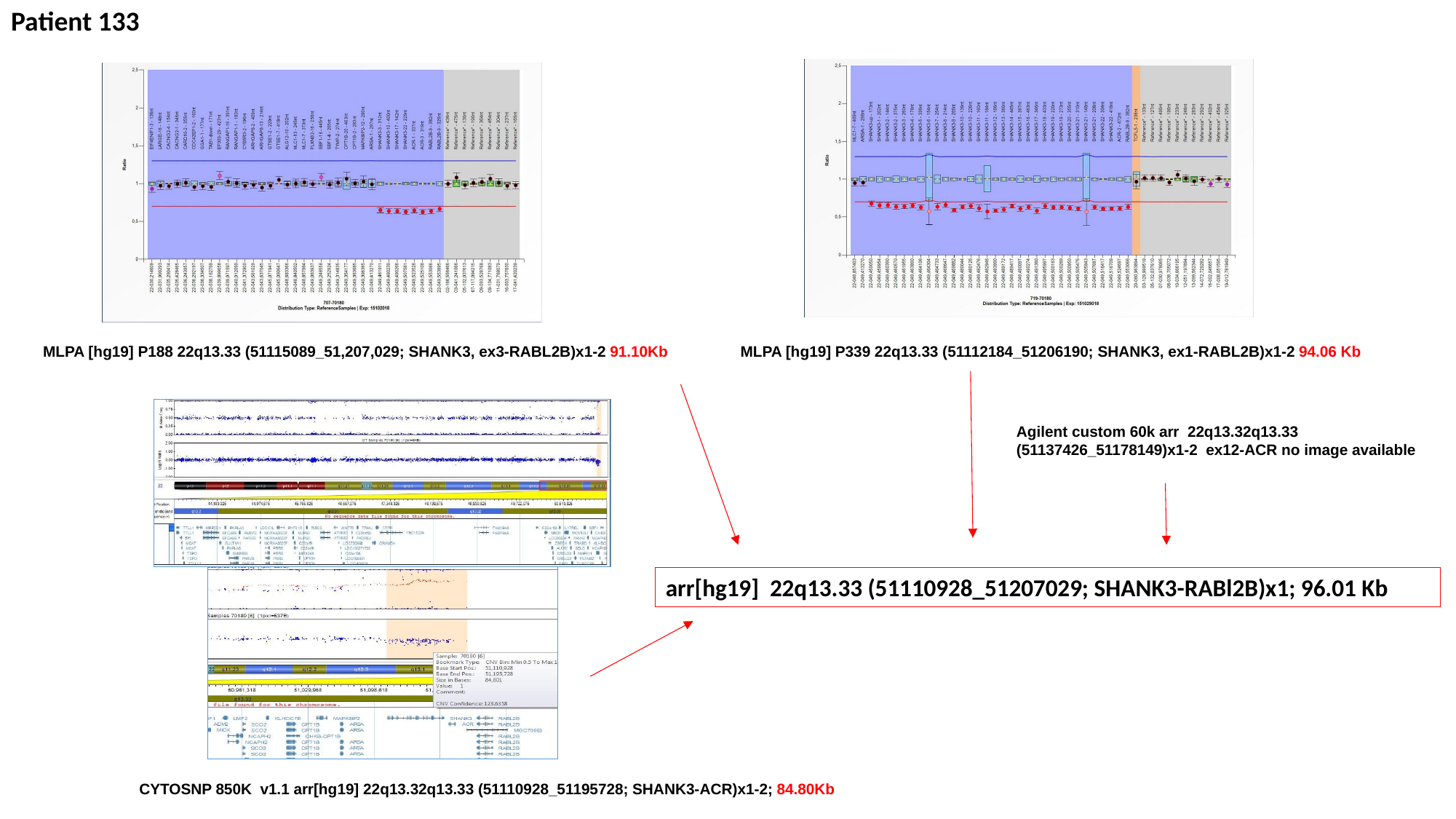

Patient 133
MLPA [hg19] P188 22q13.33 (51115089_51,207,029; SHANK3, ex3-RABL2B)x1-2 91.10Kb
MLPA [hg19] P339 22q13.33 (51112184_51206190; SHANK3, ex1-RABL2B)x1-2 94.06 Kb
Agilent custom 60k arr 22q13.32q13.33 (51137426_51178149)x1-2 ex12-ACR no image available
arr[hg19] 22q13.33 (51110928_51207029; SHANK3-RABl2B)x1; 96.01 Kb
CYTOSNP 850K v1.1 arr[hg19] 22q13.32q13.33 (51110928_51195728; SHANK3-ACR)x1-2; 84.80Kb
