# Supplementary material for: Variability in Phelan-McDermid Syndrome in a Cohort of 210 Individuals
Source: Front Genet. 2022 Apr 12;13:652454. doi: 10.3389/fgene.2022.652454 (PMC9044489; doi:10.3389/fgene.2022.652454)
Supplement: Supplementary file 9 [file Presentation2.PPTX]

## Slide 1
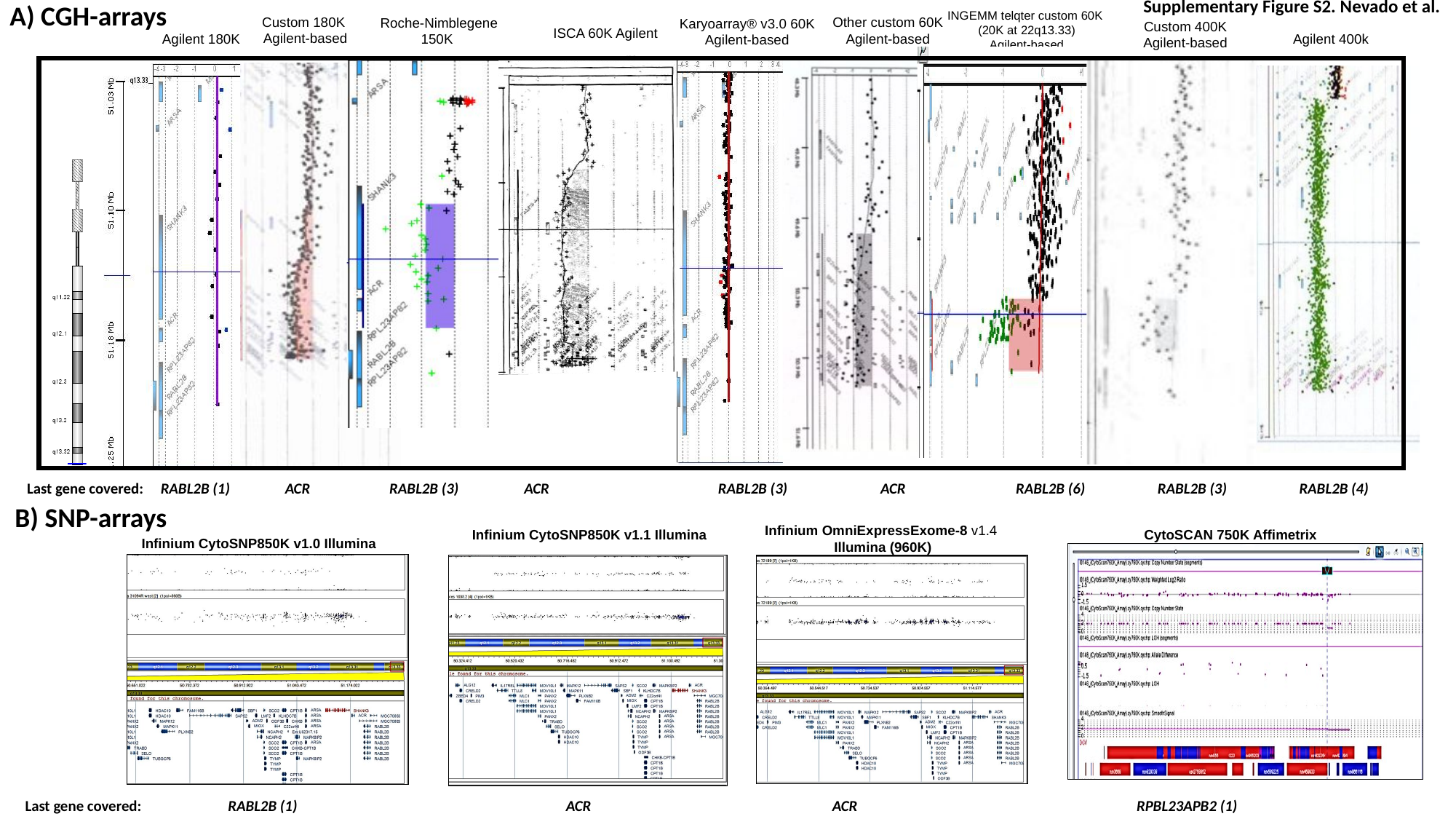

Supplementary Figure S2. Nevado et al.
A) CGH-arrays
INGEMM telqter custom 60K
(20K at 22q13.33)
Agilent-based
Custom 180K
Agilent-based
Other custom 60K
Agilent-based
Roche-Nimblegene
150K
Karyoarray® v3.0 60K
Agilent-based
Custom 400K
Agilent-based
ISCA 60K Agilent
Agilent 180K
Agilent 400k
Last gene covered: RABL2B (1) ACR RABL2B (3) ACR RABL2B (3) ACR RABL2B (6) RABL2B (3) RABL2B (4)
B) SNP-arrays
Infinium OmniExpressExome-8 v1.4
Illumina (960K)
Infinium CytoSNP850K v1.1 Illumina
CytoSCAN 750K Affimetrix
Infinium CytoSNP850K v1.0 Illumina
Last gene covered: RABL2B (1) ACR ACR RPBL23APB2 (1)
