# Supplementary material for: Variability in Phelan-McDermid Syndrome in a Cohort of 210 Individuals
Source: Front Genet. 2022 Apr 12;13:652454. doi: 10.3389/fgene.2022.652454 (PMC9044489; doi:10.3389/fgene.2022.652454)
Supplement: Supplementary file 11 [file Presentation7.PPTX]

## Slide 1
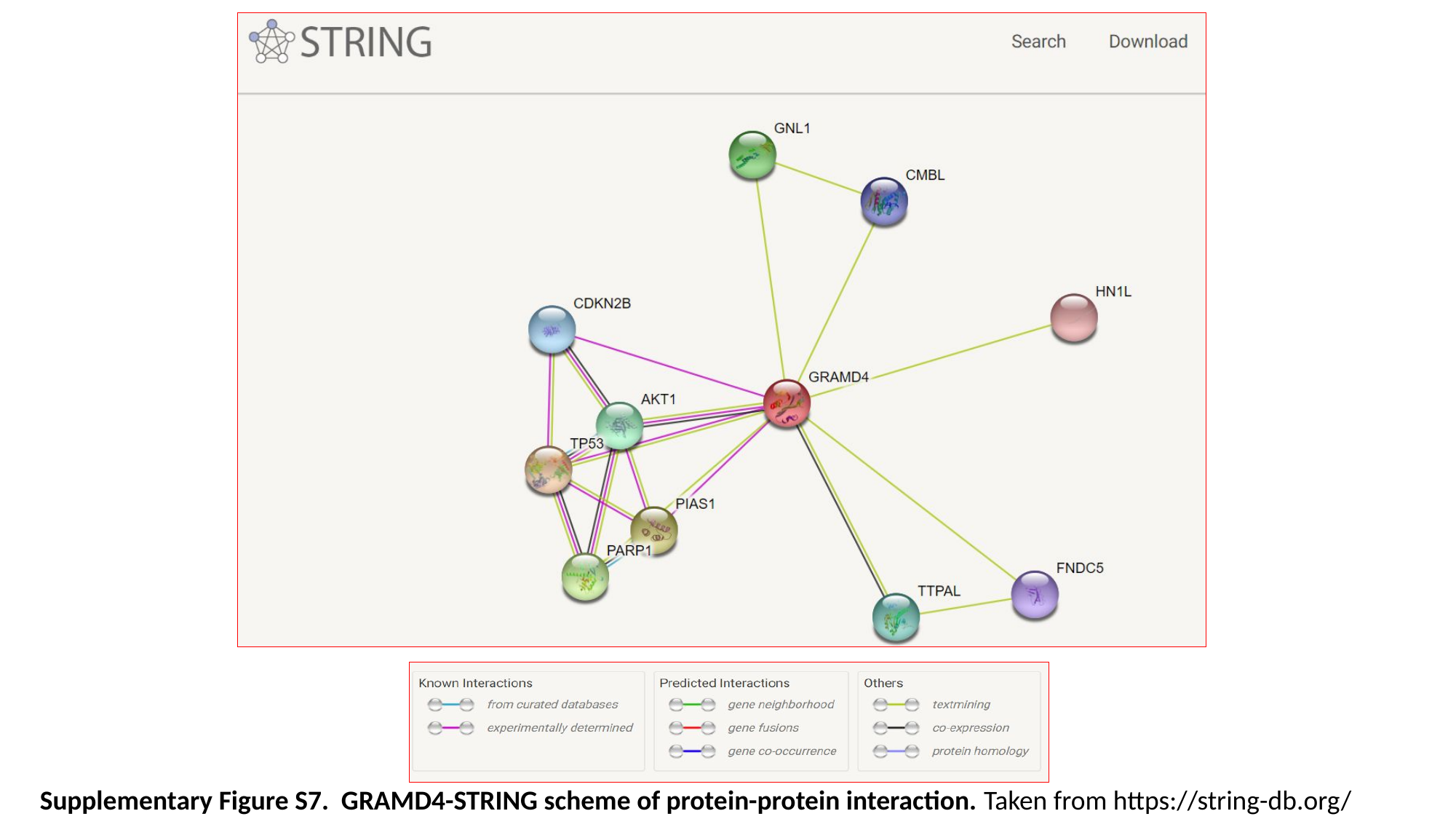

Supplementary Figure S7. GRAMD4-STRING scheme of protein-protein interaction. Taken from https://string-db.org/
