# Supplementary material for: Variability in Phelan-McDermid Syndrome in a Cohort of 210 Individuals
Source: Front Genet. 2022 Apr 12;13:652454. doi: 10.3389/fgene.2022.652454 (PMC9044489; doi:10.3389/fgene.2022.652454)
Supplement: Supplementary file 12 [file Presentation5.PPTX]

## Slide 1
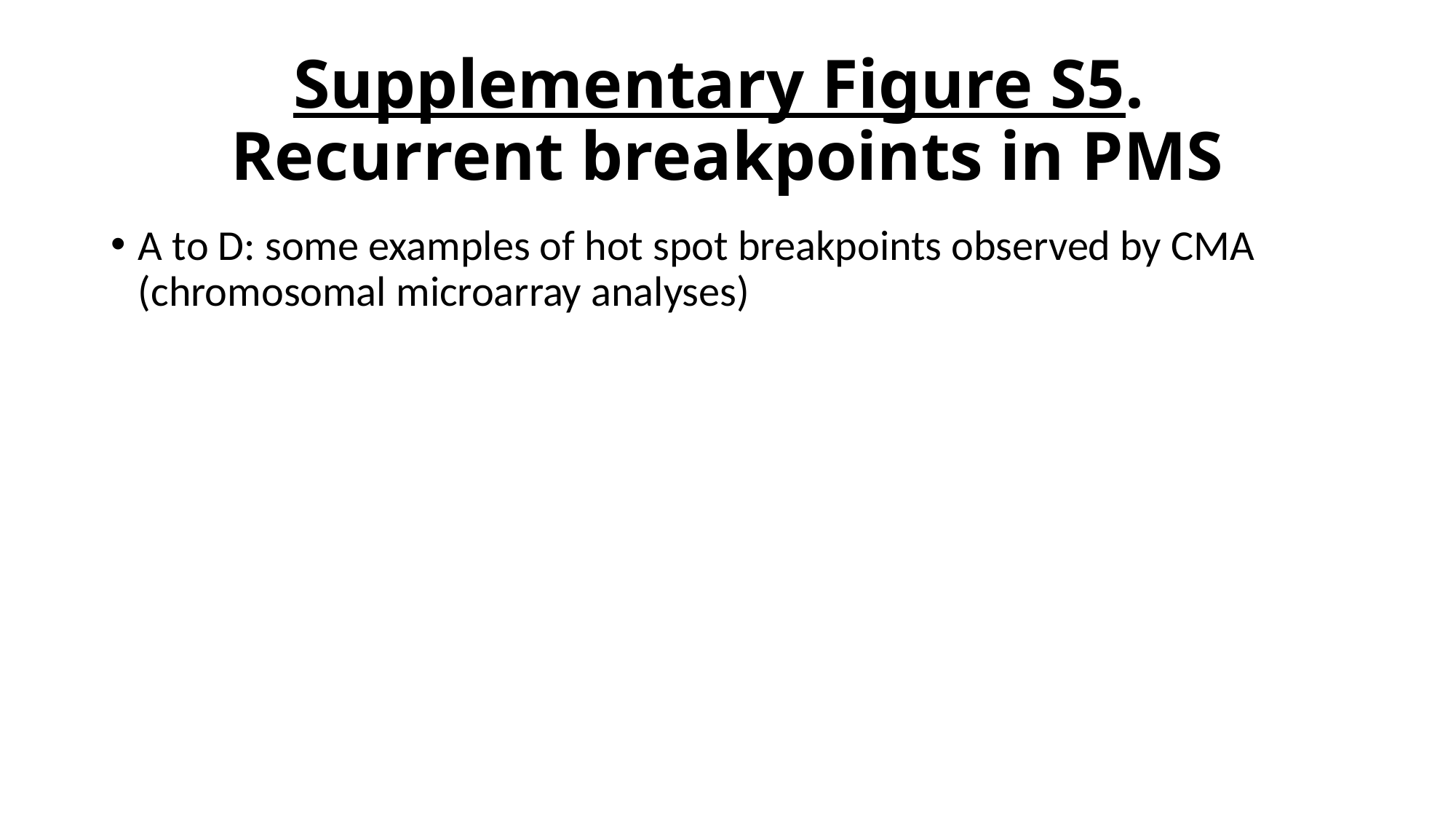

# Supplementary Figure S5. Recurrent breakpoints in PMS
A to D: some examples of hot spot breakpoints observed by CMA (chromosomal microarray analyses)

## Slide 2
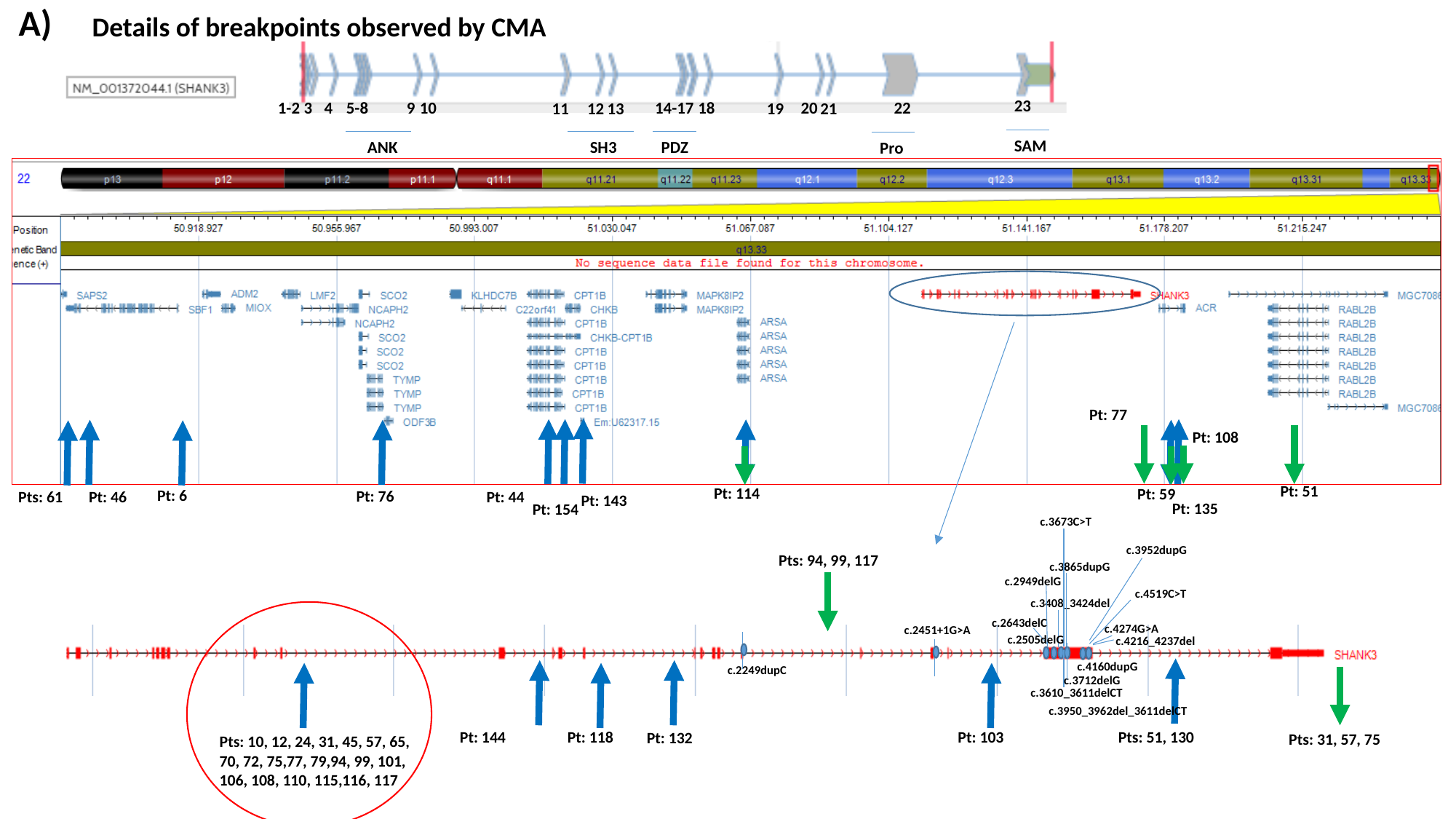

A)
Details of breakpoints observed by CMA
23
3
4
10
5-8
9
14-17
18
22
1-2
20
13
11
12
19
21
SAM
ANK
SH3
PDZ
Pro
Pt: 77
Pt: 108
Pt: 51
Pt: 114
Pt: 59
Pt: 6
Pt: 76
Pts: 61
Pt: 46
Pt: 44
Pt: 143
Pt: 135
Pt: 154
c.3673C>T
c.3952dupG
Pts: 94, 99, 117
c.3865dupG
c.2949delG
c.4519C>T
c.3408_3424del
c.2643delC
c.4274G>A
c.2451+1G>A
c.2505delG
c.4216_4237del
c.4160dupG
c.2249dupC
c.3712delG
c.3610_3611delCT
c.3950_3962del_3611delCT
Pt: 144
Pt: 118
Pt: 103
Pts: 51, 130
Pt: 132
Pts: 31, 57, 75
Pts: 10, 12, 24, 31, 45, 57, 65, 70, 72, 75,77, 79,94, 99, 101, 106, 108, 110, 115,116, 117

## Slide 3
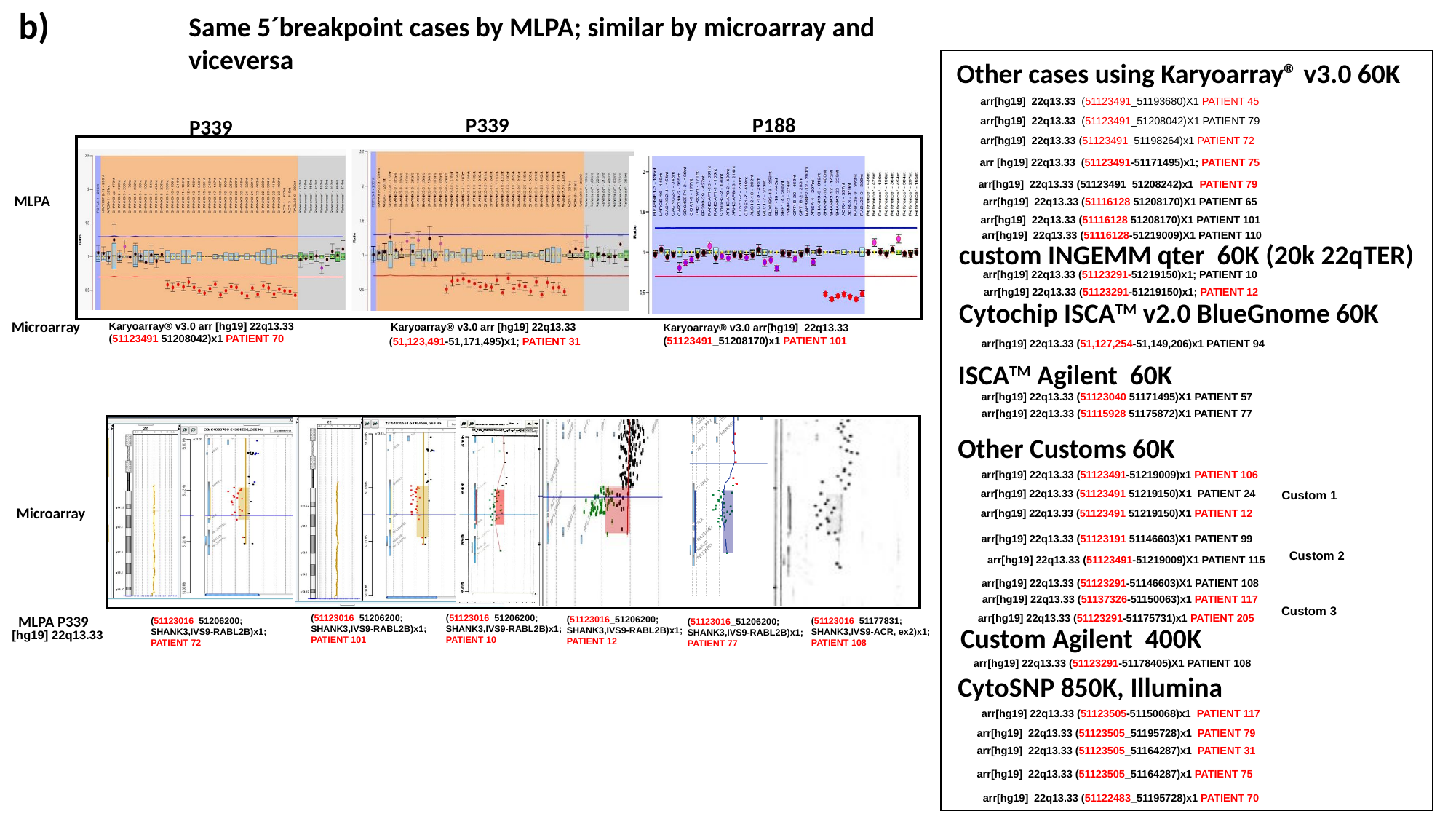

b)
Same 5´breakpoint cases by MLPA; similar by microarray and viceversa
Other cases using Karyoarray® v3.0 60K
arr[hg19] 22q13.33 (51123491_51193680)X1 PATIENT 45
P339
P188
P339
arr[hg19] 22q13.33 (51123491_51208042)X1 PATIENT 79
arr[hg19] 22q13.33 (51123491_51198264)x1 PATIENT 72
arr [hg19] 22q13.33 (51123491-51171495)x1; PATIENT 75
arr[hg19] 22q13.33 (51123491_51208242)x1 PATIENT 79
MLPA
arr[hg19] 22q13.33 (51116128 51208170)X1 PATIENT 65
arr[hg19] 22q13.33 (51116128 51208170)X1 PATIENT 101
arr[hg19] 22q13.33 (51116128-51219009)X1 PATIENT 110
custom INGEMM qter 60K (20k 22qTER)
arr[hg19] 22q13.33 (51123291-51219150)x1; PATIENT 10
arr[hg19] 22q13.33 (51123291-51219150)x1; PATIENT 12
Cytochip ISCATM v2.0 BlueGnome 60K
Microarray
Karyoarray® v3.0 arr [hg19] 22q13.33 (51123491 51208042)x1 PATIENT 70
Karyoarray® v3.0 arr [hg19] 22q13.33
(51,123,491-51,171,495)x1; PATIENT 31
Karyoarray® v3.0 arr[hg19] 22q13.33
(51123491_51208170)x1 PATIENT 101
arr[hg19] 22q13.33 (51,127,254-51,149,206)x1 PATIENT 94
ISCATM Agilent 60K
arr[hg19] 22q13.33 (51123040 51171495)X1 PATIENT 57
arr[hg19] 22q13.33 (51115928 51175872)X1 PATIENT 77
Other Customs 60K
arr[hg19] 22q13.33 (51123491-51219009)x1 PATIENT 106
arr[hg19] 22q13.33 (51123491 51219150)X1 PATIENT 24
Custom 1
Microarray
arr[hg19] 22q13.33 (51123491 51219150)X1 PATIENT 12
arr[hg19] 22q13.33 (51123191 51146603)X1 PATIENT 99
Custom 2
arr[hg19] 22q13.33 (51123491-51219009)X1 PATIENT 115
arr[hg19] 22q13.33 (51123291-51146603)X1 PATIENT 108
arr[hg19] 22q13.33 (51137326-51150063)x1 PATIENT 117
Custom 3
arr[hg19] 22q13.33 (51123291-51175731)x1 PATIENT 205
MLPA P339
(51123016_51206200;
SHANK3,IVS9-RABL2B)x1; PATIENT 101
(51123016_51206200;
SHANK3,IVS9-RABL2B)x1; PATIENT 10
(51123016_51206200;
SHANK3,IVS9-RABL2B)x1; PATIENT 12
(51123016_51206200;
SHANK3,IVS9-RABL2B)x1; PATIENT 72
(51123016_51177831;
SHANK3,IVS9-ACR, ex2)x1; PATIENT 108
(51123016_51206200;
SHANK3,IVS9-RABL2B)x1; PATIENT 77
Custom Agilent 400K
[hg19] 22q13.33
arr[hg19] 22q13.33 (51123291-51178405)X1 PATIENT 108
CytoSNP 850K, Illumina
arr[hg19] 22q13.33 (51123505-51150068)x1 PATIENT 117
arr[hg19] 22q13.33 (51123505_51195728)x1 PATIENT 79
arr[hg19] 22q13.33 (51123505_51164287)x1 PATIENT 31
arr[hg19] 22q13.33 (51123505_51164287)x1 PATIENT 75
arr[hg19] 22q13.33 (51122483_51195728)x1 PATIENT 70

## Slide 4
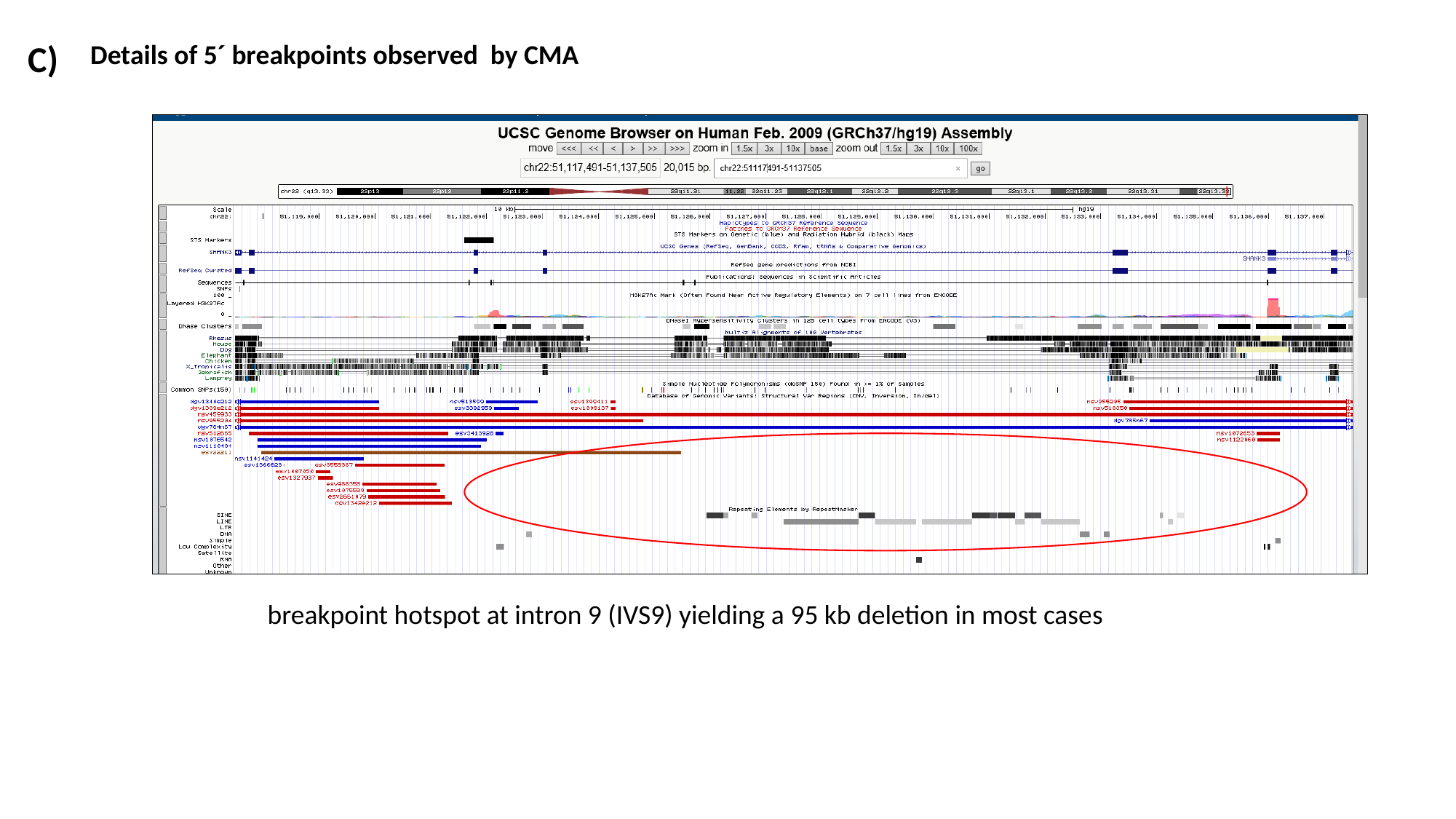

C)
Details of 5´ breakpoints observed by CMA
 breakpoint hotspot at intron 9 (IVS9) yielding a 95 kb deletion in most cases

## Slide 5
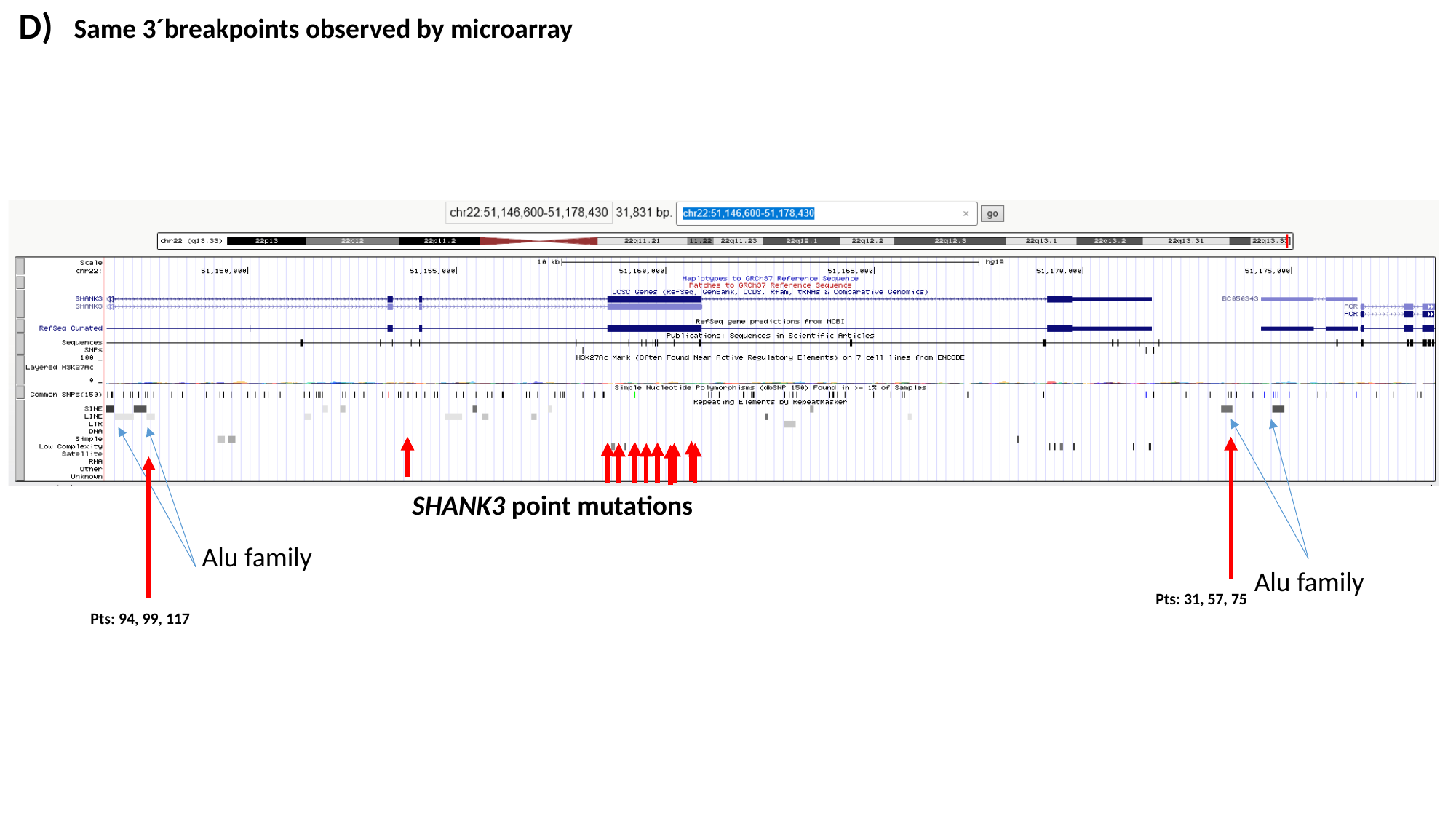

D)
Same 3´breakpoints observed by microarray
SHANK3 point mutations
Alu family
Alu family
Pts: 31, 57, 75
Pts: 94, 99, 117
